# Enhanced One‐Pot Dual‐CRISPR‐Based Assay Lyophilized on a 3D‐Printed Disc for Field‐Deployable Multiplex Bacteria Detection

**DOI:** 10.1002/advs.202509355

**Published:** 2025-09-16

**Authors:** Yuqing Shen, Bo Lu, Biao Ma, Xiong Ding

**Affiliations:** ^1^ Key Laboratory of Environmental Medicine and Engineering Ministry of Education School of Public Health Department of Nutrition and Food Hygiene School of Public Health Southeast University Nanjing 210009 P. R. China; ^2^ State Key Laboratory of Digital Medical Engineering School of Biological Science and Medical Engineering Southeast University Nanjing 210009 P. R. China

**Keywords:** 3D‐printed microfluidic disc, EOD‐CRISPR, field‐deployable multiplex detection, onsite CRISPR‐based detection, foodborne bacteria

## Abstract

One‐pot CRISPR‐based detection combining recombinase polymerase amplification (RPA) enables rapid and accurate nucleic acid testing but faces challenges in performance, multiplexing, and field‐ready lyophilization. Here, an enhanced one‐pot, helicase‐assisted RPA (hRPA)‐combined, dual‐CRISPR/uAsCas12a (EOD‐CRISPR) assay is described which can be lyophilized on a 3D‐printed microfluidic disc to achieve field‐deployable multiplex bacteria detection. In EOD‐CRISPR reactions, the reaction speed, sensitivity, and fluorescence signal are significantly enhanced due to the synergistic effect of bovine serum albumin, hRPA, and uAsCas12a nuclease. The 3D‐printed disc features four central chambers encircled by eight outer chambers, permitting detecting four targets simultaneously. For stable lyophilization on disc chambers, glassfiber membranes are inserted as substrates to adsorb the EOD‐CRISPR reagents containing a protectant of 5% trehalose and 1% glycine. Toward point‐of‐need testing, EOD‐CRISPR‐lyophilized discs are applied to build an onsite detection platform. Through detecting synthetic food samples contaminated by four foodborne bacteria (i.e., *Bacillus cereus*, *Salmonella enterica*, *Staphylococcus aureus*, and *Escherichia coli* O157:H7), the onsite detection platform is validated and the sensitivity (80%–88.9%) and specificity (92.3%–100%) are comparable to those of standard PCR methods. Therefore, the field‐deployable multiplex EOD‐CRISPR assays holds great potentials for onsite bacteria detection and beyond.

## Graphical abstract

An enhanced one‐pot, helicase‐assisted recombinase polymerase amplification (hRPA)‐combined, dual‐CRISPR/uAsCas12a assay is developed and lyophilized on a 3D‐printed microfluidic disc to achieve field‐deployable multiplex bacteria detection.

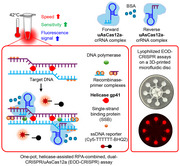

## 1 Introduction

The CRISPR‐based detection has emerged as a next‐generation molecular diagnostic technology, due to the programmable precision of Cas proteins and target‐specific guide RNA for nucleic acid recognition.^[^ ^1^ ^]^ Currently, most CRISPR‐based detection methods leverage CRISPR/Cas systems which possess *trans*‐cleavage activity, for instance, CRISPR/Cas12^[^ ^2^ ^]^ and CRISPR/Cas13.^[^ ^3^ ^]^ However, CRISPR‐based detection approaches solely depending on *trans*‐cleavage activity perform low sensitivity.^[^ ^4^ ^]^ Thus, combining nucleic acid amplification such as polymerase chain reaction (PCR) and isothermal nucleic acid amplification (INAA) is indispensable for high‐performance detection.^[^ ^5^ ^]^ Whereas, INAA is advantageous over PCR toward onsite or point‐of‐need detection, attributed to its superior performance in speed, simplicity, and sensitivity.^[^ ^6^ ^]^

As of now, INAA‐combined CRISPR‐based detection (INAA‐CRISPR) methods have been applied to detect pathogens,^[^ ^7^ ^]^ genetic mutation biomarkers,^[^ ^8^ ^]^ and even non‐biological molecules.^[^ ^9^ ^]^ Among diverse INAA techniques, loop‐mediated isothermal amplification (LAMP) and recombinase polymerase amplification (RPA) are commonly used to develop two‐step or one‐pot INAA‐CRISPR. In two‐step detection, LAMP/RPA servers as the first step to pre‐amplify targets, followed by the CRISPR‐based detection of amplicons.^[^ ^5^, ^10^ ^]^ Although possessing attomolar sensitivity, two‐step detection involves pipetting amplicons, prone to causing aerosol contaminations. In contrast, one‐pot format combines LAMP/RPA and CRISPR‐based detection in one solution, greatly simplifying procedures and reducing aerosol contaminations.

Presently, a variety of one‐pot LAMP/RPA‐CRISPR methods have been reported, such as All‐In‐One Dual CRISPR‐Cas12a,^[^ ^11^ ^]^ STOPCovid, Version 2,^[^ ^12^ ^]^ CRISPR‐based Diagnostic,^[^ ^13^ ^]^ and CRISPR Single Pot Assay for Detecting Emerging Variants of Concern.^[^ ^14^ ^]^ Since LAMP usually initiates at elevated temperature of 60–65 °C,^[^ ^15^ ^]^ one‐pot LAMP‐CRISPR entails thermophilic CRISPR/Cas systems (e.g., CRISPR/BrCas12b,^[^ ^14^ ^]^ CRISPR/AapCas12b,^[^ ^12^ ^]^ and CRISPR/TccCas13a^[^ ^16^ ^]^) to ensure detection efficiency. However, these thermophilic Cas nucleases function with long guide RNA (over 100 nt) or depending on RNA transcription, complicating optimization of reaction systems. Accordingly, one‐pot assays employing RPA (usually at 37–42 °C) and mesophilic CRISPR/Cas systems (e.g., CRISPR/LbaCas12a,^[^ ^2^, ^17^ ^]^ CRISPR/AsCas12a,^[^ ^2^, ^18^ ^]^ and CRISPR/MbCas12a^[^ ^19^ ^]^) are preferable to one‐pot LAMP‐CRISPR methods. Unlike CRISPR/Cas12b, mesophilic CRISPR/Cas12a requires shorter crRNA (below 50 nt) and efficiently recognizes DNA amplicons, facilitating INAA‐CRISPR's efficiency. Nevertheless, one‐pot RPA‐CRISPR/Cas12a assays remain challenges in performance, multiplexing, and field‐ready lyophilization.

In this study, we report an enhanced one‐pot, helicase‐assisted RPA (hRPA)‐combined, dual‐CRISPR/uAsCas12a (EOD‐CRISPR) assay lyophilized on a 3D‐printed microfluidic disc to enable field‐deployable, high‐performance multiplex CRISPR‐based detection. The speed, sensitivity, and fluorescence signal of EOD‐CRISPR assay are enhanced due to the synergistic effect of bovine serum albumin (BSA), hRPA, and uAsCas12a.^[^ ^20^ ^]^ To achieve multiplex detection, a monolithic, 3D‐printed microfluidic disc is designed to enable simultaneous detection of four targets. On disc chambers, EOD‐CRISPR reagents are stably lyophilized using glass fiber membranes and a protectant comprising 5% trehalose and 1% glycine. Moreover, a field‐deployable detection platform is formed by assembling 3D‐printed DNA extractor, portable fluorescence detector, and mini centrifuge with EOD‐CRISPR‐lyophilized discs. The platform is also validated by detecting four foodborne bacteria, *Bacillus cereus* (*B. cereus*), *Salmonella enterica* (*Salmonella*), *Staphylococcus aureus* (*S. aureus*), and *Escherichia coli* O157:H7 (*E. coli* O157:H7) from five types of synthetic food. Our portable, reliable, multiplex EOD‐CRISPR assay therefore excels in onsite bacteria testing and more.

## 2 Results

### 2.1 Overview of the EOD‐CRISPR Assay

**Figure 1:**
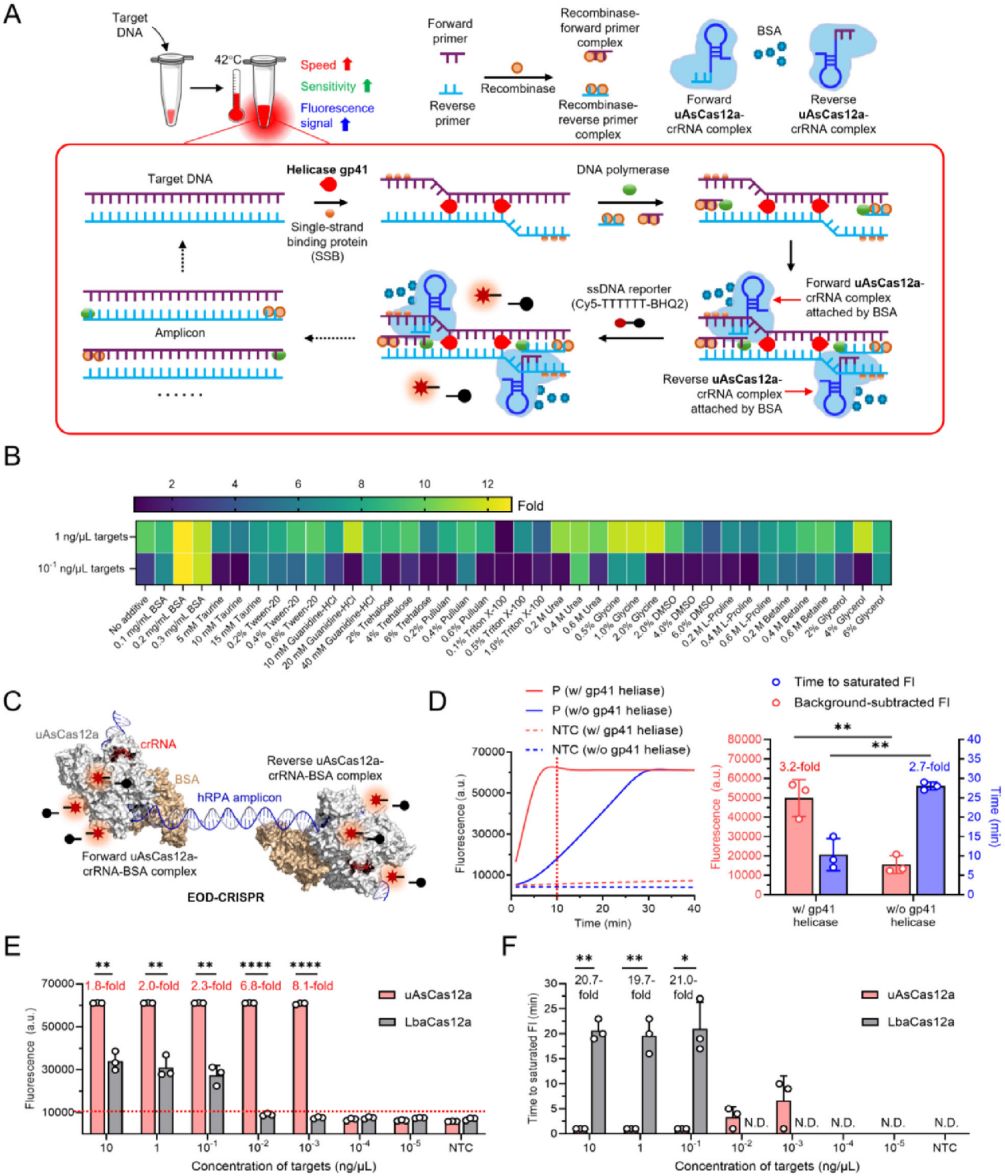
Principle and development of the EOD‐CRISPR assay. A) Schematic illustration. B) Effect of various additives on the assay. Fold, the average fluorescence ratio of three replicated (n = 3) positive reactions (with 1 or 10^−1^ ng µL^−1^ of *S. aureus* genomic DNA) to no‐target controls. C) Molecular simulation of BSA‐interacted *trans*‐cleavage activity of uAsCas12a. hRPA, helicase‐assisted recombinase polymerase amplification. D) Effect of gp41 helicase on the assays. P, the reactions with 5 × 10^−2^ ng µL^−1^ of *S. aureus* genomic DNA. Histograms present background‐subtracted FI at 10 min and the time to saturate FI. FI, fluorescence intensity. E) Comparison of endpoint FI at 10 min and F) the time to saturated FI for the assays with LbaCas12a or uAsCas12a. Various concentrations of *S. aureus* genomic DNA were used. Average fold changes of FI between two groups were calculated. Horizontal dashed red line represents the threshold (10^4^) of FI. Three replicates (n = 3) were set for each test. Error bars represent the standard deviations of replicates. Unpaired two‐tailed t‐test was used to analyse the significant difference between two groups. NTC, no‐target control. *, *p* < 0.05; **, *p* < 0.01; ****, *p* < 0.0001; N.D., not detected.

As shown in **Figure** **1**A, the EOD‐CRISPR assay method combines helicase‐assisted RPA (hRPA) components and dual (forward and reverse) uAsCas12a‐crRNA complexes in one solution. When incubating at 42 °C, the helicase gp41 efficiently unwinds double‐stranded (ds) target DNA and the single‐strand binding protein (SSB) binds to the exposed single‐stranded (ss) regions. Then, the forward and reverse recombinase‐primer complexes anneal to their target sites, initiating primer extension by DNA polymerase. Meanwhile, BSA‐attached forward and reverse uAsCas12a‐crRNA complexes recognize their corresponding binding sites, activating uAsCas12a's *trans*‐cleavage activity. The activated uAsCas12a efficiently cleaves the high‐concentration fluorophore‐labeled ssDNA reporter (i.e., Cy5‐TTTTTT‐BHQ2) in the reaction, producing target‐specific fluorescent signals. Following primer extension, the resulting amplicons function as new target DNAs to mediate similar subsequent processes. In EOD‐CRISPR reactions, hRPA‐based exponential DNA amplification and efficient dual‐CRISPR‐based detection occur simultaneously, thereby enhancing its speed, sensitivity, and fluorescence signal.

To improve fluorescence signals, we first investigated the effect of 13 chemical additives on EOD‐CRISPR reactions via measuring the fold change of fluorescence between positive reactions and no‐target controls (NTCs). As displayed in Figure 1B and Figures S1–S5 (Supporting Information) 0.2 mg mL^−1^ BSA in the tested positive reactions provided the most enhancement (over 12‐fold change) on fluorescence signal. Since BSA is a widely used protein stabilizer,^[^ ^21^ ^]^ it could stabilize uAsCas12a in EOD‐CRISPR reactions, thereby exhibiting strong *trans*‐cleavage activity. To this end, AlphaFold Server was used to model the interaction between BSA and uAsCas12a. As shown in Figure S6A (Supporting Information) 15 polar contacts were obviously observed, implying that the enhancement was associated with the two proteins’ intermolecular interaction (Figure 1C). Certainly, the underlying mechanism will be unveiled by future follow‐up studies.

Next, EOD‐CRISPR assays with and without helicase (i.e., bacteriophage T4 gp41 helicase) were compared. Helicase gp41 is a hexameric helicase that unwinds DNA with 5′ to 3′ polarity to promote DNA replication.^[^ ^22^ ^]^ So, supplementing gp41 could enhance RPA performance through ensuring efficient strand separation. As shown in Figure 1D, gp41‐loaded EOD‐CRISPR assays at 10 min produced significantly higher (3.2‐fold) fluorescence signal than the assays in absence of gp41. Further, the assays without gp41 required obviously longer (2.7‐fold) time to reach saturated fluorescence. Thus, the gp41‐assisted RPA (i.e., hRPA) increases the EOD‐CRISPR's fluorescence signal and speed.

Last, instead of using LbaCas12a, a highly active uAsCas12a was expressed and applied to EOD‐CRISPR assays (Figure S6B,C, Supporting Information). The uAsCas12a is a variant of *Acidaminococcus sp*. Cas12a (AsCas12a) and performs markedly increased efficacy due to two‐point mutations of M537R and F870L.^[^ ^20^ ^]^ As shown in Figure 1E and Figure S7 (Supporting Information), at the point of 10‐min incubation, the sensitivity of EOD‐CRISPR assays with uAsCas12a was 10^−3^ ng µL^−1^ targets, which increased by two orders of magnitude when compared to it (10^−1^ ng µL^−1^) of the assays with LbaCas12a. Also, their fluorescence signals were generally elevated with the fold change from 1.8 to 8.1 (Figure 1E). Moreover, uAsCas12a accelerated the reactions significantly, since the time to saturated fluorescence was much shorter (1‐5 min) (Figure 1F).

Collectively, the EOD‐CRISPR with BSA, hRPA, and uAsCas12a enables highly efficient one‐pot RPA‐dual‐CRISPR assay in terms of speed (less than 5 min for time to saturated fluorescence), sensitivity (increased by two orders of magnitude), and fluorescence signal (up to 8.1‐fold change).

### 2.2 Tube‐Based EOD‐CRISPR Assays for Foodborne Bacteria Detection

According to the World Health Organization, the illnesses by foodborne bacteria cause over 0.4 million people death as well as the huge economic losses each year.^[^ ^23^ ^]^ Timely and accurate detection of foodborne bacteria is of great significance.

**Figure 2:**
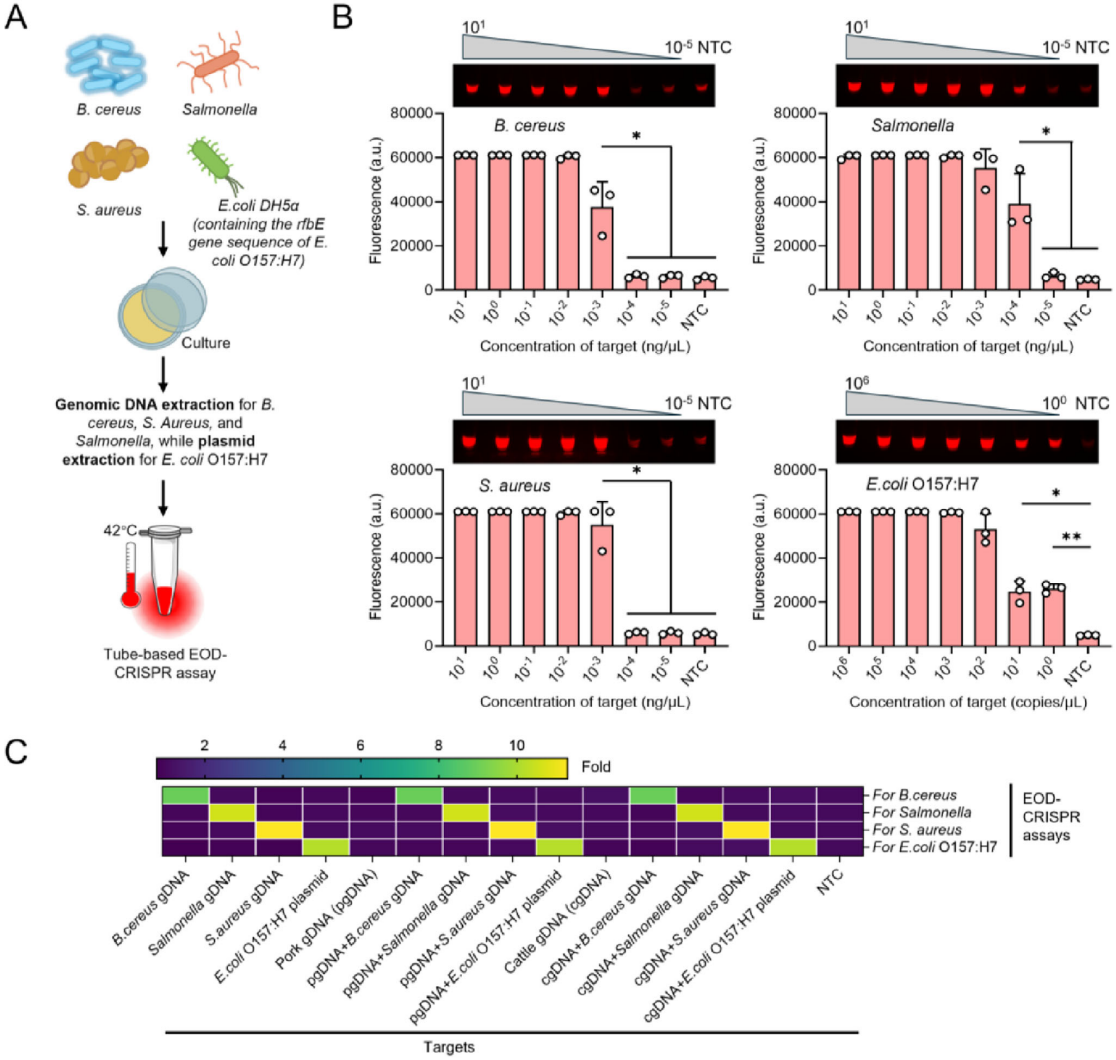
Sensitivity and specificity of tube‐based EOD‐CRISPR assays for foodborne bacteria detection. A) Workflow of the assays. Target DNAs were the genomic DNAs extracted from *B. cereus*, *Salmonella*, and *S. aureus* strains, and the plasmid DNAs (inserted with the *rfb*E gene sequence of *E. coli* O157:H7) extracted from *E. coli* DH5α strain. B) Sensitivity of the assays at 20 min when detecting various concentrations of bacterial genomic DNAs. Three replicates (n = 3) were run for each test. Error bars represent the standard deviations of replicates. Unpaired two‐tailed t‐test was used to analyse the significant difference between two groups. **, *p* < 0.01; *, *p* < 0.05. NTC, no‐target control. C) Specificity of the assays when detecting pure bacterial genomic DNA and their mixtures with gDNA from pork or cattle meats. gDNA, genomic DNA. Fold, the average fluorescence ratio of three replicated (n = 3) positive reactions to no‐target controls.

Given the high performance of EOD‐CRISPR reaction, we developed its tube‐based assays to detect four high‐risk foodborne bacteria, *B. cereus*, *Salmonella*, *S. aureus*, and *E. coli* O157:H7, by targeting their specific genes of *mur*B, *inv*A, *nuc*, *rfb*E, respectively. As shown in **Figure** **2**A, the sensitivity of tube‐based EOD‐CRISPR assay was investigated by detecting various concentrations of the genomic DNAs from *B. cereus*, *Salmonella*, and *S. aureus* strains, as well as the plasmid DNAs from *E. coli* DH5α strain which contains the *rfb*E gene sequence of *E. coli* O157:H7. When incubated at 42 °C for 20 min, tube‐based EOD‐CRISPR assays could stably detect down to 10^−3^, 10^−4^, 10^−3^ ng µL^−1^, and 1 copy µL^−1^ of *B. cereus*, *Salmonella*, *S. aureus*, and *E. coli* O157:H7 DNA, respectively (Figure 2B). Also, the fluorescence‐based visual detections showed the same detection sensitivity. In addition, the sensitivity was further evaluated using various concentrations (colony‐forming units per milliliter; CFU/mL) of genomic DNA extracted from bacterial cultures. As illustrated in Figure S8 (Supporting Information) the EOD‐CRISPR assay demonstrated stable detection capability for as low as 10^2^, 10^2^, 10^1^, and 10^1^ CFU mL^−1^ of *B. cereus, Salmonella, S. aureus*, and *E. coli* O157:H7 strains, respectively.

The specificity of tube‐based EOD‐CRISPR assay was estimated through testing pure bacterial genomic DNAs and their mixtures with the genomic DNAs of pork and cattle meats. As shown in Figure 2C, tube‐based assays performed high specificity to corresponding targets, even in the presence of genomic DNAs from pork and cattle meats. The high specificity is likely contributed to by high intrinsic specificity (i.e., low off‐target effect) of CRISPR/uAsCas12a system.^[^ ^20^ ^]^

Together, tube‐based EOD‐CRISPR assays are rapid (within 20 min), highly specific (without target‐cross reactivity), and highly sensitive (down to 10^−4^ ng µL^−1^, 1 copy µL^−1^ or 10^1^ CFU mL^−1^ targets) for foodborne bacteria detection.

### 2.3 Design and Treatment of 3D‐Printed Microfluidic Disc

Although tube‐based EOD‐CRISPR assays already possess high performance, the detection multiplexing is limited to one single target. Thus, we designed a monolithic, 3D‐printed microfluidic disc for EOD‐CRISPR assays. The disc was one‐step fabricated using a stereolithography (SLA) 3D printer, avoiding aligning and binding multiple layers in fabrication of polymethyl methacrylate (PMMA)‐based lab‐on‐disc.^[^ ^24^ ^]^

**Figure 3:**
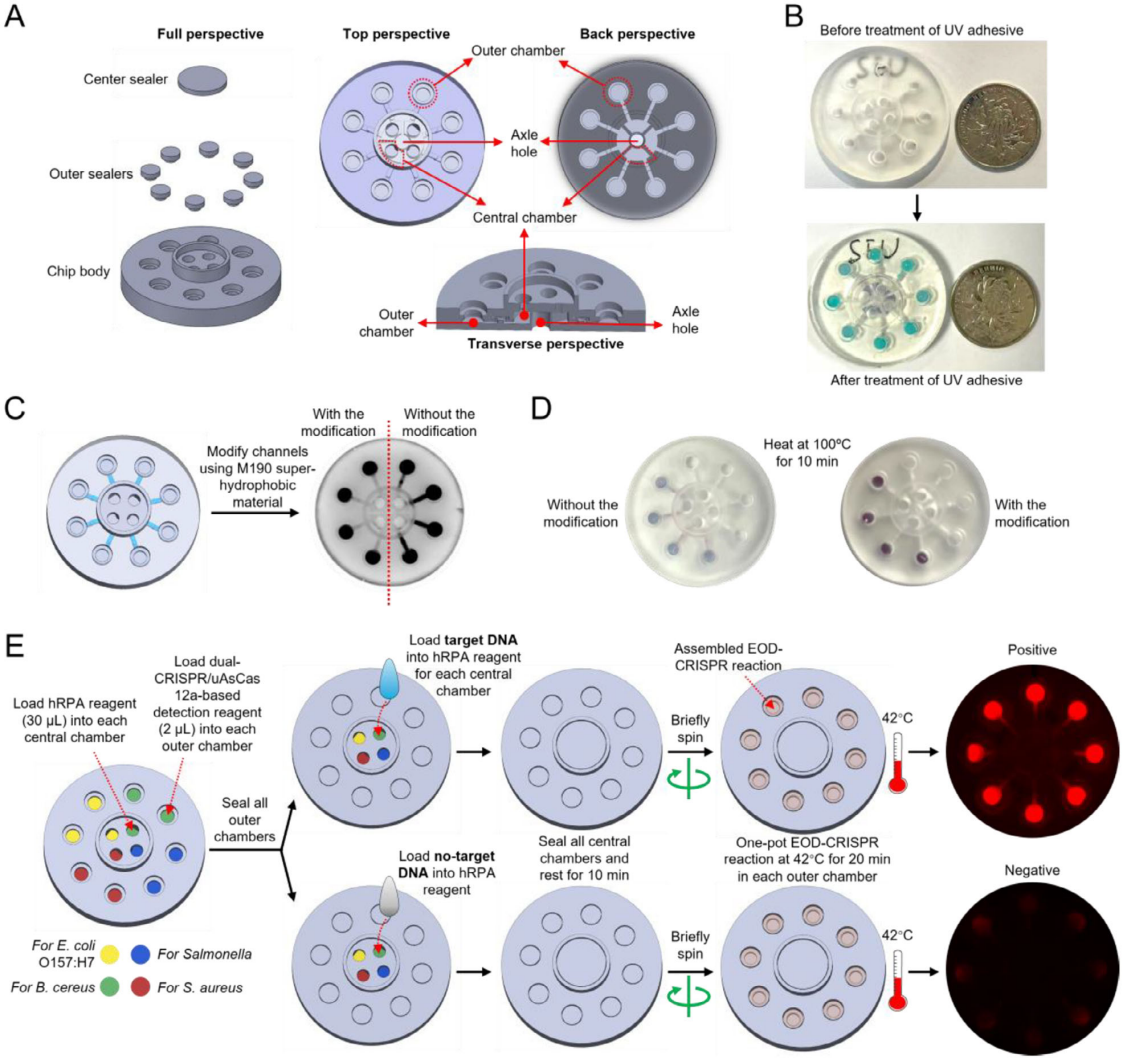
Multiplex EOD‐CRISPR assay on a 3D‐printed disc. A) Disc design. B) Treatment for disc transparency using a ultraviolet (UV) adhesive. C) Hydrophobic modification of channels. D) Comparison of liquid distribution on chips with and without hydrophobic modification after heating at 100 °C for 10 min. E) Workflow of multiplex EOD‐CRISPR assay for foodborne bacteria detection using the disc. Each outer chamber contained 2 µL of dual‐CRISPR/uAsCas12a‐based detection reagent and each central chamber was filled with 30 µL of hRPA reagent. Target DNA, the mixed genomic DNA of *B. cereus*, *Salmonella*, *S. aureus*, and *E. coli* O157:H7. No‐target DNA, the pork's genomic DNA. hRPA, helicase‐assisted recombinase polymerase amplification.

As shown in **Figure** **3**A, the chip body contained eight outer chambers and four central chambers. Each central chamber connected two outer chambers. Each outer chamber had one sealer (outer sealer), but all the central chambers shared with one sealer (central sealer). Also, an axle hole was designed to attach a mini centrifuge for sample loading. Consequently, this disc has a potential in applying for onsite detection. However, PMMA‐based discs depend on benchtop centrifuges, unsuitable for field deployment.^[^ ^25^ ^]^ The sizes of chambers, channels, and the whole chip were all presented in Figure S9 (Supporting Information). Theoretically, the volumes of central chamber, outer chamber, and their collected channel should be 36.738, 10.048, and 4.368 µL (Figure S9C, Supporting Information). If loading 2 µL CRISPR reagent and 30 µL hRPA reagent into each outer and central chambers, respectively, the final EOD‐CRISPR solution post centrifuge should fill in each outer chamber and its collected channel completely, providing a constant reaction volume (≈ 14.416 µL).

To improve the disc's transparency for fluorescence detection, an ultraviolet (UV) adhesive was used to coat the surface (Figure 3B). Previously, heat‐resistant acrylic spray was utilized to increase transparency, while requiring a long‐time curing period after treatment.^[^ ^26^ ^]^ Comparatively, the UV adhesive is fast and cost‐efficient for transparency improvement. In addition to transparency, the disc's channels were modified using a superhydrophobic material to block the infiltration of liquid from outer chamber into central chamber, even heating at 100 °C for 10 min (Figure 3C,D).

Subsequently, we applied the disc to the multiplex detection of foodborne bacteria. As shown in Figure 3E, one central chamber and the linked two outer chambers were filled with EOD‐CRISPR reagents specific to corresponding target genes of *B. cereus*, *Salmonella*, *S. aureus*, or *E. coli* O157:H7. Each outer chamber was filled with 2 µL of dual‐CRISPR/uAsCas12a‐based detection reagent, while 30 µL of hRPA reagent for each central chamber. Then, all outer chambers were sealed using UV adhesive. As to on‐disc detection, the genomic DNA mixture of all the four bacteria was loaded into each central chamber prior to sealing the chambers and resting for 10 min. After brief spinning the disc, the final EOD‐CRISPR reactions were assembled in each outer chamber, then subject to incubation at 42 °C for 20 min. Based on the fluorescence imaging, the positive disc exhibited strong red fluorescence, whereas the negative disc targeting pork's genomic DNA showed no fluorescence. Given this, the 3D‐printed disc after treatment is feasible to simultaneously detect four foodborne bacteria, enlarging detection multiplexing.

### 2.4 Lyophilization Process of EOD‐CRISPR Reagents

Toward point‐of‐need detection, transporting and distributing the disc with EOD‐CRISPR reagents to the areas with limited infrastructure is critical.^[^ ^27^ ^]^ Therefore, the lyophilization process is indispensable to achieve high stability and long shelf life of the reagents in logistics for transport and storage. To meet this need, the lyophilization of EOD‐CRISPR reagents were developed and evaluated in this study.

**Figure 4:**
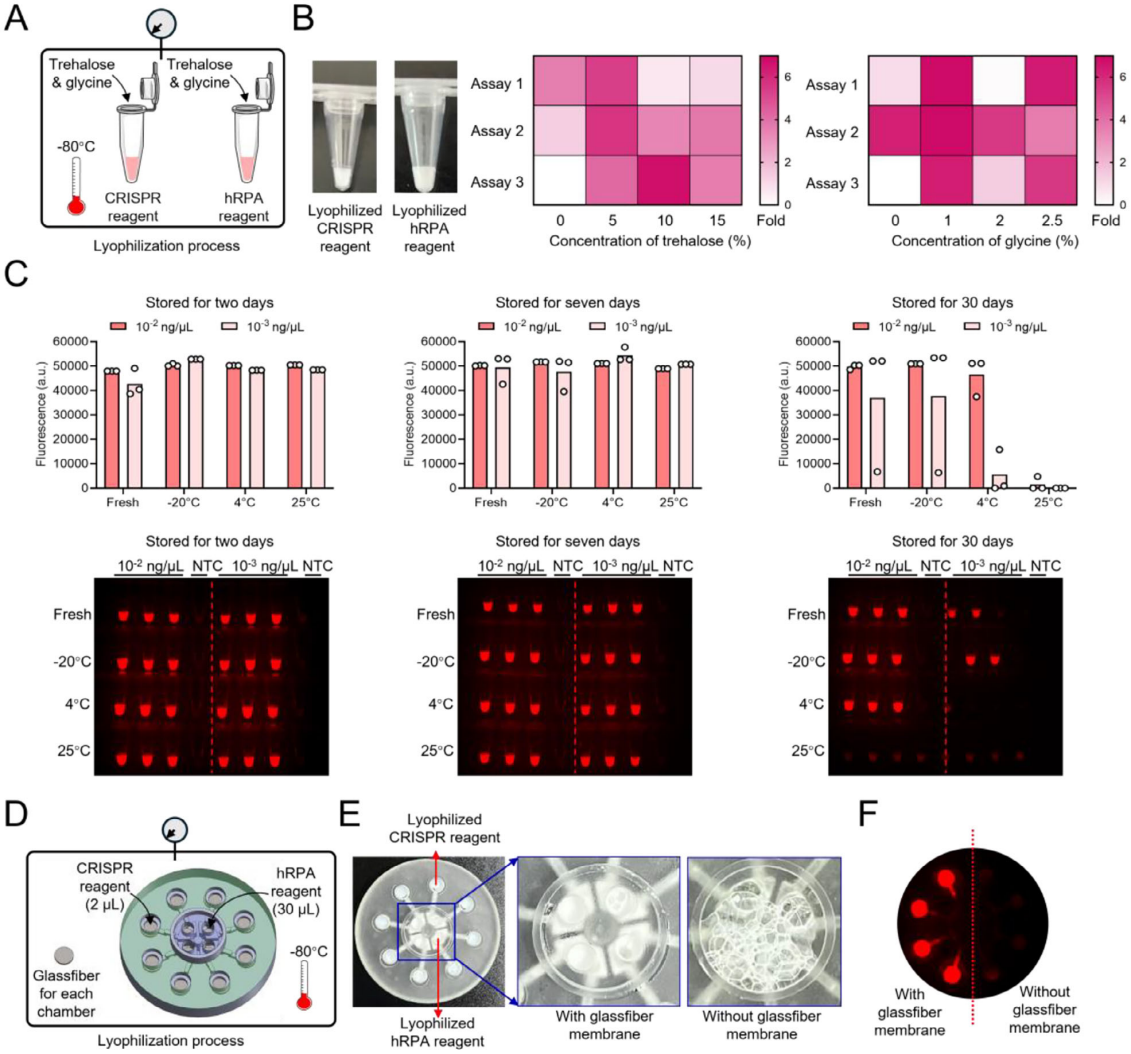
Lyophilization of EOD‐CRISPR reagents in tubes and on discs. A) Lyophilization of EOD‐CRISPR reagents in tubes. The CRISPR and hRPA reagents of EOD‐CRISPR should be lyophilized separately. CRISPR reagent, the dual‐CRISPR/uAsCas12a‐based detection reagent. hRPA, helicase‐assisted recombinase polymerase amplification. B) Optimization of trehalose and glycine for lyophilization in tubes. The fold changes of fluorescence between positive reactions (with 10^−3^ ng µL^−1^ of *S. aureus* genomic DNA) and no‐target controls were used. C) Stability evaluation of lyophilized EOD‐CRISPR assays at various storage temperatures and time. EOD‐CRISPR reactions with 10^−2^ and 10^−3^ ng µL^−1^ of *S. aureus* genomic DNA ran for 20 min. Fresh, the newly prepared reaction. NTC, no‐target control. Three replicates (n = 3) were run for each test. D) Lyophilization of EOD‐CRISPR reagents on disc. E) Enlarged illustration of the central chambers during the lyophilization with or without glassfiber membrane. F) Disc imaging of lyophilized EOD‐CRISPR assays with and without glassfiber membrane when testing the mixed genomic DNA targets of *B. cereus*, *Salmonella*, *S. aureus*, and *E. coli* O157:H7.

First, we investigated whether the mixture of trehalose (a nonreducing disaccharide) and glycine (a bulking agent) was helpful for stable EOD‐CRISPR lyophilization in tubes (**Figure** **4**A), as it was included as a protectant in lyophilized RPA‐CRISPR reactions.^[^ ^28^ ^]^ As shown in Figure 4B, the optimal mixture was composed of 5% trehalose and 1% glycine to enable robust EOD‐CRISPR assays. However, the CRISPR and hRPA reagents of EOD‐CRISPR should be lyophilized separately to maintain their own high efficiency.

Next, the stability of lyophilized EOD‐CRISPR assay was investigated after up to 30‐day storing at various temperatures. As indicated in Figure 4C, lyophilized EOD‐CRISPR was able to visually detect 10^−2^ ng µL^−1^ target DNA, even stored at 4 °C for 30 days. Upon detecting 10^−3^ ng µL^−1^ target DNA, lyophilization failed to extend EOD‐CRISPR's shelf life to 30 days at both 4 and 25 °C. Interestingly, our lyophilized EOD‐CRISPR kept stable to detect 10^−3^ ng µL^−1^ target at 25 °C for seven days, possessing non‐deteriorated performance as that at ‐20 °C. Thus, cold chain transportation is potentially unnecessary to deploy EOD‐CRISPR reagents to the places within 7‐day shipment distance.

Besides, we explored the lyophilization of EOD‐CRISPR reagents on the disc (Figure 4D). Unfortunately, when directly loading the reagents into disc chambers, millimeter‐level depth and width hardly supported stale lyophilization due to the generation of bubbles (Figure 4E). To address this issue, we employed glassfiber membranes as substates to adsorb EOD‐CRISPR reagents. As shown in Figure 4E, the adsorbed EOD‐CRISPR reagents in disc chambers were successfully lyophilized without producing any bubbles. Also, on‐disc fluorescence detection was realized using the lyophilized reagents, indicating that glassfiber membranes didn't influence EOD‐CRISPR reactions (Figure 4F).

### 2.5 On‐Disc Lyophilized EOD‐CRISPR Assays for Foodborne Bacteria Detection

**Figure 5:**
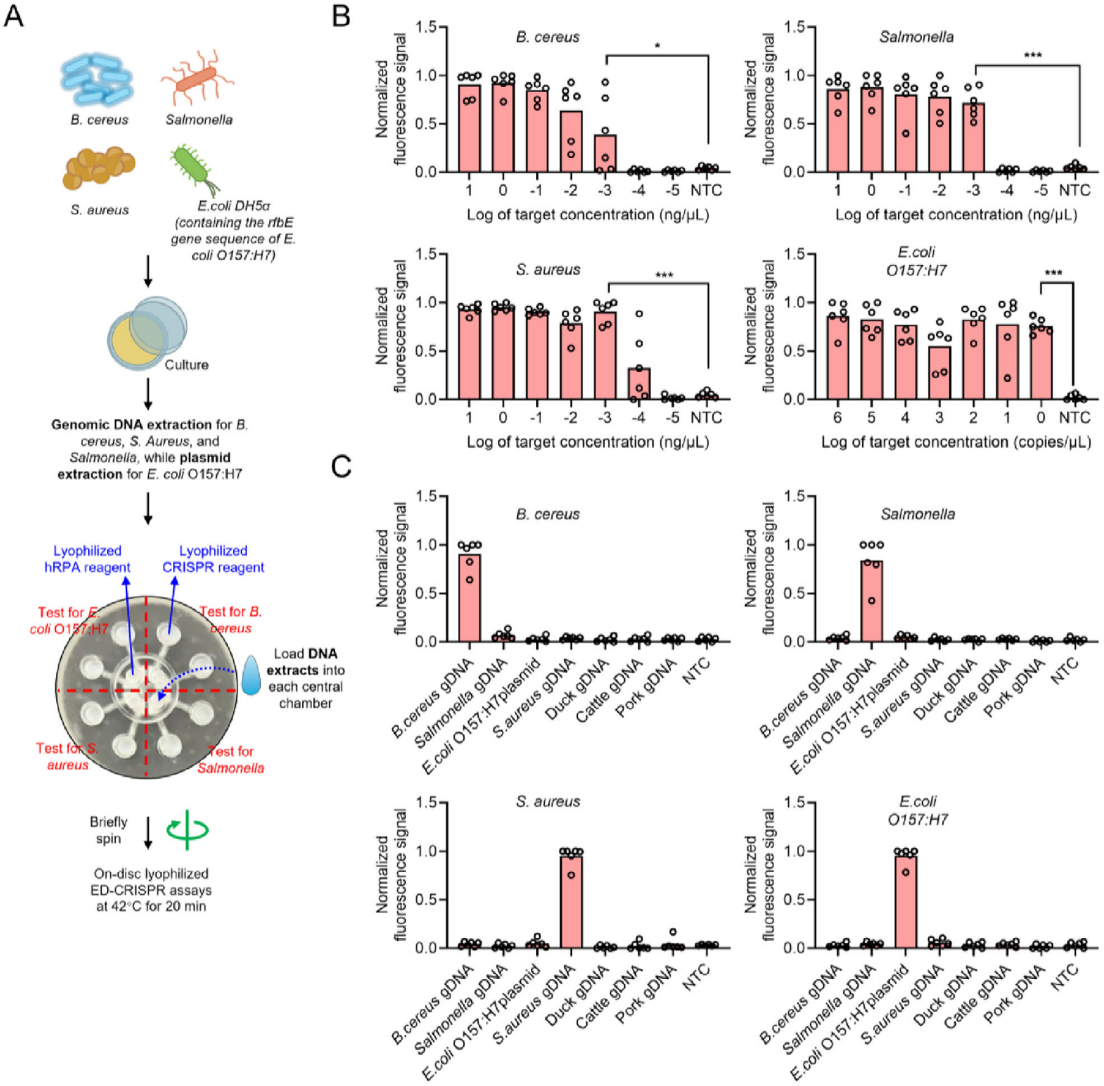
On‐disc multiplex bacteria detection by lyophilized EOD‐CRISPR assays. A) Workflow for the assays. Outer chambers contained the lyophilized CRISPR reagents specific to corresponding bacteria and central chambers B) Sensitivity assays by detecting various concentrations of targets. C) Specificity assays by detecting various bacterial and meat genomic DNA (gDNA). All the tests were incubated at 42 °C for 20 min. Six replicates (n = 6) were run for each test. Error bars represent the standard deviations of the replicates. Unpaired two‐tailed t‐test was used to analyse the significant difference between two groups. gDNA, genomic DNA. hRPA, helicase‐assisted recombinase polymerase amplification. ***, *p* < 0.001; *, *p* < 0.05.

The sensitivity and specificity of lyophilized EOD‐CRISPR assays on discs were estimated through detecting genomic or plasmid DNAs extracted from the culture cells of foodborne bacteria. For each disc, lyophilized EOD‐CRISPR reagents were able to identify the four bacteria of *B. cereus*, *S. aureus*, *Salmonella*, and *E. coli* O157:H7 simultaneously (**Figure** **5**A).

As shown in Figure 5B and Figure S10 (Supporting Information) the results of disc imaging post reaction demonstrated that our on‐disc lyophilized EOD‐CRISPR assays were able to stably detect 10^−3^ ng µL^−1^ genomic DNA extracted from *B. cereus*, *S. aureus*, and *Salmonella*, as well as 10° copy µL^−1^ *E. coli* O157:H7 DNA. Except for *S. aureus*, the sensitivities of our assays were comparable (for *B. cereus* and *Salmonella*) to or better (for *E. coli* O157:H7) than those of PCR assays (Figure S11, Supporting Information). As to the detection of 10^−4^ ng µL^−1^ genomic DNA of *S. aureus*, the average Cq value by PCR was over 30, indicating weak positive targets which our on‐disc assays also occasionally detected (Figure 5B; Figure S10, Supporting Information). Thus, on‐disc lyophilized EOD‐CRISPR assays achieved a comparable sensitivity to PCR assays. Regarding specificity, the on‐disc detection also demonstrated high specificity to the targets. As shown in Figure 5C and Figure S12 (Supporting Information) the phenomenon of cross reactivity was not observed for each target. Together, on‐disc lyophilized EOD‐CRISPR assays possess the same performance as tube‐based assays, in addition to improving detection multiplexing.

### 2.6 Onsite Detection Platform for Foodborne Bacteria Detection

**Figure 6:**
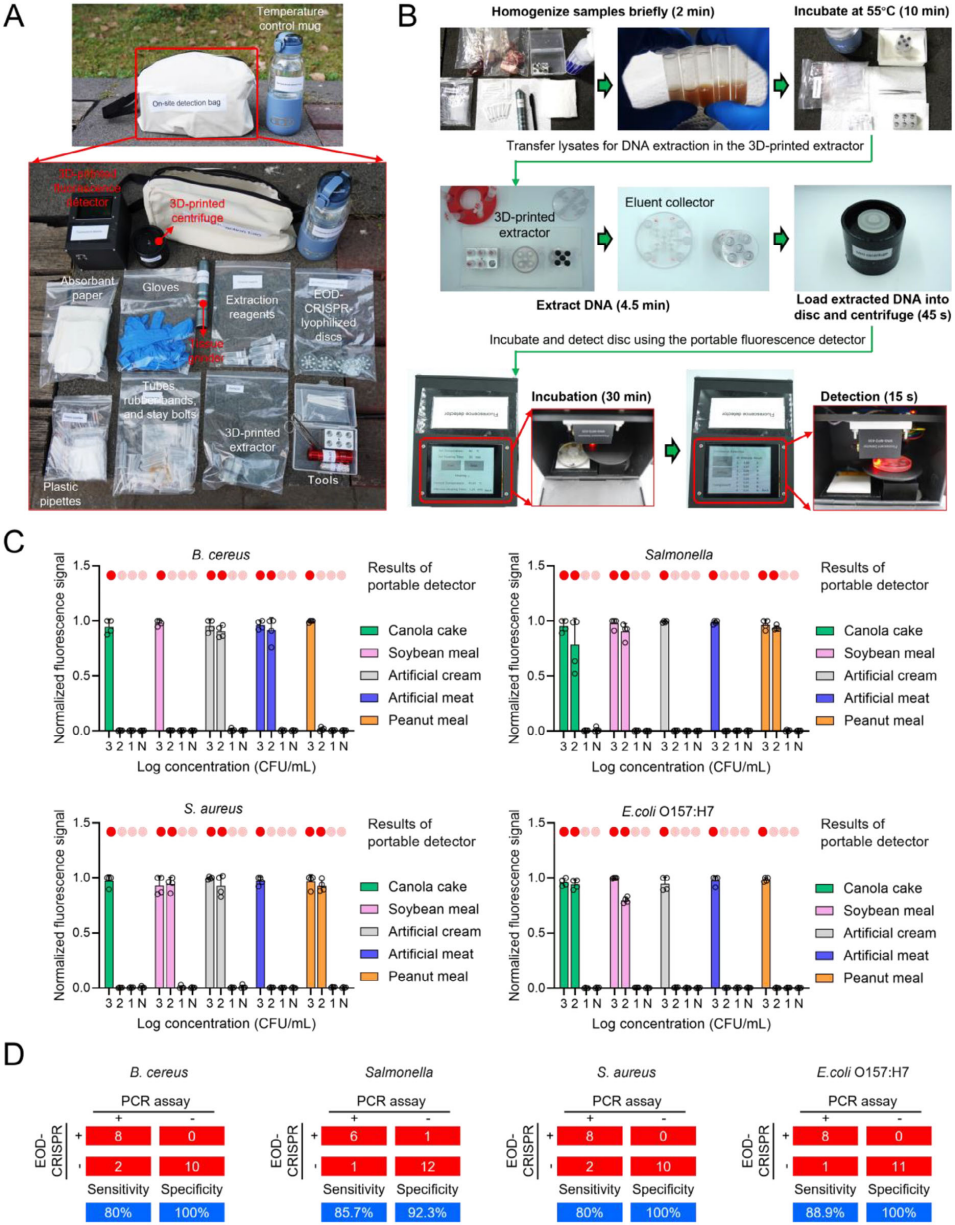
Onsite bacteria detection platform and its performance. A) Photos of the onsite detection bag and the list of materials. B) Workflow of the detection platform (taking meat samples as an example). C) Sample testing using five kinds of synthetic food mixed with various amounts of *B. cereus*, *S. aureus*, *Salmonella*, and *E. coli* O157:H7 cultures. For results from the portable fluorescence detector, red and light red dots presented positive and negative, respectively. Four replicates (n = 4) were run for each target. Error bars represent the standard deviations of the replicates. D) Confusion matrixes describing the performances of EOD‐CRISPR and PCR assays (as the standard) for corresponding bacteria testing. +, positive; ‐, negative.

To meet the requirement of point‐of‐need detection, the EOD‐CRISPR reagents‐lyophilized discs were further coupled with 3D‐printed extractor, 3D‐printed fluorescence detector, and 3D‐printed mini centrifuge to develop an onsite detection bag for foodborne bacteria detection (**Figure** **6**A). The bag also contained the required materials for food matrixes’ homogenization, DNA extraction, and chip operation. All the materials were packed using sterilized plastic bags. Since efficient DNA extraction from food matrixes (e.g., meat) demanded the treatment of proteinase K at 55 °C,^[^ ^29^ ^]^ a temperature control mug with the constant temperature of 55 °C was also utilized. Thus, our onsite detection platform comprised the detection bag and the temperature control mug (Figure 6A), readily handling bacterial contamination events at point of need.

The 3D‐printed extractor comprised binding array, mini barrel, mini pump, residue collector, and eluent collector (Figure S13A, Supporting Information). After filling the binding array with circular glassfiber membranes (D = 7 mm and THK = 2 mm), the assembled extractor could extract and purify DNA from five samples simultaneously. The average extraction rate by the extractor was tested to be ≈20% when extracting high (10 ng µL^−1^) or low (10^−1^ ng µL^−1^) concentration of DNA targets (Figure S13B, Supporting Information). The DNA recovery rate of our mini extractor was similar as it reported previously.^[^ ^30^ ^]^ In future follow‐up studies, we will customize the extraction reagents to further improve extraction rate.

The inner structure of the 3D‐printed portable fluorescence detector housing was shown in Figure S14A (Supporting Information). The detector (size: 140 mm × 110 mm × 105 mm) with power consumption of under 10 W combined heating, fluorescence detection, display, control, and power supply modules in the 3D‐printed housing. The heating module employed a 40 mm × 40 mm alumina ceramic heater, while the fluorescence detection module used a detector with 635 nm excitation and 685 nm detection wavelengths. A 3.2‐inch display enabled real‐time data visualization, and the control module, featuring an STM32F103 microcontroller, NTC thermistor, and PID algorithm, ensured precise thermal regulation between 20–80 °C with ± 0.8 °C accuracy. The system's rechargeable lithium‐ion battery powered all modules, with the microcontroller coordinating a stepping motor and 12‐bit ADC for accurate sample array scanning.

The 3D‐printed mini centrifuge was sized with the height of 64 mm and the external diameter of 75 mm (Figure S14B, Supporting Information), composed of two rechargeable lithium‐ion batteries and a mini electric motor (export 10000 rpm). The spin axis of the motor was tightly inserted into the axle hole of the disc. The mini centrifuge was used to rapidly load liquids into the disc's chambers at point of need.

As shown in Figure 6B, when taking meat samples as an example, homogenization was the first step to disrupt cells to release bacteria efficiently. In our onsite detection bag, a handhold tissue grinder was utilized to homogenize samples within 2 min. Subsequently, the samples mixed with lysis buffer and proteinase K were incubated at 55 °C for 10 min using the temperature control mug. Then, the digested samples were subject to DNA extraction using the 3D‐printed extractor. The detailed procedures of sample treatment and DNA extraction were shown in Figure S15 (Supporting Information). Post eluent collecting, 30 µL of DNA extract was loaded into each disc's central chamber, followed by brief centrifuging. Prior to incubation and detection in the detector, the disc's central chambers were sealed by center sealer using the UV adhesive under the exposure of UV flashlight. Ultimately, the detection results were shown on the screen. Using our onsite detection platform, the whole time from sample collection to data acquisition was in less than 48 min.

To validate the applicability of our onsite detection platform, we tested five types of synthetic food including canola cake, soybean meal, artificial cream, artificial meat, and peanut meal. For each type, 10^3^, 10^2^, and 10^1^ CFU mL^−1^ of cultured *B. cereus*, *S. aureus*, *Salmonella*, or *E. coli* O157:H7 cells were added in a final concentration to mimic real food‐contaminated samples. As shown in Figure 6C and Figures S16–S19 (Supporting Information) the assay results of disc imaging using a benchtop imaging system were consistent with those of the portable fluorescence detector. Compared with the standard PCR methods which rely on table centrifuges and spin columns for DNA extraction (Tables S5–S8, Supporting Information), our onsite detection platform with rapid on‐disc DNA extraction performed 80%‐88.9% sensitivity and 92.3%‐100% specificity (Figure 6D). Since the 3D‐printed extractor's DNA extraction efficiency was around 20% (Figure S13, Supporting Information), the sensitivity of the onsite detection platform was lower than the PCR assays which require benchtop centrifuge‐based DNA extraction. In future follow‐up studies, we'll try to increase the extractor's extraction efficiency through customizing or optimizing the extraction reagents. Anyway, from the perspective of onsite detection, the platform possesses comparable accuracy to the standard PCR assay for food sample testing.

In addition to synthetic food, pork and chicken meats were spiked with 10^3^ CFU mL^−1^ of cultured *B. cereus*, *S. aureus*, *Salmonella*, or *E. coli* O157:H7 cells to resemble foodborne illness outbreaks. As shown in Figure S20 (Supporting Information) our onsite detection platform could identify the four bacteria from raw meats, readily adapted to the prevention and control of foodborne illness outbreaks.

## 3 Discussion

In the present study, we report an enhanced one‐pot hRPA‐combined dual‐CRISPR/uAsCas12a assay, namely EOD‐CRISPR. To achieve multiplex detection, EOD‐CRISPR reagents are successfully lyophilized on a 3D‐printed microfluidic disc. Toward point‐of‐need applications, we apply EOD‐CRISPR‐lyophilized discs to build a bacteria detection platform which comprises an onsite detection bag and a temperature control mug. Upon testing bacteria‐spiked synthetic food samples, the onsite detection platform achieves comparable detection accuracy to standard PCR assays. In addition, the “sample‐to‐answer” time of the onsite detection platform is less than 48 min, providing a filed‐deployable, rapid, multiplex one‐pot CRISPR‐based bacteria detection toolbox.

Presently, developing efficient one‐pot RPA‐CRISPR/Cas12a reaction remains challenges, due to the intrinsic incompatibility of RPA and CRISPR/Cas12a components. For example, the *cis*‐cleavage activity of Cas12a can digest the RPA's amplification targets, thereby lowering detection sensitivity of one‐pot assays. Thus, balancing Cas12a's *cis*‐cleavage and *trans*‐cleavage activities in the assays is of importance. To address this issue, a variety of strategies such as photocontrolled crRNA activation,^[^ ^31^ ^]^ phase‐separation propensity of recombinase,^[^ ^32^ ^]^ phase separation by glycerol^[^ ^33^ ^]^ or sucrose viscosity,^[^ ^34^ ^]^ and suboptimal protospacer adjacent motifs of Cas12a^[^ ^35^ ^]^ have been applied. However, these approaches entail complicated optimization of chemical modification, recombinase, and liquid viscosity to guarantee high efficiency. In contrast, introducing dual CRISPR/Cas12a systems into reaction system is a straightforward strategy,^[^ ^11^ ^]^ whereas the detection performance varies with dosage of components. In this study, rather than tuning components, we systemically investigate the effect of 13 additives on the basic hRPA‐dual‐CRISPR/Cas12a reaction and find that 0.2 mg mL^−1^ BSA increases the fluorescence fold change by over 12 times (Figure 1B). Along with BSA, gp41 helicase and uAsCas12a further facilitate the detection performance (Figure 1D,E). In comparison with previous one‐pot RPA‐CRISPR/Cas12a assays (Table S9, Supporting Information), our EOD‐CRISPR reaction has prominent advantages in efficiency of sensing, assay time, and sensitivity.

Besides performance, multiplexing and field‐ready lyophilization are two vital challenges that limit the practical application of one‐pot RPA‐CRISPR/Cas12a assays. To this end, lab‐on‐disc platforms are preferably developed through integrating centrifugal microfluidic discs with one‐pot CRISPR‐based detection.^[^ ^36^ ^]^ However, fabricating centrifugal discs currently requires complicated aligning and binding processes of multiple layers which are made via soft lithography, micromachining, and hot embossing and injection molding.^[^ ^24^, ^37^ ^]^ In this study, we use a simple SLA‐based 3D printing technology to rapidly manufacture complex microfluidic discs for multiplex EOD‐CRISPR assays. After treated using UV adhesive and superhydrophobic material, the discs possess improved optical transparency and the infiltration of liquid between chambers is blocked (Figure 3). To achieve stable lyophilization, tube‐based EOD‐CRISPR reactions are supplemented with a protectant of 5% trehalose and 1% glycine (Figure 4A). However, due to low depth (0.8 mm) and low diameter (4 mm) of chambers, bubbles are obviously caused during lyophilization of EOD‐CRISPR reagents on discs (Figure 4C). To solve this obstacle, glassfiber membranes are utilized as substrates to absorb or immobilize the reagents, thereby enabling successful lyophilization on disc chambers. The EOD‐CRISPR‐lyophilized disc does not only detect four targets simultaneously, but also enables easy transportation and distribution into resource‐limited settings for point‐of‐need detection. To our knowledge, it is the first report of multiplex one‐pot dual‐CRISPR/Cas12a assay lyophilized on a 3D‐printed microfluidic disc successfully. Presumably, in addition to EOD‐CRISPR, the 3D‐printed disc we developed can be further adapted to enhance the multiplexing capability of other emerging CRISPR‐based bacterial assays, such as amplification‐free CRISPR‐cascade reaction^[^ ^38^ ^]^ and paper‐based, CRISPR‐responsive visual or label‐free bacterial detection.^[^ ^39^ ^]^

Given the high performance, tube‐ and disc‐based EOD‐CRISPR assays are then adapted to monitoring food safety caused by four common foodborne bacteria, *B. cereus*, *S. aureus*, *Salmonella*, or *E. coli* O157:H7. As expected, our EOD‐CRISPR reactions perform high sensitivity and specificity to detect genomic DNA targets of corresponding bacteria (Figures 2 and 5). Furthermore, an onsite detection platform based on EOD‐CRISPR‐lyophilized discs is validated by testing bacteria‐contaminated synthetic food samples (Figure 6C,D), possessing 80%‐88.9% sensitivity and 92.3%‐100% specificity when compared with standard PCR assay. The relatively low detection sensitivity is likely associated with the low rate (≈ 20%) of the 3D‐printed extractor's DNA extraction, which will be increased through customizing or optimizing the extraction reagents in our future follow‐up studies.

In dealing with the outbreak of foodborne bacterial contamination event, the time to identify types of bacteria greatly determines the intervention and treatment of infectious diseases. As shown in Figure S21 (Supporting Information) we propose a new emergency coping strategy to handle the outbreak of food safety events. With our rapid onsite detection platform, the time from sample collection to data acquisition can be narrowed down to 48 min, enabling quick response to food safety threat. Since there's no linear relationship between target concentration and fluorescence intensity, the current version of EOD‐CRISPR assay is not adapted to the quantitative detection of non‐zero‐tolerance bacteria such as *B. cereus*. However, the high fold change of fluorescence between positive and negative EOD‐CRISPR reactions allows it to be suitable for microwell‐ or droplet‐based digital CRISPR detection,^[^ ^18^, ^40^ ^]^ thereby providing quantitative results. Certainly, in addition to food safety screening, our multiplex EOD‐CRISPR‐based platform holds great potential for other applications such as emerging pathogen detection, clinical diagnostics, animal epidemic prevention, and more.

## 4 Experimental Section

### Materials

All the used plasmids and primers were synthesized by Sangon Biotech (China). The crRNAs for CRISPR/Cas12a systems were synthesized by Genery Biotech (China). The dual‐labeled oligonucleotide probes were synthesized by Accurate Biology (China). The NEBuffer r2.1 was purchased from New England Biolabs (NEB, USA). The BSA was purchased from Beyotime (China). The multienzyme isothermal rapid amplification (hRPA) kit was purchased from Amp‐Future Biotech (China). The DNA extraction kit was purchased from QIAGEN Biotech (USA). All food‐borne microorganisms (*B. cereus* BNCC134491, *Salmonella enterica subsp. enterica* BNCC138602, *S. aureus subsp. aureus* BNCC353548, and *E. coli* O157:H7 BNCC186579) were purchased from BeNa Culture Collection (China). The hydrophobic agent (M190) was purchased from DASU NANO(China). The glass fiber membrane was purchased from Chemxyz (China). The UV shadow‐free adhesive was purchased from Henkel (China). The AceQ Universal SYBR PCR Master Mix were purchased from Vazyme Biotech (China).

### Design of Primers, crRNAs, and Probes

Nucleotide sequence data for strains from GenBank (NCBI, USA) were aligned to identify conserved regions. The conserved regions are listed in Tables S1 (Supporting Information). The primers were designed based on the *nuc* gene of *S. aureus*, the *rfb*E gene of *E. coli* O157:H7, the *inv*A gene of *Salmonella*, the *mur*B gene of *S. aureus*. The used hRPA primer, crRNAs, and probes in the study were all designed using the online Primer Explorer tool (https://primerexplorer.jp/e/) with empirical modifications. All the sequence information is listed in Tables S2–S4 (Supporting Information).

### Bacterial Culture Preparation and DNA Extraction

The bacterial liquid stored in glycerol (2.5 µL) were cultured into 5 mL of a liquid medium and incubated at 37 °C for 10 h under 250 rpm mixing. Bacterial genomic DNA was then extracted using the DNeasy Blood & Tissue kit (QIAGEN) according to the manufacturer's instruction. The concentration was quantified using a spectrophotometer. Finally, the DNA extracts were stored at ‐20 °C until use.

### UAsCas12a Protein Expression and Purification

The uAsCas12 overexpression vectors^[^ ^20^ ^]^ with a His‐Tag were transformed into chemically competent *E. coli* BL21(DE3) and incubated overnight at 37 °C using the LB agar plates containing 50 µg mL^−1^ Kanamycin. Then, single colonies were picked out to inoculate 50 mL LB starter cultures (50 µg mL^−1^ Kanamycin) and incubated shaking vigorously overnight at 37 °C. Next day, 40 mL of the previous starter cultures were transferred to inoculate 1.5 L TB media (50 µg mL^−1^ Kanamycin) and grown at 37 °C to an OD_600_ of ≈ 0.6, followed by cooling down on ice. The gene expression was subsequently induced with isopropyl β‐d‐1‐thiogalactopyranoside (IPTG, 1 mm in final concentration) when incubated at 18 °C for 16 h. After the cells were collected and centrifuged, they were resuspended in lysis buffer (20 mm Tris‐HCl, 1mm TECP, 1 m NaCl, 20 mm imidazole, and 10% glycerol, pH 7.5), then sonicated until the liquid was clear. Then, the soluble fraction was purified using HyPur T Ni‐NTA 6FF (BBI LIFE SCIENCES CORPORATION, China) and then eluted with elution buffer containing 1× PBS, pH 7.5 (with 50, 100, 200, 400, 600, and 800 mm imidazole). The resulting protein was verified using SDS‐PAGE/Coomassie staining. The protein concentration was quantified using a BCA Protein Assay Kit (Beyotime, China). The purified protein was finally stored at ‐80 °C until further use.

### The EOD‐CRISPR Assay

The EOD‐CRISPR reaction was prepared separately as Component A and B. Component A was the dual‐uAsCas12a‐crRNA mix containing 0.2× NEB Buffer r2.1, 0.5 µm each of crRNAs, 0.2 mg mL^−1^ BSA, 2 µm ssDNA probe, and 0.64 µM uAsCas12a. Component B was the hRPA mix containing 1× Rehydrated hRPA mix and 0.5 µm each of hRPA primers. The concentration for each agent was calculated based on the finally assembled reaction (10 µL). In a typical EOD‐CRISPR assay, 1 µL of the target solution and 0.5 µL Buffer B (from hRPA kit) were mixed with 6.3 µL of Component B and left at ambient temperature for 5–10 min. Then, the mixture was supplemented with 2.2 µL of Component A to assembly the final EOD‐CRISPR reaction which was incubated at 42 °C for 10–20 min in an Archimed X4 PCR instrument (RocGene Technology, China).

### Lyophilization System and Process

Similarly, the lyophilization system also included Component A and Component B. Component A was the dual‐uAsCas12a‐crRNA mix containing 0.2× NEB Buffer r2.1, 0.5 µm each of crRNAs, 0.2 mg mL^−1^ BSA, 0.1% (v/v) triton X‐100, 5% (m/v) trehalose, 1% (m/v) glycine, 2.5% (m/v) pullulan, 2 µm ssDNA probe, and 0.64 µM uAsCas12a. Component B was the hRPA mix contained 1× Rehydrated hRPA mix, 0.1% (v/v) triton X‐100, 5% (m/v) trehalose, 1% (m/v) glycine, and 0.5 µM each of hRPA Primers. All the concentrations were calculated based on the finally assembled reaction (10 µL). The two components were snap‐frozen at ‐80 °C overnight and then were subjected to the vacuum system for 5 h. When in use, 10 µL of rehydration buffer (8.5 µL of water, 1 µL of DNA target, and 0.5 µL of Buffer B) was added to the lyophilized Component B. Next, 10 µL Component B was added to the lyophilized Component A. Finally, the 10 µL mixture was incubated at 42 °C. In this system, different concentrations of trehalose (5%, 10%, and 15%) and glycine (1%, 2%, and 2.5%) were optimized. The optimal concentration of two lyophilized additives was determined based on fluorescence intensity. The stability of lyophilization was tested with the optimized system and placed at 25, 4, and ‐20 for two days, one week, and one month.

### Genomic DNA Extraction Using 3D‐Printed Extractor

The 3D‐printed extractor comprised binding array, mini barrel, mini pump, residue collector, and eluent collector. Binding array contained a 7‐mm diameter and 2‐mm high glassfiber membrane for nucleic acid extraction. The residue collector contained absorbent paper to absorb the waste liquids during extraction. First, it needed to homogenize samples briefly using a handhold tissue grinder. Next, 200 µL bacterial solution was mixed with 200 µL lysate and 20 µL proteinase K, then incubated at 55 °C for 10 min using the temperature control mug. Subsequently, 200 µL ethanol was added and filtered with the extractor. After washing twice (500 µL for each time) using the washing buffer, 200 µL elution buffer was added to collect the purified DNA. Details can be found in Figure 6B and Figure S15 (Supporting Information).

### Field‐Deployable 3D‐Printed Microfluidic Disc

The 3D‐printed microfluidic disc has a diameter of 3.8 cm and a height of 8 cm. The disc's 3D structure was designed using SolidWorks software and then fabricated using a Form 3 SLA 3D printer (Formlabs) with clear methacrylate‐based resin (FormsLabs, FLGPCL02). Next, the printed discs were washed using isopropanol (IPA) several times to remove the uncured resin. For ease of observation, the disc was coated using the UV shadow‐free adhesive evenly and irradiated under a UV lamp. Importantly, 10 µL hydrophobic reagent should be introduced into the channels and baked at 100 °C for 10 min to prevent solution backflow. To prevent bubbles and overflow during lyophilization on the disc, the reaction reagents should be immobilized on glassfiber matrices (D = 3mm). Prior to lyophilization, each hRPA reaction unit in central chambers and each CRISPR reaction unit in outer chambers were filled with 30 µL of the component B and 10 µL of the component B in Lyophilization System above, respectively. Post lyophilization, all the chambers were sealed using the UV adhesive. Since one disc was only adapted to one sample testing but to identify four bacteria in one sample simultaneously, the specificity of on‐disc assays was investigated in separate trials, namely in separate discs. However, to save disc's usage quantity, two discs were intentionally filled with the same lyophilized EOD‐CRISPR reagents to identify one type of bacteria. When loading various concentrations of target DNA into each central chamber, the on‐disc sensitivity of lyophilized EOD‐CRISPR assays was examined.

### Multiplex Bacteria Detection Using Onsite Detection Platform

To evaluate the feasibility of the onsite detection platform for real food sample testing, five types of synthetic food samples were purchased from local supermarkets, including canola cake, soybean meal, artificial cream, artificial meat, and peanut meal. For each food type, 3.6 mL of the sterile 1× PBS was added to 0.4 g food, followed by homogenizing manually using a handhold tissue grinder. Subsequently, the resulting solution was spiked with cultured bacteria at a final concentration of 10^3^, 10^2^, or 10^1^ CFU mL^−1^. The bacteria‐spiked solution was then subject to DNA extraction using the 3D‐printed extractor as described above. After collecting purified DNA, 30 µL of the DNA extract was loaded into each hRPA reaction unit in the central chamber to hydrate the pre‐lyophilized component B, followed by adding 3 µL of Buffer B. The central chamber was sealed immediately using the UV adhesive‐coated central sealer. After rest at ambient temperature for 5–10 min, the disc was centrifuged using the 3D‐printed mini centrifuge for 30 s. Afterwards, the disc was incubated at 42 °C for 20 min and detected in the 3D‐printed portable fluorescence detector. The detection result would be visually displayed on the detector's screen.

### Standard PCR Assays

All the used PCR primers are listed in Table S4 (Supporting Information). The primers were obtained from industry standards (SN/T 3932‐2014, SN/T 5439.1‐2022, and SN/T 5364.5‐2021) for the detection of corresponding foodborne bacteria. The standard PCR assays were conducted strictly following the commercial kits’ instructions. Prior to PCR testing, the same bacteria‐spiked solution mentioned above was subject to the spin column‐based DNA extraction using the DNeasy Blood & Tissue kit according to the manufacturer's instruction. A typical PCR reaction (10 µL) contained 5 µL of 2×AceQ Universal SYBR PCR master mix, 0.2 µM forward primer, 0.2 µM reverse primer, and 0.4 µL of the DNA extract. The program of thermal cycling was 5 min at 95 °C for initial denaturation and the 40 cycles of 15 s at 95 °C for denaturation and 30 s at 60 °C for annealing. Following it, melting curve analysis was applied to verify specific amplicons through heating post‐PCR reactions from 60 to 95 °C at a rate of 4 °C s^−1^.

### Statistical Analysis

The statistical analyses including the confusion matrixes describing the comparison of performance between two assays are conducted using the GraphPad Prism 10.2.2 software. The unpaired two‐tailed Student's t‐test in which a *P* < 0.05 indicates statistical significance is applied to analyze the significant difference between two groups. The data in histograms are presented as mean ± standard deviation of at least three replicates. Unless otherwise specified, each image involving tube‐based and disc imaging in the corresponding figure is a representative of at least two independent experiments.

## Conflict of Interest

The authors declare no conflict of interest.

## Author Contributions

X.D. designed methodology; performed investigation; performed funding acquisition; wrote the original draft. Y.S. collected the samples; performed validation and investigation; wrote the original draft; B.L. and B.M. performed validation and investigation.

## Supporting information



Supporting Information

## Data Availability

The data that support the findings of this study are available from the corresponding author upon reasonable request.

## References

[1] a) M. M. Kaminski , O. O. Abudayyeh , J. S. Gootenberg , F. Zhang , J. J. Collins , Nat. Biomed. Eng. 2021, 5, 643; 34272525 10.1038/s41551-021-00760-7

[2] a) J. S. Chen , E. Ma , L. B. Harrington , M. D. Costa , X. Tian , J. M. Palefsky , J. A. Doudna , Science 2018, 360, 436; 29449511 10.1126/science.aar6245 PMC6628903

[3] a) O. O. Abudayyeh , J. S. Gootenberg , P. Essletzbichler , S. Han , J. Joung , J. J. Belanto , V. Verdine , D. B. Cox , M. J. Kellner , A. Regev , Nature 2017, 550, 280; 28976959 10.1038/nature24049 PMC5706658

[4] H. Li , Y. Xie , F. Chen , H. Bai , L. Xiu , X. Zhou , X. Guo , Q. Hu , K. Yin , Chem. Soc. Rev. 2023, 52, 361. 36533412 10.1039/d2cs00594h

[5] a) J. P. Broughton , X. Deng , G. Yu , C. L. Fasching , V. Servellita , J. Singh , X. Miao , J. A. Streithorst , A. Granados , A. Sotomayor‐Gonzalez , Nat. Biotechnol. 2020, 38, 870; 32300245 10.1038/s41587-020-0513-4 PMC9107629

[6] Y. Zhao , F. Chen , Q. Li , L. Wang , C. Fan , Chem. Rev. 2015, 115, 12491. 26551336 10.1021/acs.chemrev.5b00428

[7] a) Y. H. Roh , C. Y. Lee , S. Lee , H. Kim , A. Ly , C. M. Castro , J. Cheon , J. H. Lee , H. Lee , Adv. Sci. 2023, 10, 2206872; 10.1002/advs.202206872 PMC10074104 36725305

[8] a) S. Gong , S. Zhang , F. Lu , W. Pan , N. Li , B. Tang , CRISPR/Cas‐Based In Vitro Diagnostic Platforms for Cancer Biomarker Detection, ACS Publications, Washington, DC 2021; 10.1021/acs.analchem.1c02533 34427091

[9] a) W. Feng , A. M. Newbigging , J. Tao , Y. Cao , H. Peng , C. Le , J. Wu , B. Pang , J. Li , D. L. Tyrrell , Chem. Sci. 2021, 12, 4683; 34163728 10.1039/d0sc06973f PMC8179559

[10] P. Ma , Q. Meng , B. Sun , B. Zhao , L. Dang , M. Zhong , S. Liu , H. Xu , H. Mei , J. Liu , Adv. Sci. 2020, 7, 2001300. 10.1002/advs.202001300 PMC7536916 33042732

[11] X. Ding , K. Yin , Z. Li , R. V. Lalla , E. Ballesteros , M. M. Sfeir , C. Liu , Nat. Commun. 2020, 11, 4711. 32948757 10.1038/s41467-020-18575-6 PMC7501862

[12] J. Joung , A. Ladha , M. Saito , N.‐G. Kim , A. E. Woolley , M. Segel , R. P. Barretto , A. Ranu , R. K. Macrae , G. Faure , N. Engl. J. Med. 2020, 383, 1492. 32937062 10.1056/NEJMc2026172 PMC7510942

[13] O. O. Abudayyeh , J. S. Gootenberg , Science 2021, 372, 914. 34045344 10.1126/science.abi9335

[14] L. T. Nguyen , N. C. Macaluso , B. L. Pizzano , M. N. Cash , J. Spacek , J. Karasek , M. R. Miller , J. A. Lednicky , R. R. Dinglasan , M. Salemi , EBioMedicine 2022, 77. 10.1016/j.ebiom.2022.103926 PMC8917962 35290826

[15] T. Notomi , H. Okayama , H. Masubuchi , T. Yonekawa , K. Watanabe , N. Amino , T. Hase , Nucleic Acids Res. 2000, 28, 63e. 10.1093/nar/28.12.e63 PMC102748 10871386

[16] A. Mahas , T. Marsic , M. Lopez‐Portillo Masson , Q. Wang , R. Aman , C. Zheng , Z. Ali , M. Alsanea , A. Al‐Qahtani , B. Ghanem , Proc. Natl. Acad. Sci. USA 2022, 119, 2118260119. 10.1073/pnas.2118260119 PMC9282225 35763567

[17] J. S. Park , K. Hsieh , L. Chen , A. Kaushik , A. Y. Trick , T. H. Wang , Adv. Sci. 2021, 8, 2003564. 10.1002/advs.202003564 PMC7927608 33717855

[18] X. Ding , K. Yin , Z. Li , M. M. Sfeir , C. Liu , Biosens. Bioelectron. 2021, 184, 113218. 33878591 10.1016/j.bios.2021.113218 PMC8052607

[19] a) J. Jiao , Y. Liu , M. Yang , J. Zheng , C. Liu , W. Ye , S. Song , T. Bai , C. Song , M. Wang , Plant Biotechnol. J. 2023, 21, 1465; 37069831 10.1111/pbi.14051 PMC10281610

[20] L. Zhang , J. A. Zuris , R. Viswanathan , J. N. Edelstein , R. Turk , B. Thommandru , H. T. Rube , S. E. Glenn , M. A. Collingwood , N. M. Bode , Nat. Commun. 2021, 12, 3908. 34162850 10.1038/s41467-021-24017-8 PMC8222333

[21] a) W. Abu Al‐Soud , P. Rådstrom , J. Clin. Microbiol. 2000, 38, 4463; 11101581 10.1128/jcm.38.12.4463-4470.2000 PMC87622

[22] X. Feng , M. M. Spiering , R. de Luna Almeida Santos , S. J. Benkovic , H. Li , Nat. Commun. 2023, 14, 4396. 37474605 10.1038/s41467-023-40106-2 PMC10359460

[23] W. H. O., (WHO) 2015, https://www.who.int/publications/i/item/9789241565165, (accessed: December 2015)

[24] a) A. Sayad , F. Ibrahim , S. M. Uddin , J. Cho , M. Madou , K. L. Thong , Biosens. Bioelectron. 2018, 100, 96; 28869845 10.1016/j.bios.2017.08.060

[25] J. Liu , H. Wang , L. Zhang , Y. Lu , X. Wang , M. Shen , N. Li , L. Feng , J. Jing , B. Cao , Small 2022, 18, 2200854. 10.1002/smll.202200854 35599436

[26] K. Kadimisetty , J. Song , A. M. Doto , Y. Hwang , J. Peng , M. G. Mauk , F. D. Bushman , R. Gross , J. N. Jarvis , C. Liu , Biosens. Bioelectron. 2018, 109, 156. 29550739 10.1016/j.bios.2018.03.009 PMC6172948

[27] Y. M. Hassan , A. S. Mohamed , Y. M. Hassan , W. M. El‐Sayed , Clin. Exp. Med. 2025, 25, 33. 39789283 10.1007/s10238-024-01540-8 PMC11717804

[28] C. Saisawang , P. Naksith , S. Sakdee , A. J. Ketterman , S. Tuntithavornwat , P. Nimsamer , O. Mayuramart , N. Chantaravisoot , T. Pisitkun , S. Payungporn , KIJOMS 2023, 9, 4.

[29] D. Cravero , F. Cerutti , M. G. Maniaci , P. Barzanti , S. Scaramagli , M. V. Riina , F. Ingravalle , P. L. Acutis , S. Peletto , Lwt 2019, 106, 64.

[30] L. Samie , C. Champod , V. Glutz , M. Garcia , V. Castella , F. Taroni , Sci. Justice 2019, 59, 405. 31256811 10.1016/j.scijus.2019.02.003

[31] a) M. Hu , Z. Qiu , Z. Bi , T. Tian , Y. Jiang , X. Zhou , Proc. Natl. Acad. Sci. 2022, 119, 2202034119; 10.1073/pnas.2202034119 PMC9245704 35727982

[32] A. Homchan , M. Patchsung , P. Chantanakool , T. Wongsatit , W. Onchan , D. Muengsaen , T. Thaweeskulchai , M. Tandean , T. Sakpetch , S. Suraritdechachai , J. Am. Chem. Soc. 2025, 147, 10088. 39948709 10.1021/jacs.4c11893 PMC11951158

[33] M. Lin , H. Yue , T. Tian , E. Xiong , D. Zhu , Y. Jiang , X. Zhou , Anal. Chem. 2022, 94, 8277. 35635176 10.1021/acs.analchem.2c00616

[34] K. Yin , X. Ding , Z. Li , H. Zhao , K. Cooper , C. Liu , Anal. Chem. 2020, 92, 8561. 32390420 10.1021/acs.analchem.0c01459 PMC7588651

[35] S. Lu , X. Tong , Y. Han , K. Zhang , Y. Zhang , Q. Chen , J. Duan , X. Lei , M. Huang , Y. Qiu , Nat. Biomed. Eng. 2022, 6, 286. 35314803 10.1038/s41551-022-00861-x

[36] a) M. Tang , G. Wang , S.‐K. Kong , H.‐P. Ho , Micromachines 2016, 7, 26; 30407398 10.3390/mi7020026 PMC6190084

[37] a) I. Maguire , R. O'kennedy , J. Ducrée , F. Regan , Anal. Methods 2018, 10, 1497;

[38] J. Lim , A. B. Van , K. Koprowski , M. Wester , E. Valera , R. Bashir , Proc. Natl. Acad. Sci. 2025, 122, 2420166122. 10.1073/pnas.2420166122 PMC11929484 40063799

[39] a) M. J. Kachwala , F. Hamdard , D. Cicek , H. Dagci , C. W. Smith , N. Kalla , M. V. Yigit , Adv. Healthcare Mater. 2024, 13, 2400508; 10.1002/adhm.202400508 38683016

[40] D. Cai , Y. Wang , Z. Zhang , E. Huang , N. Yang , X. Yang , T. Zhang , H. Wen , Y. Wang , Z. Chen , Biosens. Bioelectron. 2025, 276, 117256. 39970723 10.1016/j.bios.2025.117256

