# Supplementary material for: Enhanced One‐Pot Dual‐CRISPR‐Based Assay Lyophilized on a 3D‐Printed Disc for Field‐Deployable Multiplex Bacteria Detection
Source: Adv Sci (Weinh). 2025 Sep 16;12(45):e09355. doi: 10.1002/advs.202509355 (PMC12677630; doi:10.1002/advs.202509355)
Supplement: Supplementary file 1 — Supporting Information [file ADVS-12-e09355-s001.docx]

Supporting Information

**Enhanced One-pot Dual-CRISPR-based Assay** **Lyophilized on a 3D-printed** **Disc for** **Field-deployable Multiplex Bacteria Detection**

*Yuqing Shen, Bo Lu, Biao Ma, and Xiong Ding ^*^*

**Yuqing Shen and Xiong Ding***

Key Laboratory of Environmental Medicine and Engineering, Ministry of Education, School of Public Health

Department of Nutrition and Food Hygiene, School of Public Health

Southeast University

Nanjing 210009, P. R. China

*****E-mail: [xiongdlab21@seu.edu.cn](mailto:xiongdlab21@seu.edu.cn)

**Bo Lu and Biao Ma**

State Key Laboratory of Digital Medical Engineering, School of Biological Science and Medical Engineering, Southeast University

Nanjing 210009, P. R. China

**Table of Contents**

[Figure S1. Effect of various concentrations of BSA, Tariune, Tween-20, and Guanidine-HCl on EOD-CRISPR assays 4](#_Toc204947233)

[Figure S2. Effect of various concentrations of Trehalose, Pullulan, and Triton X-100 on EOD-CRISPR assays 5](#_Toc204947234)

[Figure S3. Effect of various concentrations of L-Proline, Betaine, and Glycerol on EOD-CRISPR assays 6](#_Toc204947235)

[Figure S4. Effect of various concentrations of Urea, Glycine, and DMSO on EOD-CRISPR assays 7](#_Toc204947236)

[Figure S5. Fluorescence imaging of tube-based EOD-CRISPR assays with various concentrations of 13 additives 8](#_Toc204947237)

[Figure S6. Analysis of uAsCas12a nuclease 9](#_Toc204947238)

[Figure S7. Comparison of real-time fluorescence changes of the EOD-CRISPR assays with LbaCas12a and uAsCas12a when testing various concentrations of *S. aureus* genomic DNA 10](#_Toc204947239)

[Figure S8. Sensitivity testing of EOD-CRISPR assays using various concentrations (CFU/mL) of extracted gDNA from corresponding bacteria 11](#_Toc204947240)

[Figure S9. Structure of 3D-printed microfluidic disc 12](#_Toc204947241)

[Figure S10. Sensitivities of on-disc EOD-CRISPR assays through fluorescence imaging for four foodborne bacteria detection 13](#_Toc204947242)

[Figure S11. Sensitivities of four foodborne pathogens using the commercial qPCR assays 14](#_Toc204947243)

[Figure S12. Specificities of on-disc EOD-CRISPR assays through fluoresence imaging for four foodborne bacteria detection 15](#_Toc204947244)

[Figure S13. The 3D-printed extractor for DNA extraction and purification. A) The assembly units. B) Extraction rate of the 3D-printed extractor when using 10^1^, 10^0^, and 10^-1^ ng/μL of S. aureus genomic DNA. Three replicates (n=3) were run for each test. 16](#_Toc204947245)

[Figure S14. The 3D-printed housing of A) portable fluorescence detector and B) mini centrifuge. 17](#_Toc204947246)

[Figure S15. The procedures of sample treatment and DNA extraction using the 3D-printed extractor 18](#_Toc204947247)

[Figure S16. The results of disc imaging and portable fluorescence detector for *B. cereus* detection from five types of synthetic food including canola cake (A), soybean meal (B), artificial cream (C), artificial meat (D), and peanut meal (E). 21](#_Toc204947248)

[Figure S17. The results of disc imaging and portable fluorescence detector for *Salmonella* detection from five types of synthetic food including canola cake (A), soybean meal (B), artificial cream (C), artificial meat (D), and peanut meal (E). 24](#_Toc204947249)

[Figure S18. The results of disc imaging and portable fluorescence detector for *S. aureus* detection from five types of synthetic food including canola cake (A), soybean meal (B), artificial cream (C), artificial meat (D), and peanut meal (E). 27](#_Toc204947250)

[Figure S19. The results of disc imaging and portable fluorescence detector for *E. coli* O157:H7 detection from five types of synthetic food including canola cake (A), soybean meal (B), artificial cream (C), artificial meat (D), and peanut meal (E). 30](#_Toc204947251)

[Figure S20. The results of disc imaging and portable fluorescence detector for the detection of *B. cereus, Salmonella, S. aureus,* and *E. coli O157:H7* detection from pork and chicken meats 31](#_Toc204947252)

[Figure S21. Schematic illustration of emergency coping strategies for outbreak of foodborne bacterial contamination event 32](#_Toc204947253)

[Table S1. The list of conserved sequences for *B. cereus, Salmonella, E. coli* O157:H7*,* and *S. aureus* 33](#_Toc204947254)

[Table S2. The list of RPA primers for *B. cereus, Salmonella, E. coli* O157:H7*,* and *S. aureus* 37](#_Toc204947255)

[Table S3. The list of crRNA and probe for *B. cereus, Salmonella, E. coli* O157:H7*,* and *S. aureus* 38](#_Toc204947256)

[Table S4. The list of PCR primers for *B. cereus, Salmonella, E. coli* O157:H7*,* and *S. aureus* 39](#_Toc204947257)

[Table S5. The Cq values of the qPCR method for detecting *B. cereus* 40](#_Toc204947258)

[Table S6. The Cq values of the qPCR method for detecting *Salmonella* 41](#_Toc204947259)

[Table S7. The Cq values of the qPCR method for detecting *S. aureus* 42](#_Toc204947260)

[Table S8. The Cq values of the qPCR method for detecting *E. coli* O157:H7 43](#_Toc204947261)

[Table S9. Comparison of currently reported one-pot RPA-CRISPR/12a assays with the EOD-CRISPR assay 44](#_Toc204947262)

[References…………………………………………………………………………………………45](#_Toc204947263)


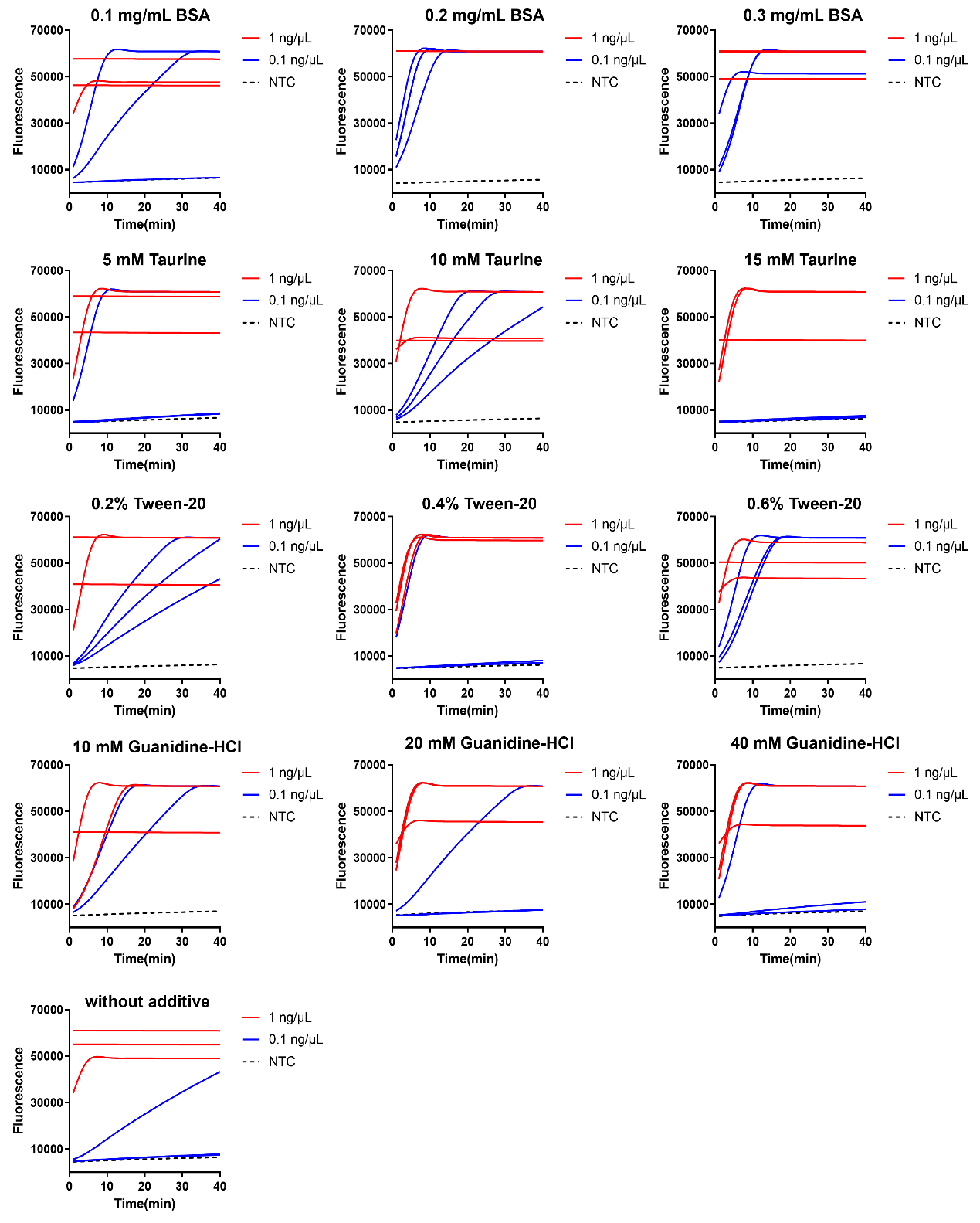


# **Figure S1. Effect of various concentrations of BSA, Tariune, Tween-20, and Guanidine-HCl on EOD-CRISPR assays.** The assays with 1 and 0.1 ng/μL of *S. aureus* genomic DNA were conducted. Three replicates (n = 3) were run for each test. NTC, no-target control.


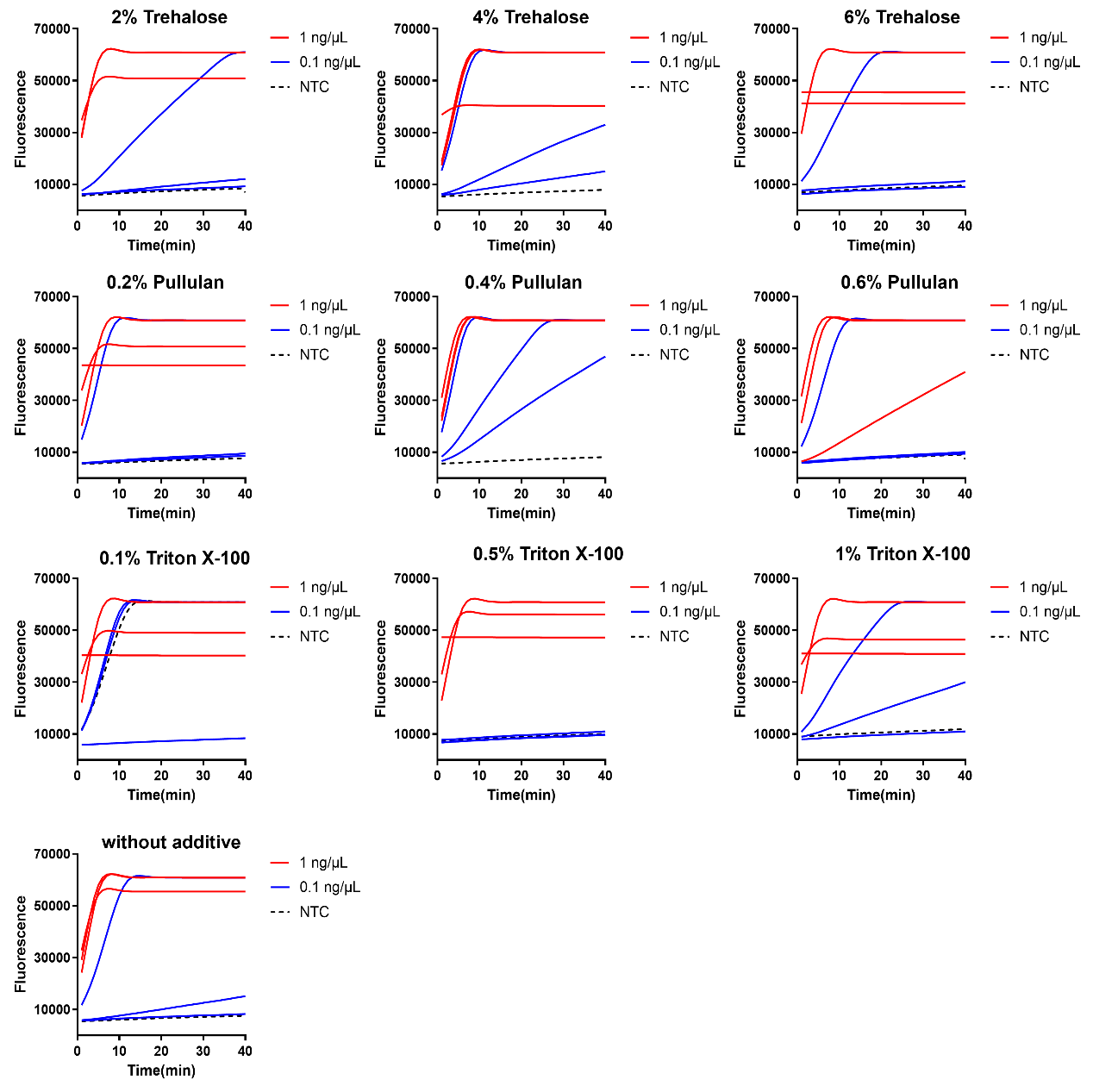


# **Figure S2. Effect of various concentrations of Trehalose, Pullulan, and Triton X-100 on EOD-CRISPR assays.** The assays with 1 and 0.1 ng/μL of *S. aureus* genomic DNA were conducted. Three replicates (n = 3) were run for each test. NTC, no-target control.


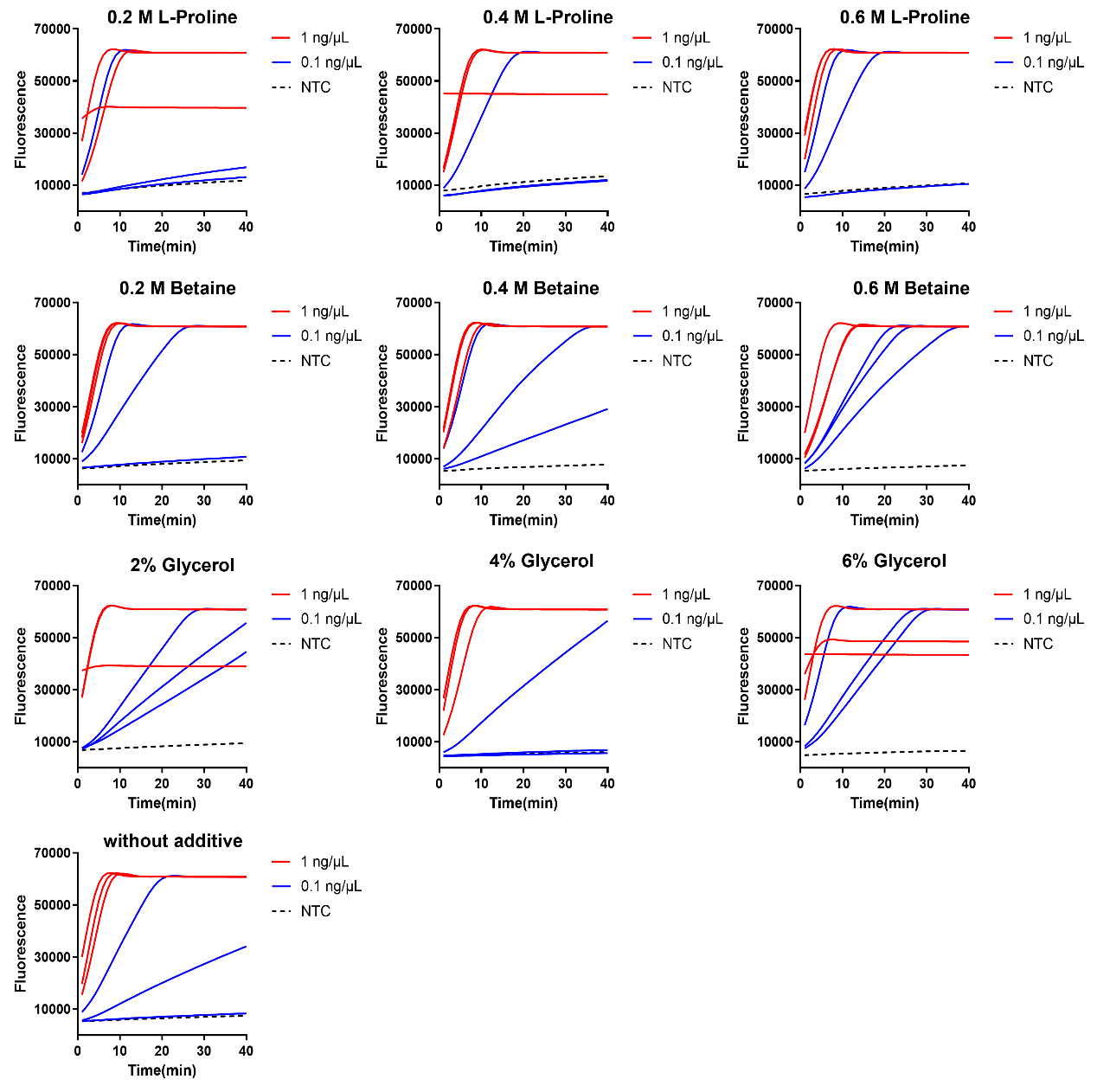


# **Figure S3. Effect of various concentrations of L-Proline, Betaine, and Glycerol on EOD-CRISPR assays.** The assays with 1 and 0.1 ng/μL of *S. aureus* genomic DNA were conducted. Three replicates (n = 3) were run for each test. NTC, no-target control.


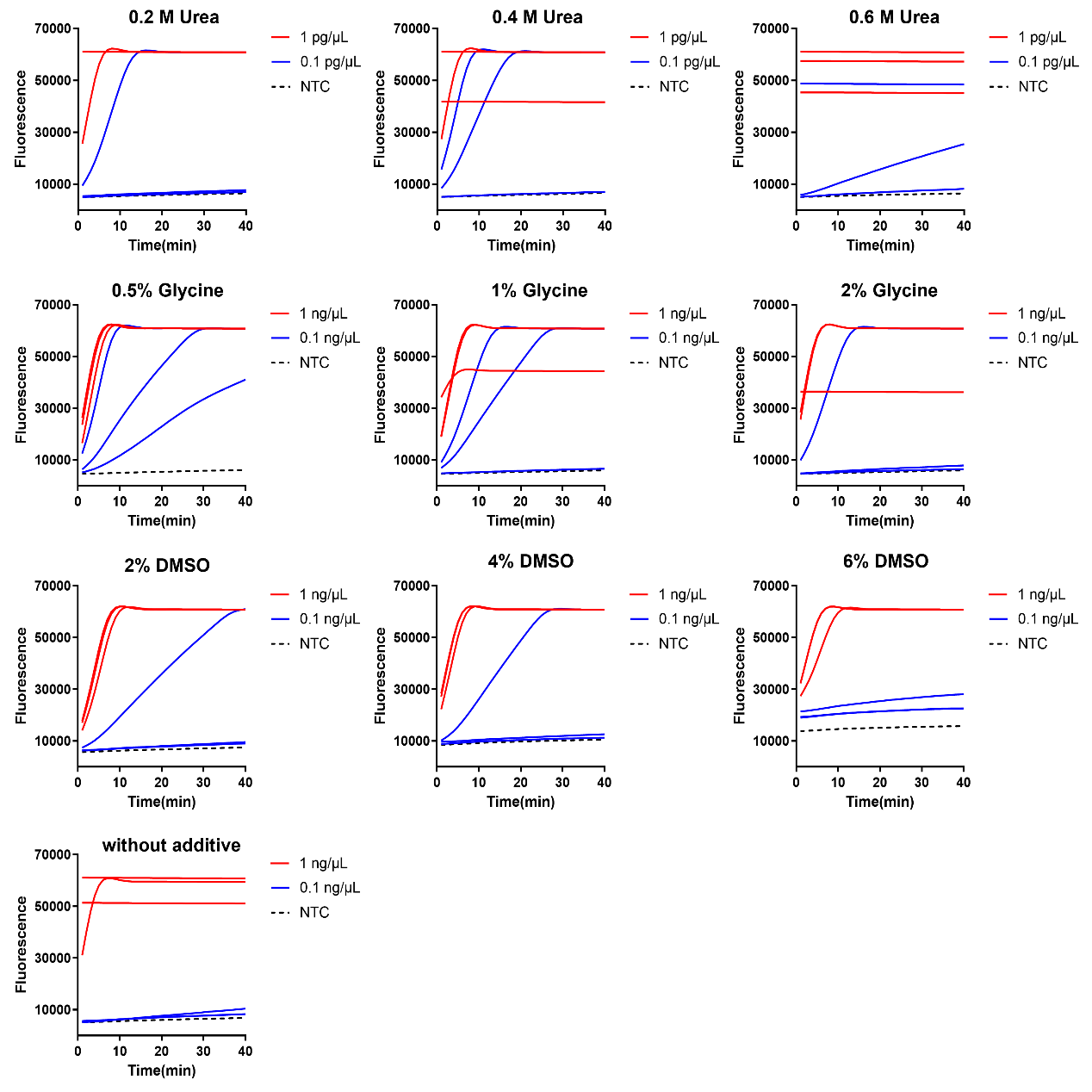


# **Figure S4. Effect of various concentrations of Urea, Glycine, and DMSO on EOD-CRISPR assays.** The assays with 1 and 0.1 ng/μL of *S. aureus* genomic DNA were conducted. Three replicates (n = 3) were run for each test. NTC, no-target control.


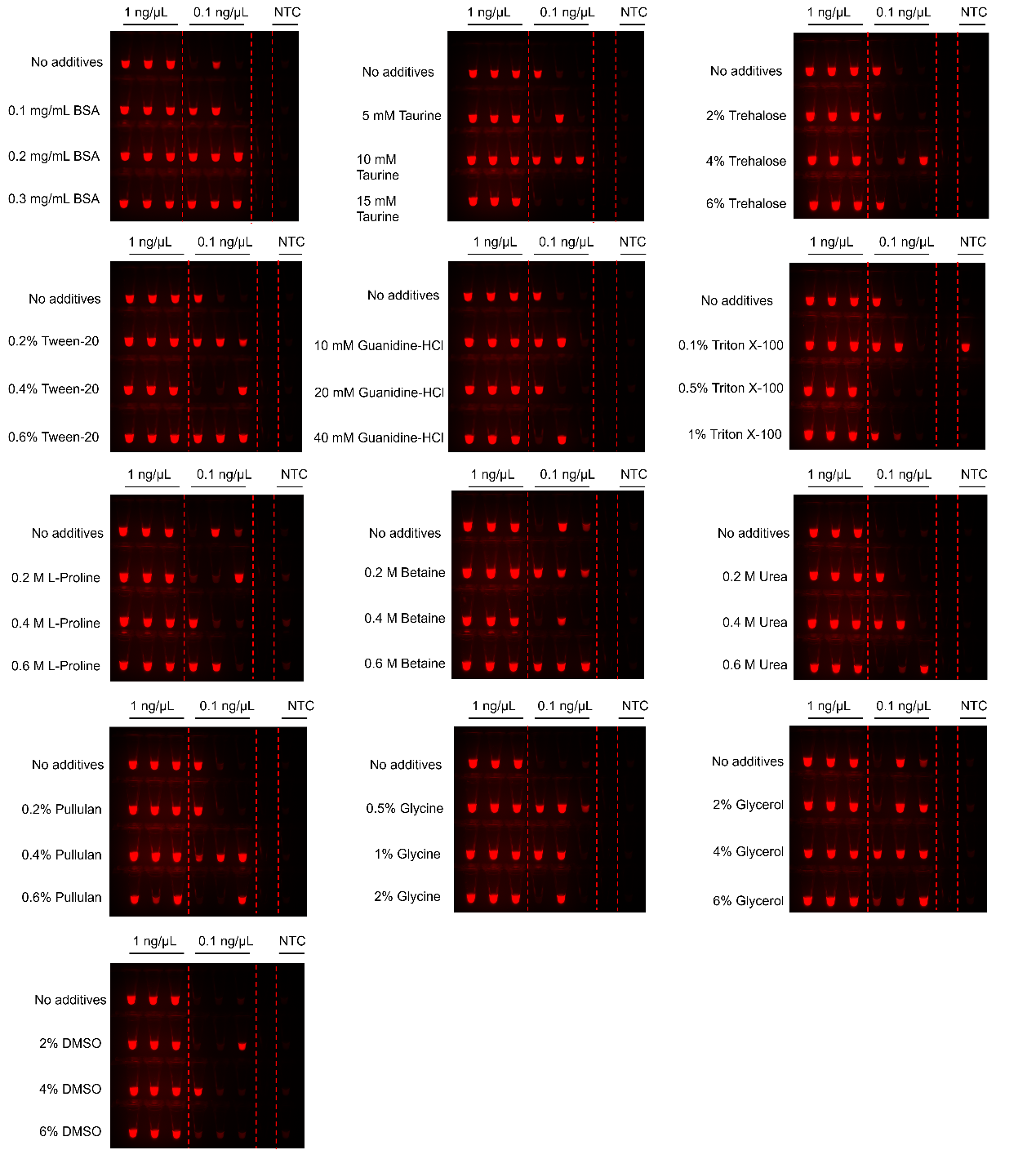


# **Figure S5. Fluorescence imaging of tube-based EOD-CRISPR assays with various concentrations of 13 additives.** The assays with 1 and 0.1 ng/μL of *S. aureus* genomic DNA were conducted. Three replicates (n = 3) were run for each test. NTC, no-target control.


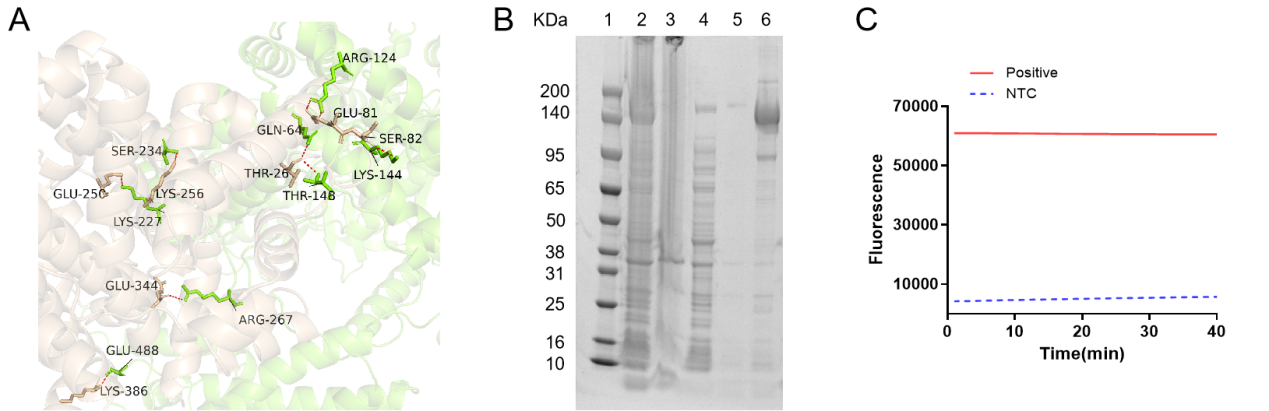


# **Figure S6. Analysis of uAsCas12a nuclease. A)** The interaction between BSA and uAsCas12a (light green) analyzed using AlphaFold Server. The amino acids with polar contacts were indicated. **B)** SDS-PAGE gel electrophoresis of uAsCas12a nuclease. 1, marker; 2, crude enzyme; 3, crude enzyme precipitation; 4, resin flow-through; 5, unbound protein; 6, elution by 200 mM imidazole. **C)** The real-time fluorescence EOD-CRISPR assay using purified uAsCas12a nuclease. Positive, the assay with 1 ng of *S. aureus* genomic DNA. NTC, no-target control.


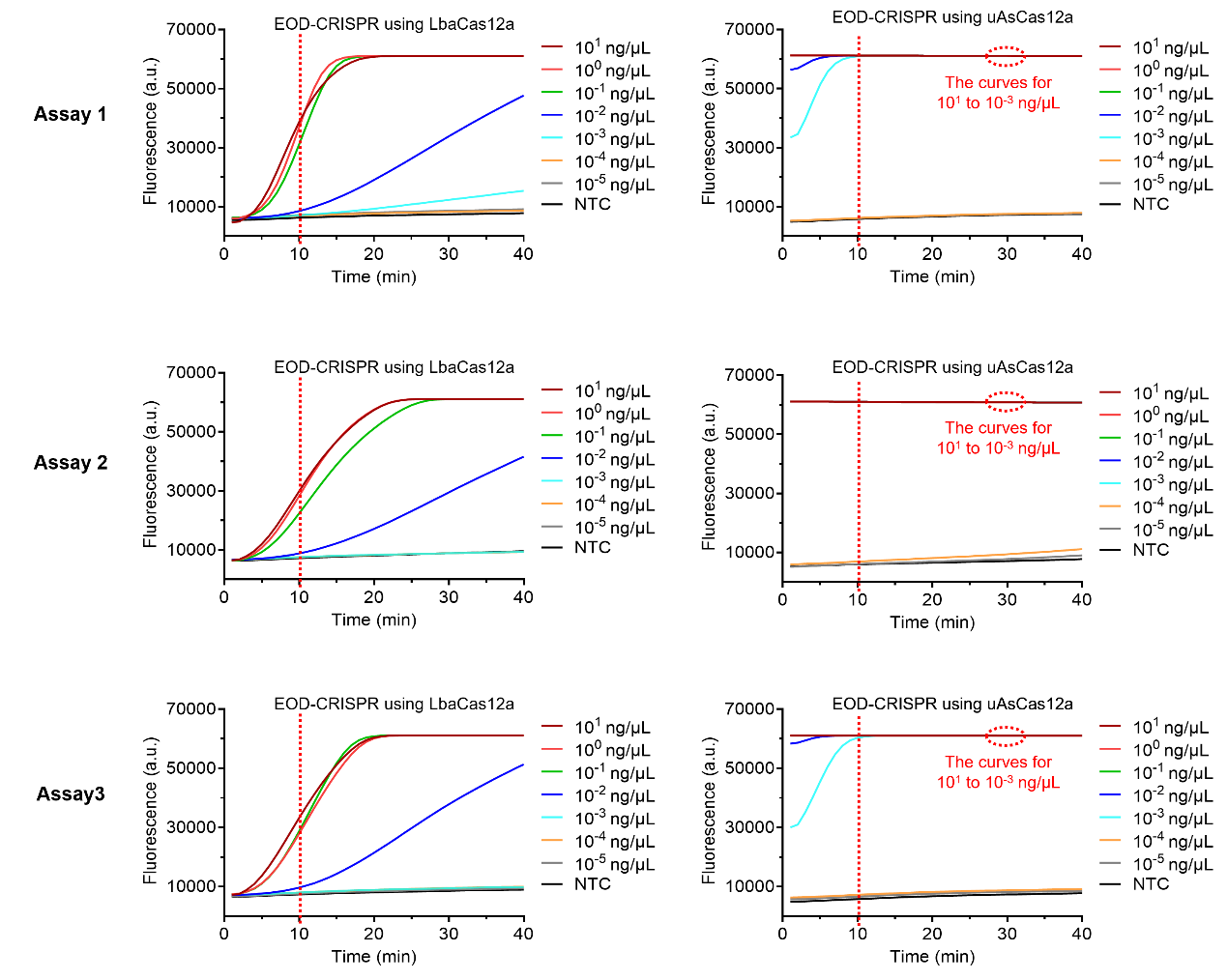


# **Figure S7. Comparison of real-time fluorescence changes of the EOD-CRISPR assays with LbaCas12a and uAsCas12a when testing various concentrations of *S. aureus* genomic DNA**. The vertical dotted line presents the point of 10-min incubation. NTC, no-target control.


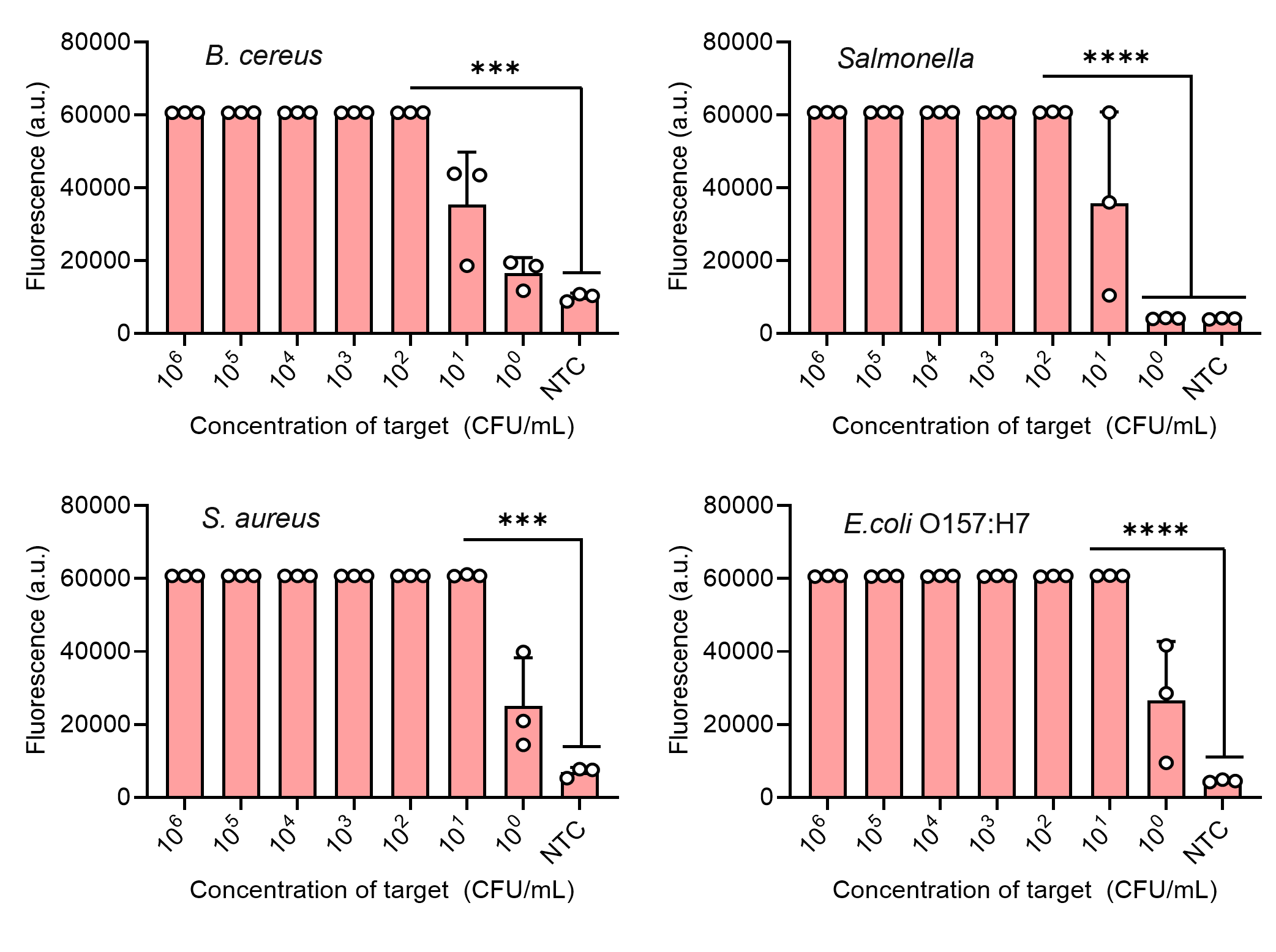


# **Figure S8.** **Sensitivity testing of EOD-CRISPR assays using various concentrations (CFU/mL) of extracted gDNA from corresponding** **bacteria.** Fluorescence imaging was conducted at the end of reaction. All the tests were incubated at 42ºC for 20 min. Three replicates (n = 3) were run for each test. Error bars represent the standard deviations of the three replicates. Unpaired two-tailed t-test was used to analyse the significant difference between two groups. ***, *p* < 0.001; ****, *p* < 0.0001


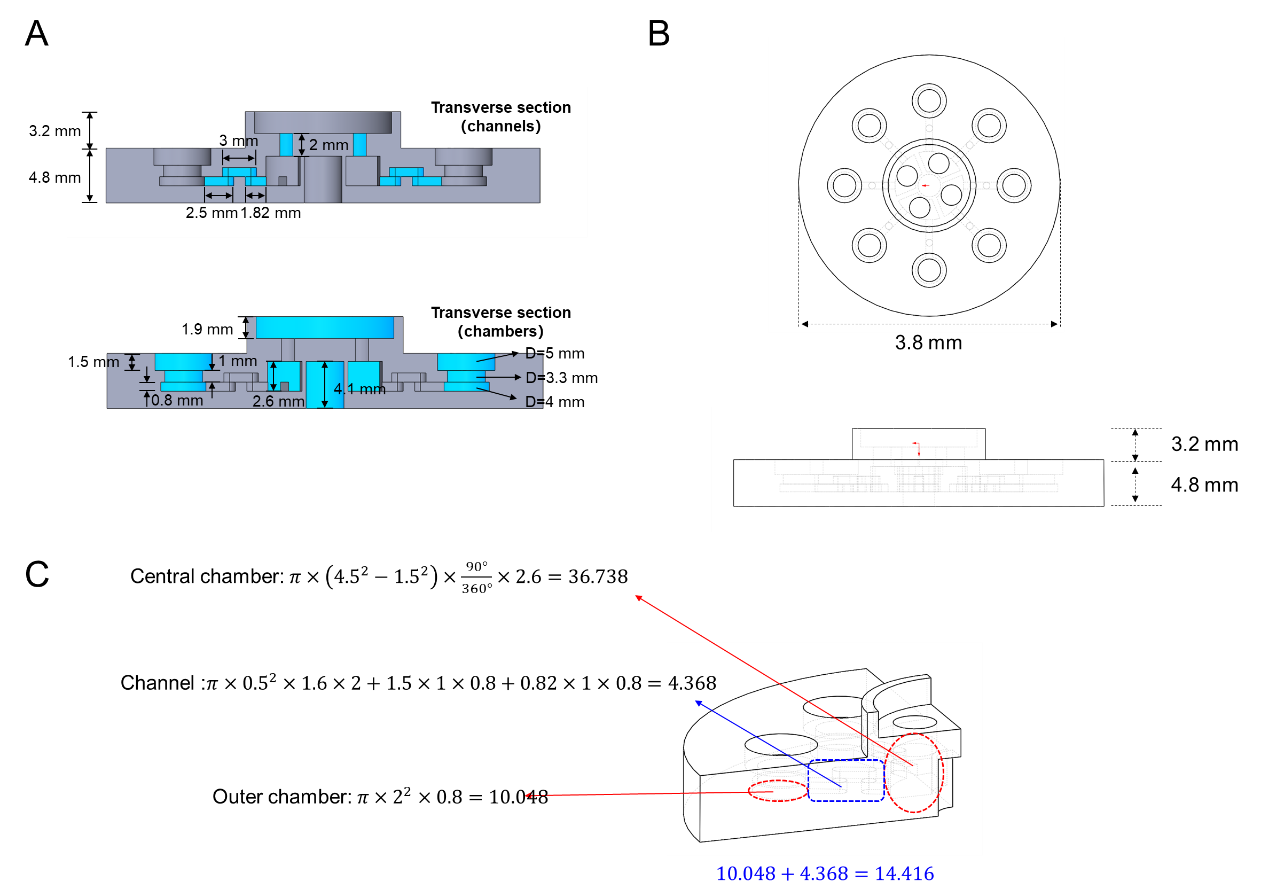


# **Figure S9. Structure of 3D-printed microfluidic disc. A)** Transverse section of channel and chamber dimensions. **B)** Dimension of 3D-printed microfluidic disc. **C)** Volume of chambers and channels, measured in microliters.

**
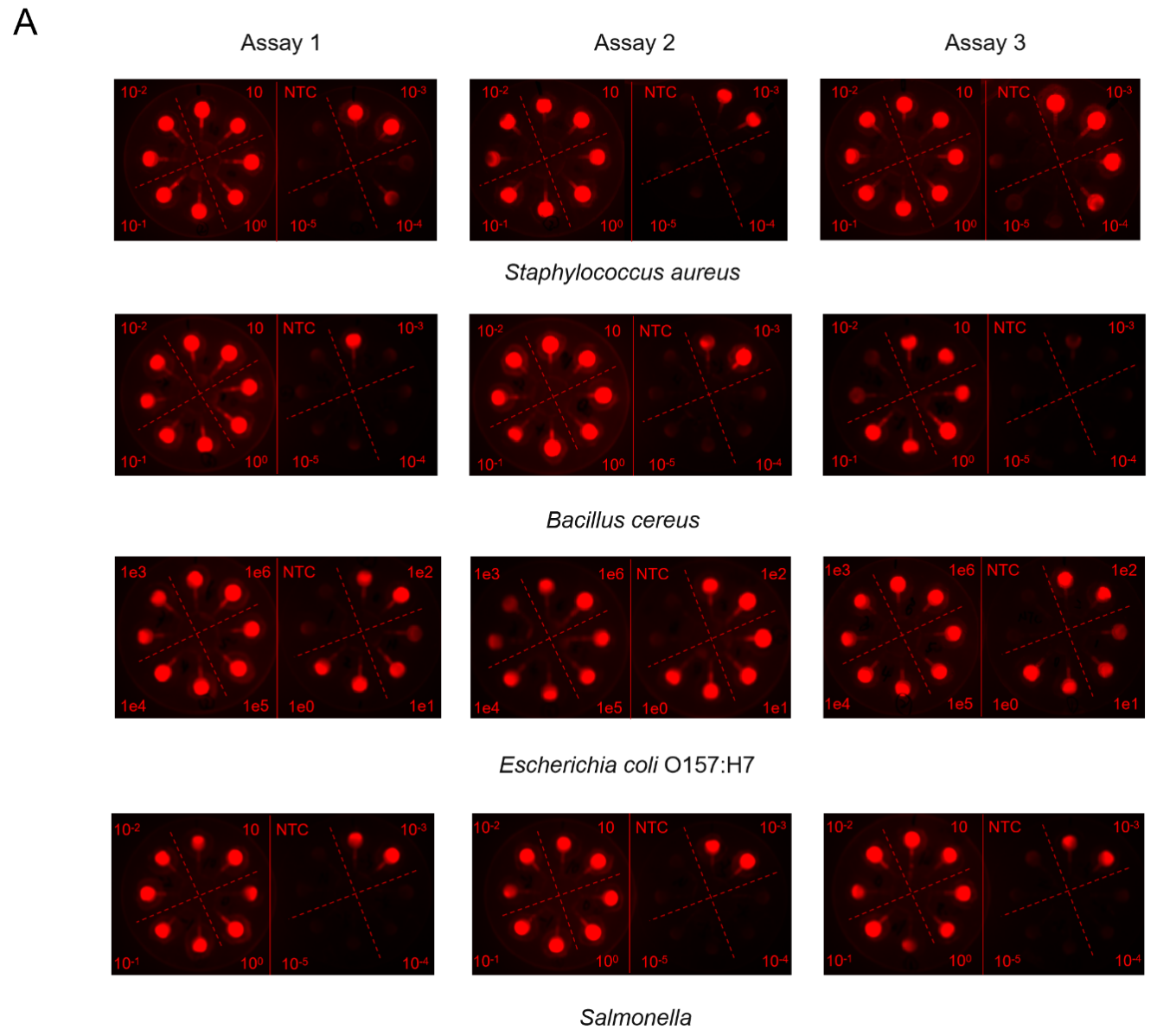
**

# **Figure S10. Sensitivities of on-disc EOD-CRISPR assays through fluorescence imaging for four foodborne bacteria detection.** NTC, no-target control. Three replicates (n=3) were run for each test. Various concentrations of extracted genomic DNA from corresponding bacteria were used as targets.


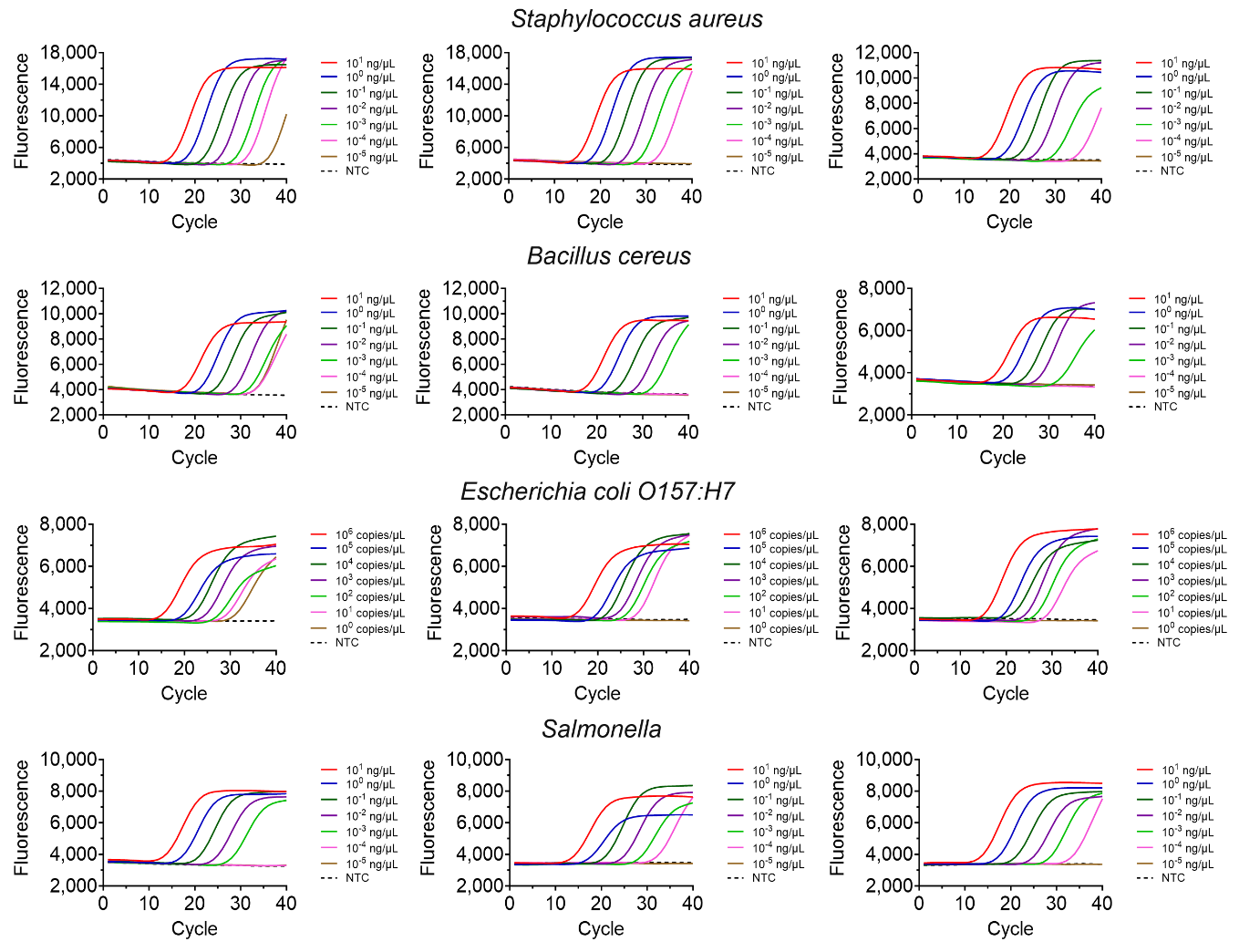


# **Figure S11. Sensitivities of four foodborne pathogens using the commercial qPCR assays.** NTC, no-target control. Three replicates (n=3) were run for each test. Various concentrations of extracted genomic DNA from corresponding bacteria were used as targets.


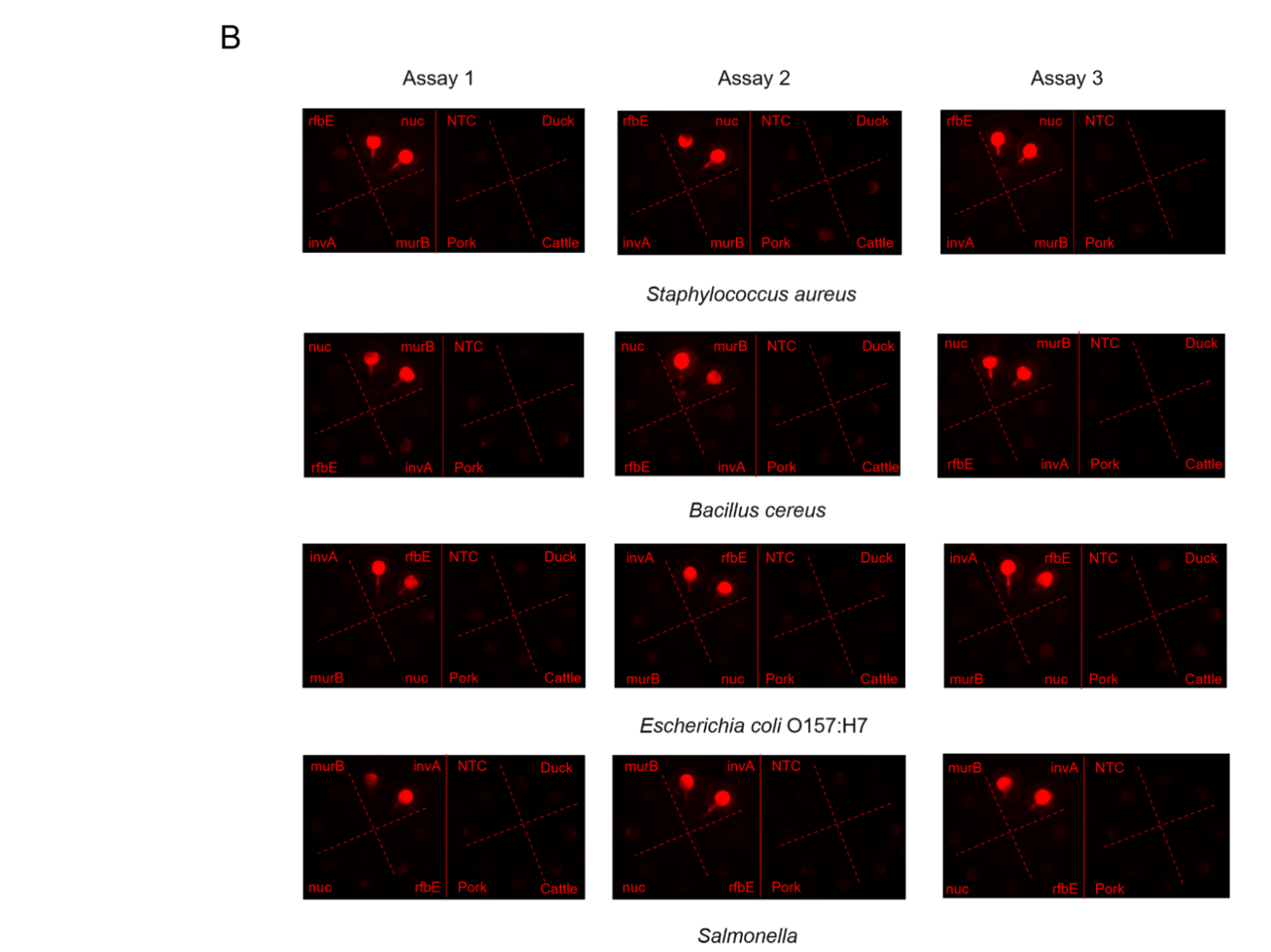


# **Figure S12. Specificities of on-disc EOD-CRISPR assays through fluoresence imaging for four foodborne bacteria detection.** NTC, no-target control. Three replicates (n=3) were run for each test. The loaded target was 1 ng/μL of extracted genomic DNA from corresponding bacteria.


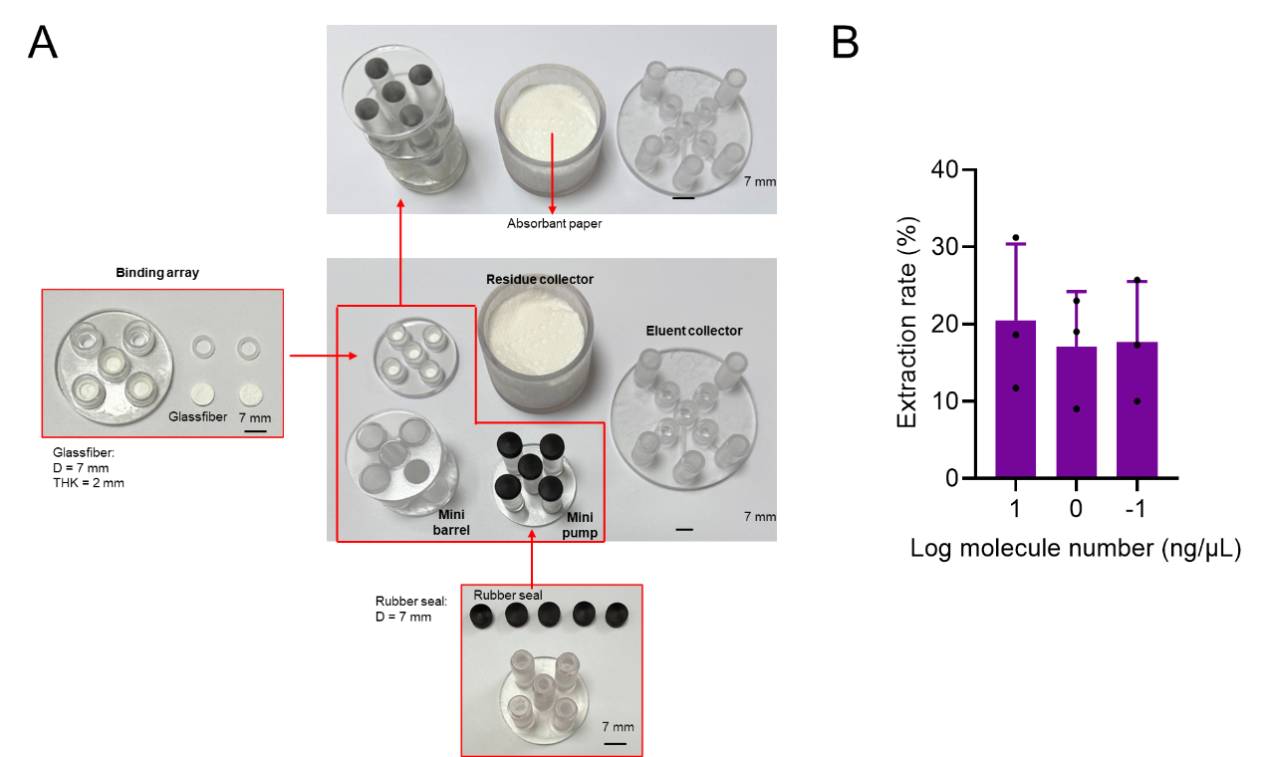


# **Figure S13. The 3D-printed extractor for DNA extraction and purification. A)** The assembly units. **B)** Extraction rate of the 3D-printed extractor when using 10^1^, 10^0^, and 10^-1^ ng/μL of S. aureus genomic DNA. Three replicates (n=3) were run for each test.


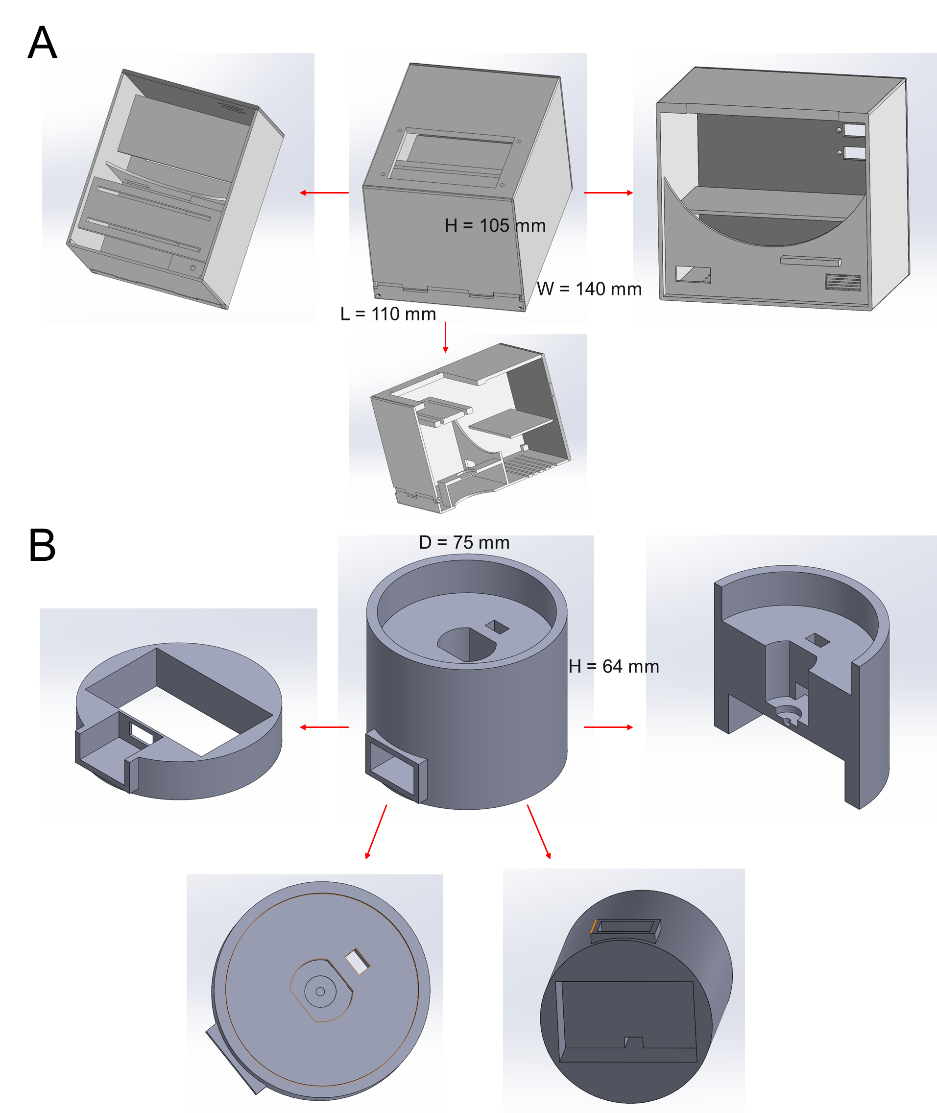


# **Figure S14. The 3D-printed housing of A) portable fluorescence detector and B) mini centrifuge.**


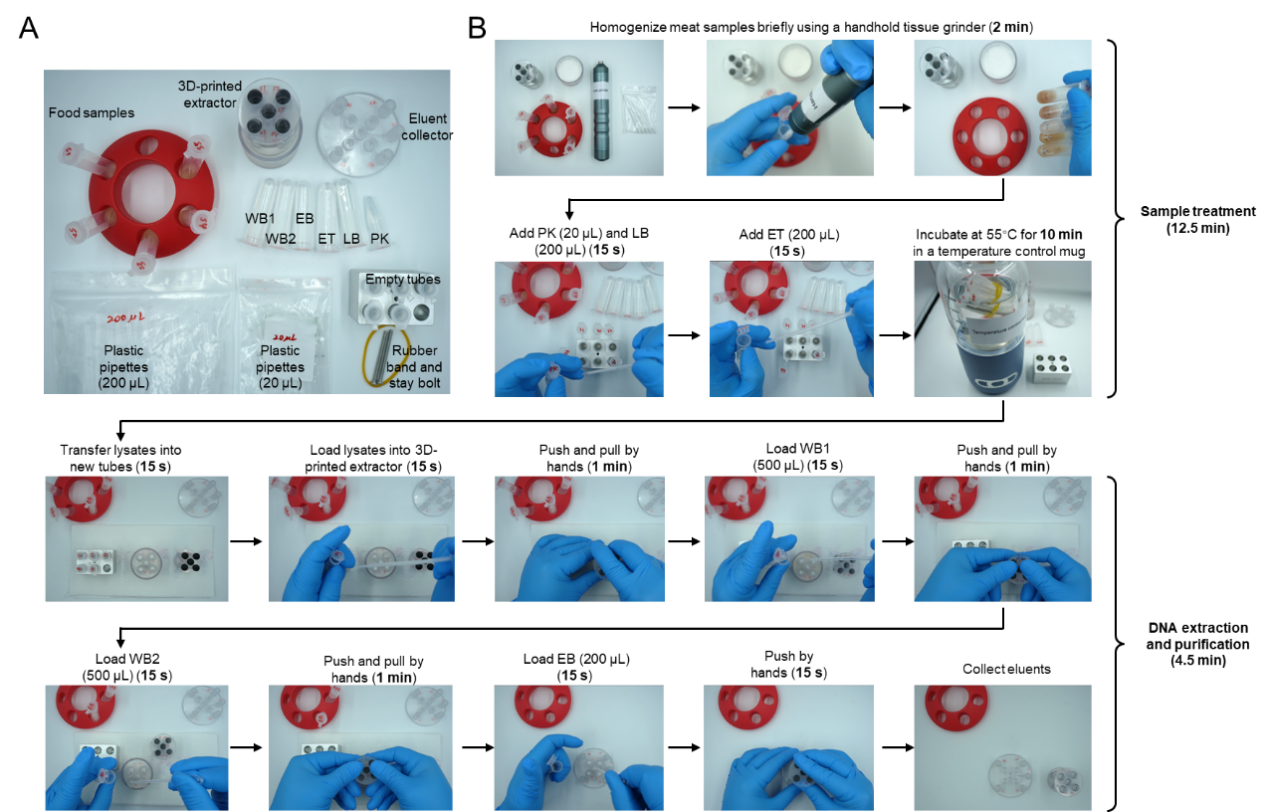


# **Figure S15. The procedures of sample treatment and DNA extraction using the 3D-printed extractor**. A) The requried materials. B) The step-by-step operation from sample homogenization to DNA eluent collection. WB1, wash buffer 1; WB2, wash buffer 2; EB, eluent buffer; ET, ethanol; LB, lysis buffer; PK, proteinase K.


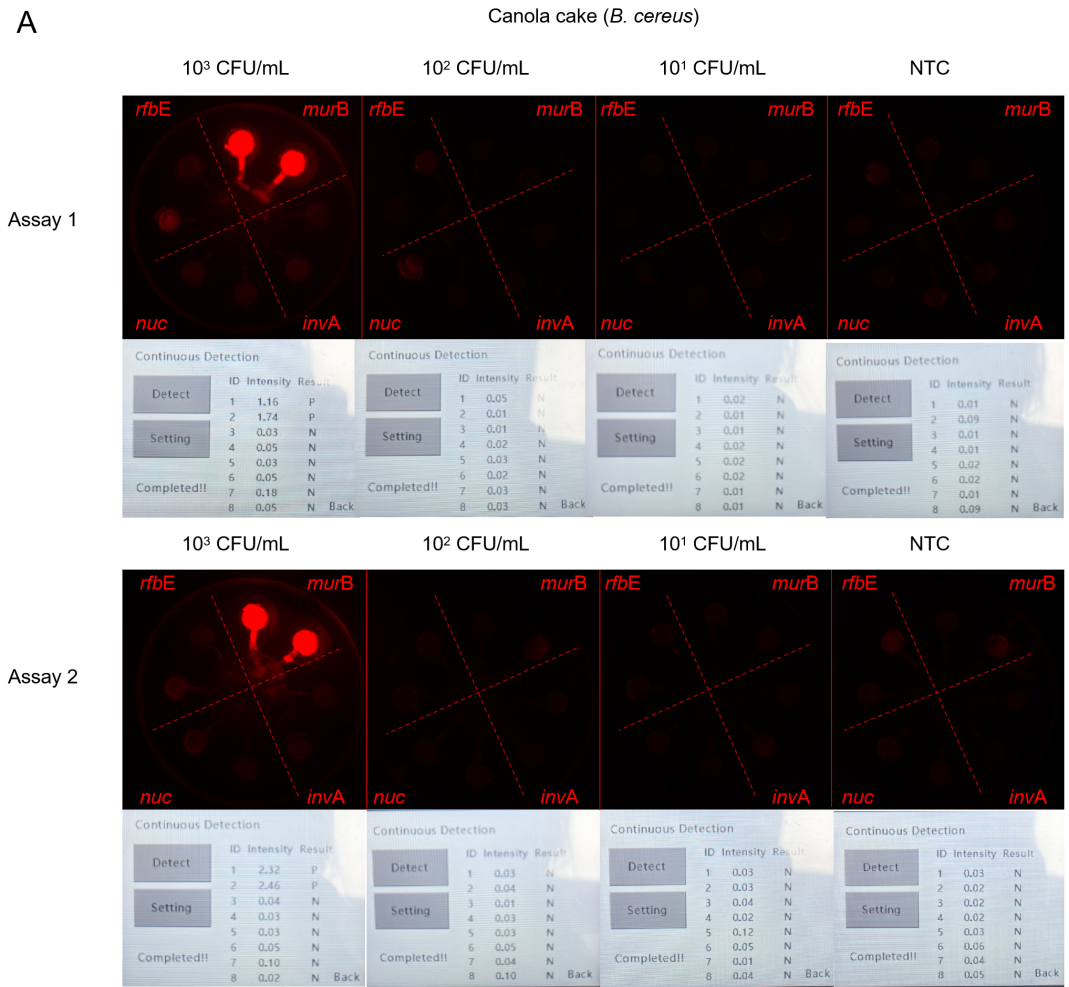


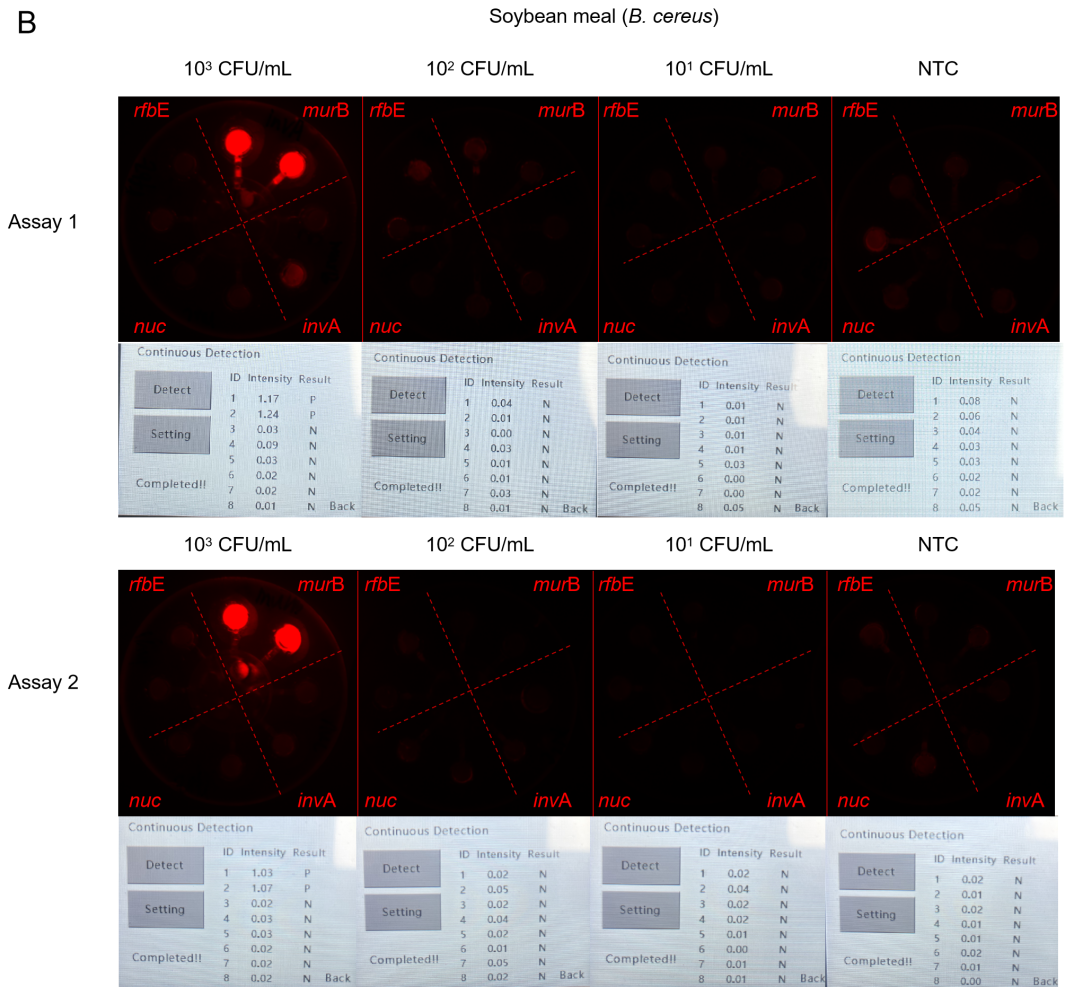


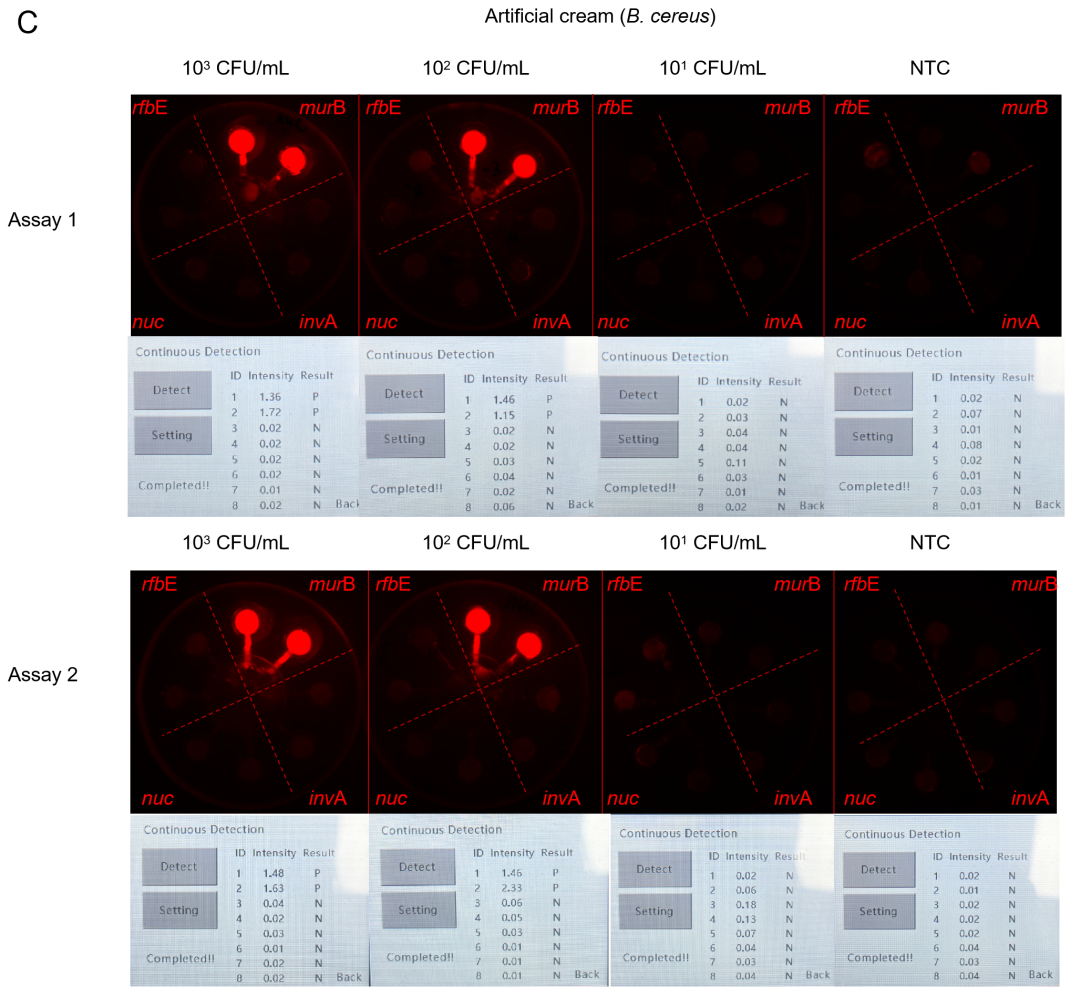


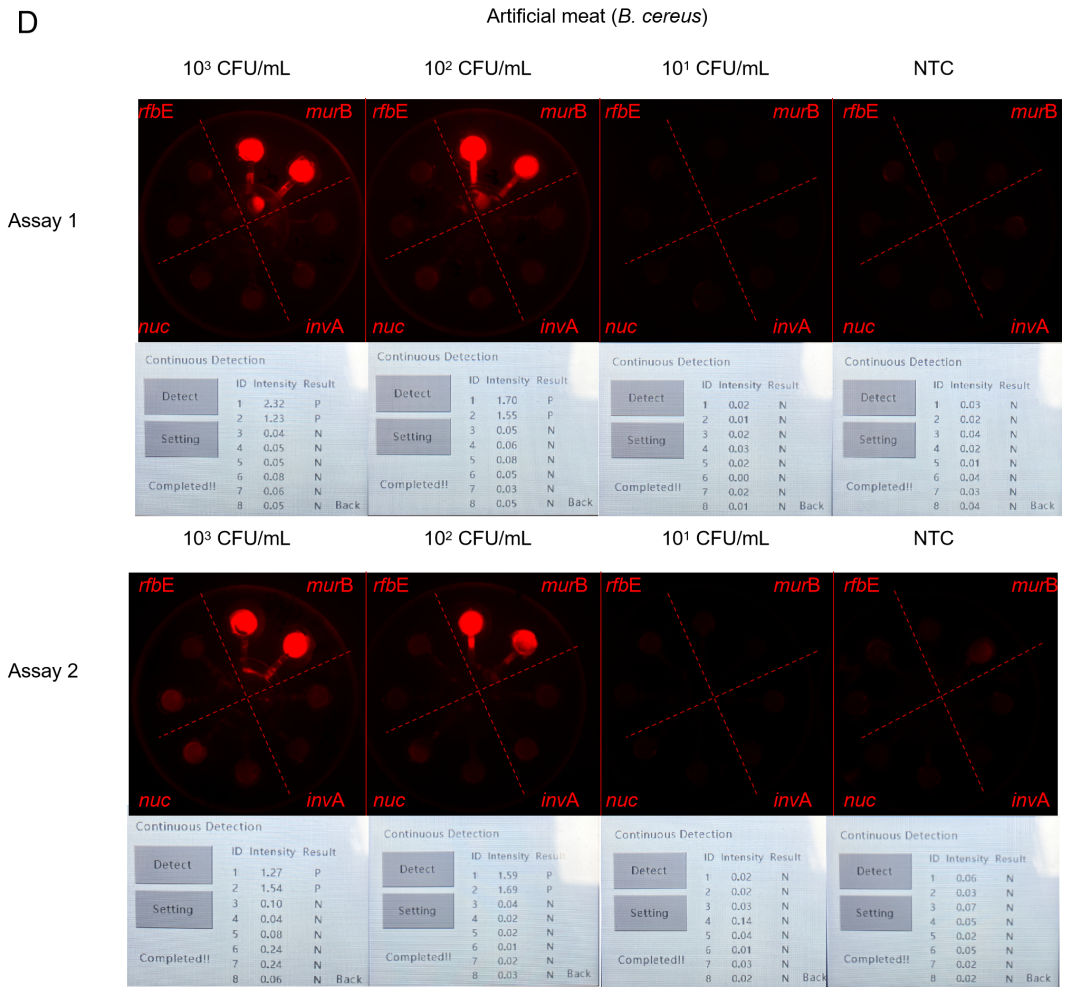


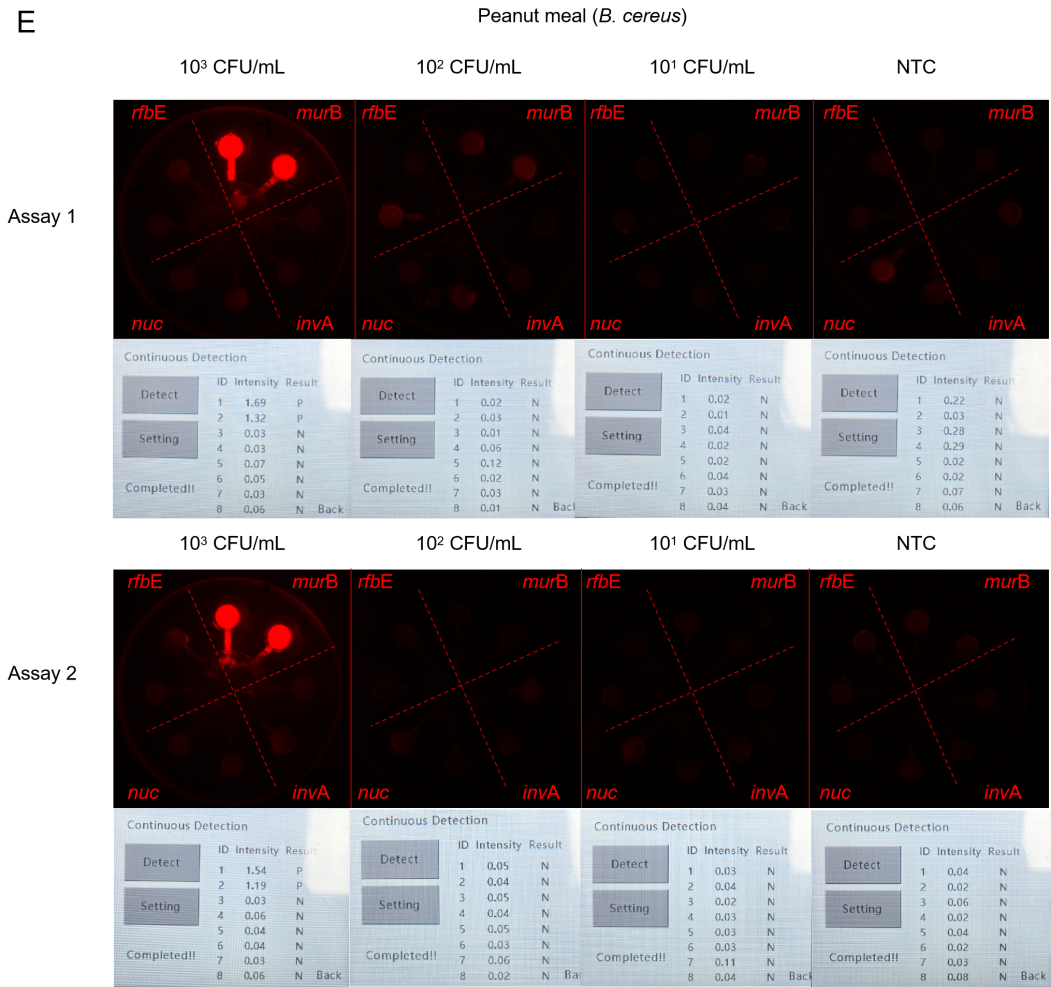


# **Figure S16. The results of disc imaging and portable fluorescence detector for *B. cereus* detection from five types of synthetic food including canola cake (A), soybean meal (B), artificial cream (C), artificial meat (D), and peanut meal (E).**


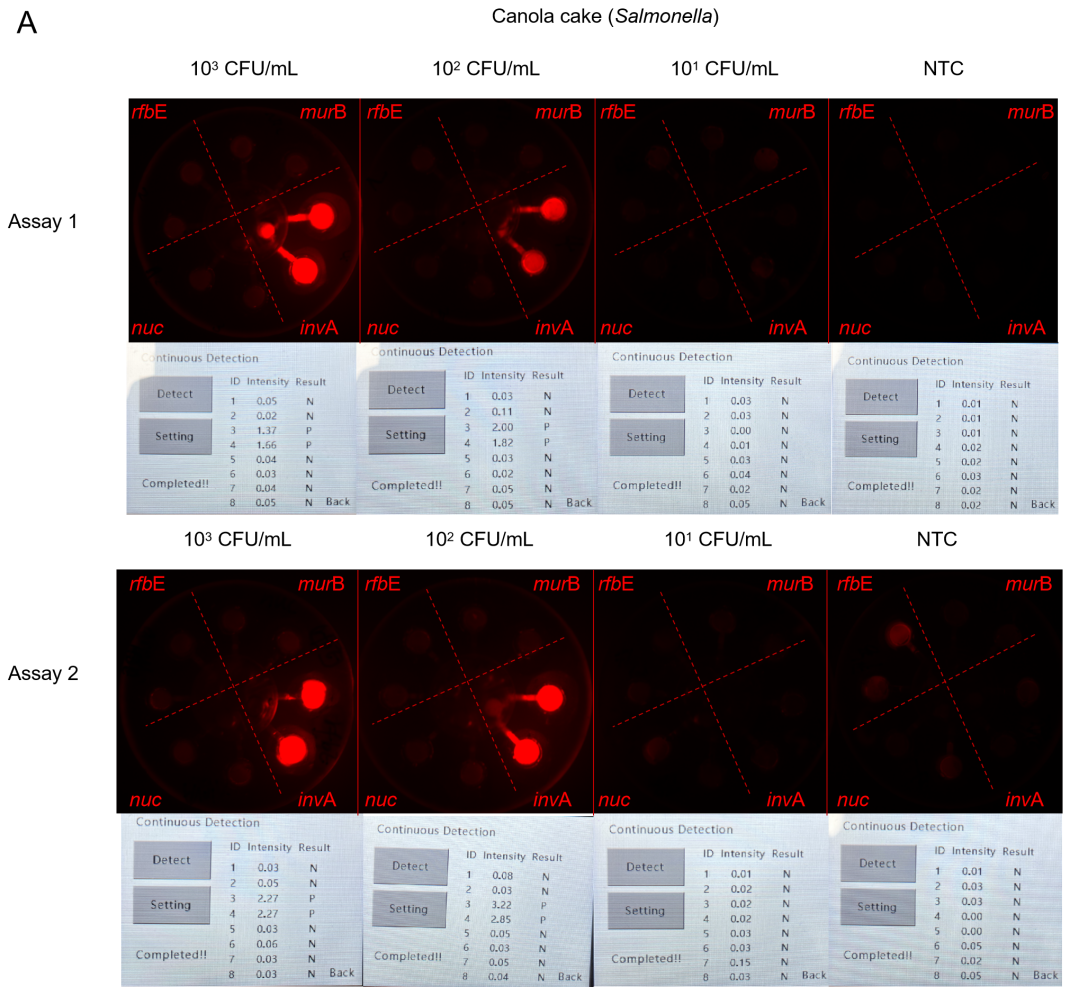


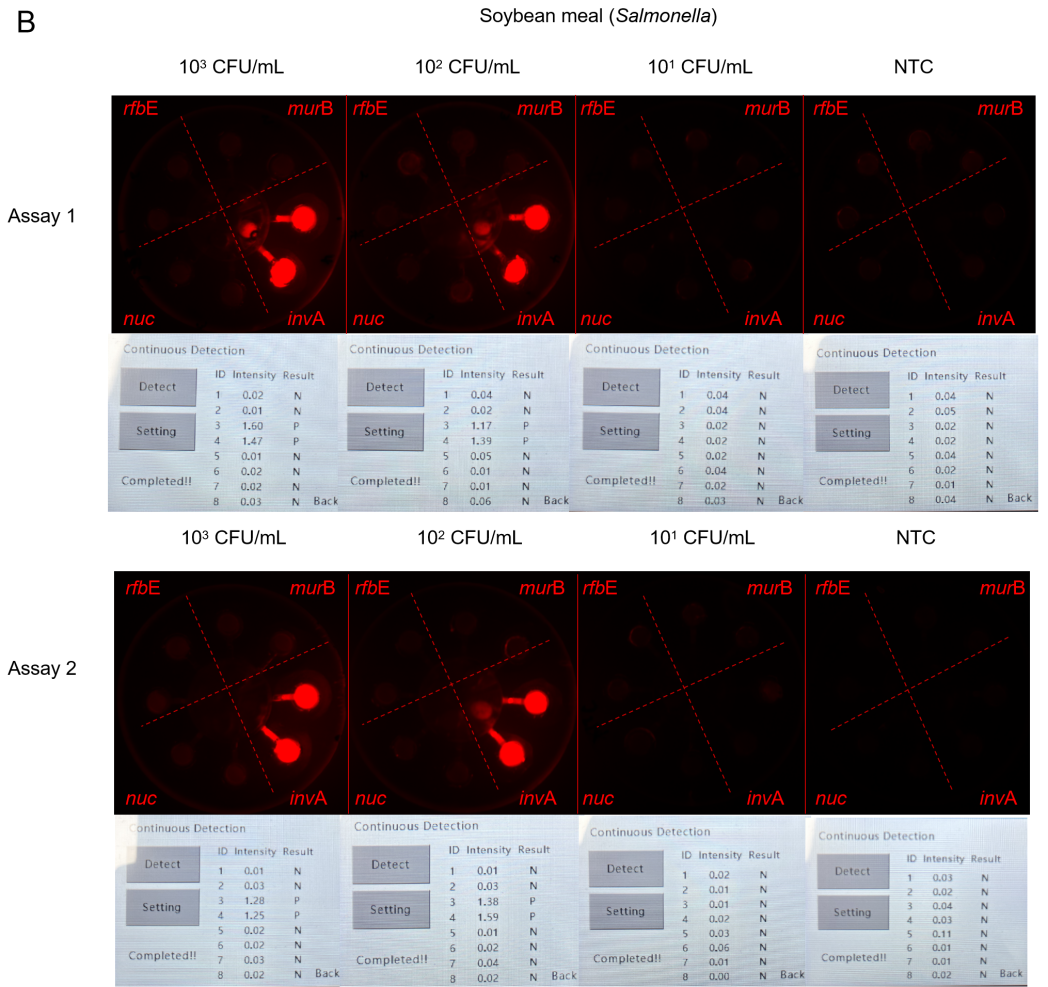


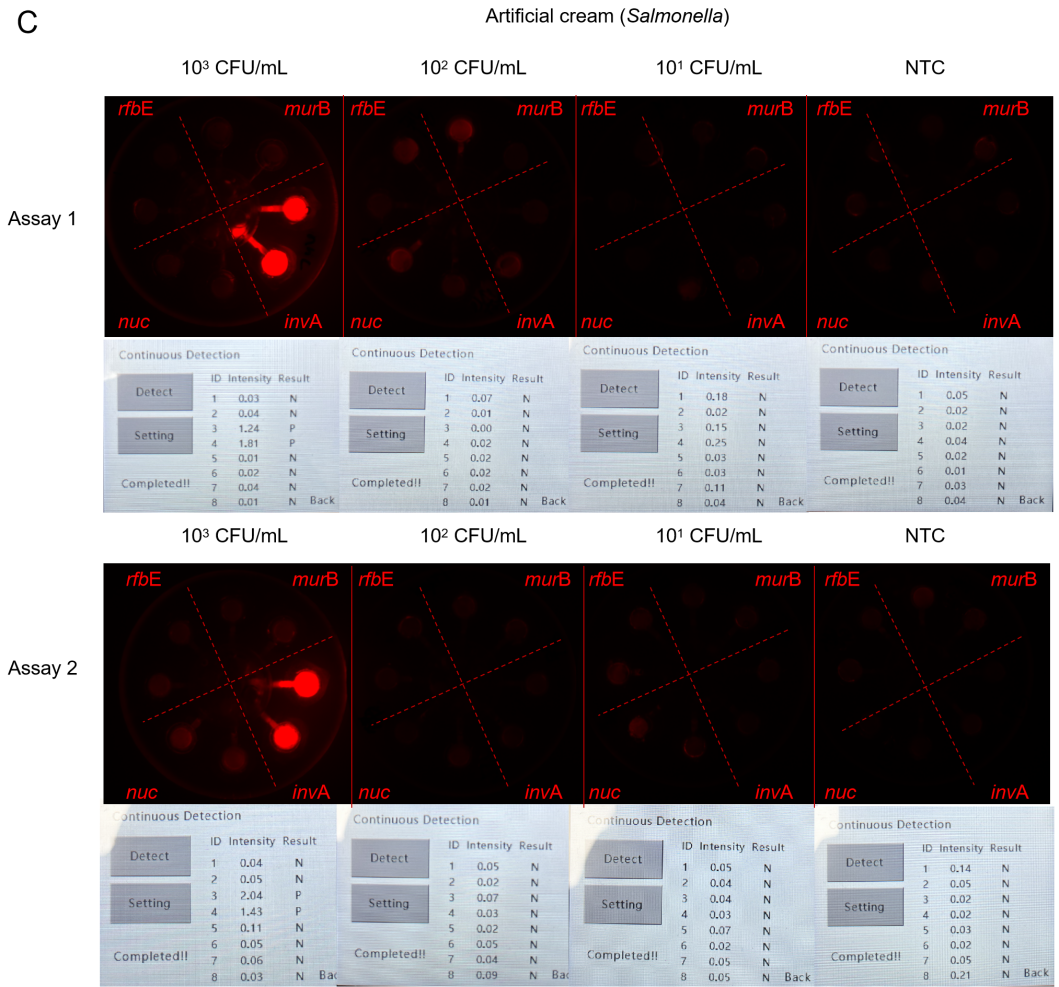


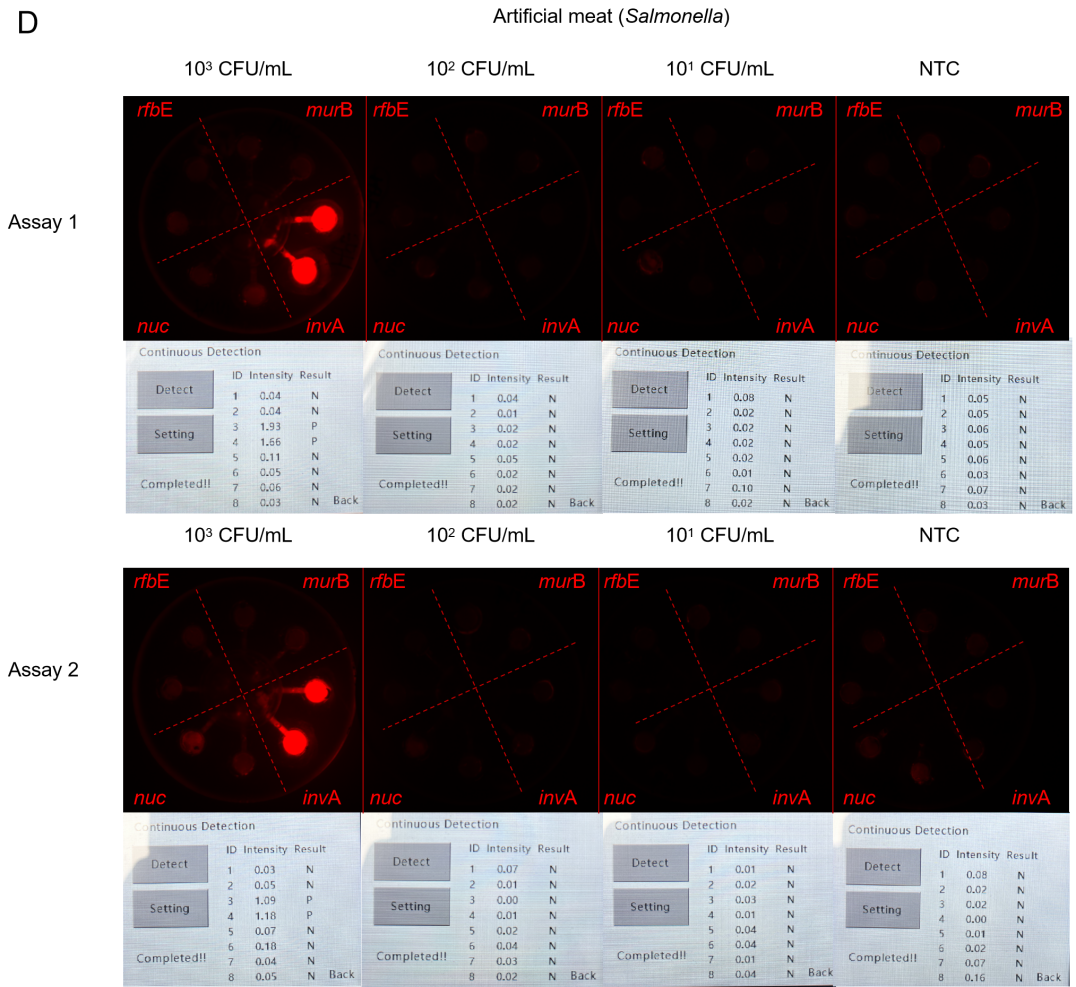


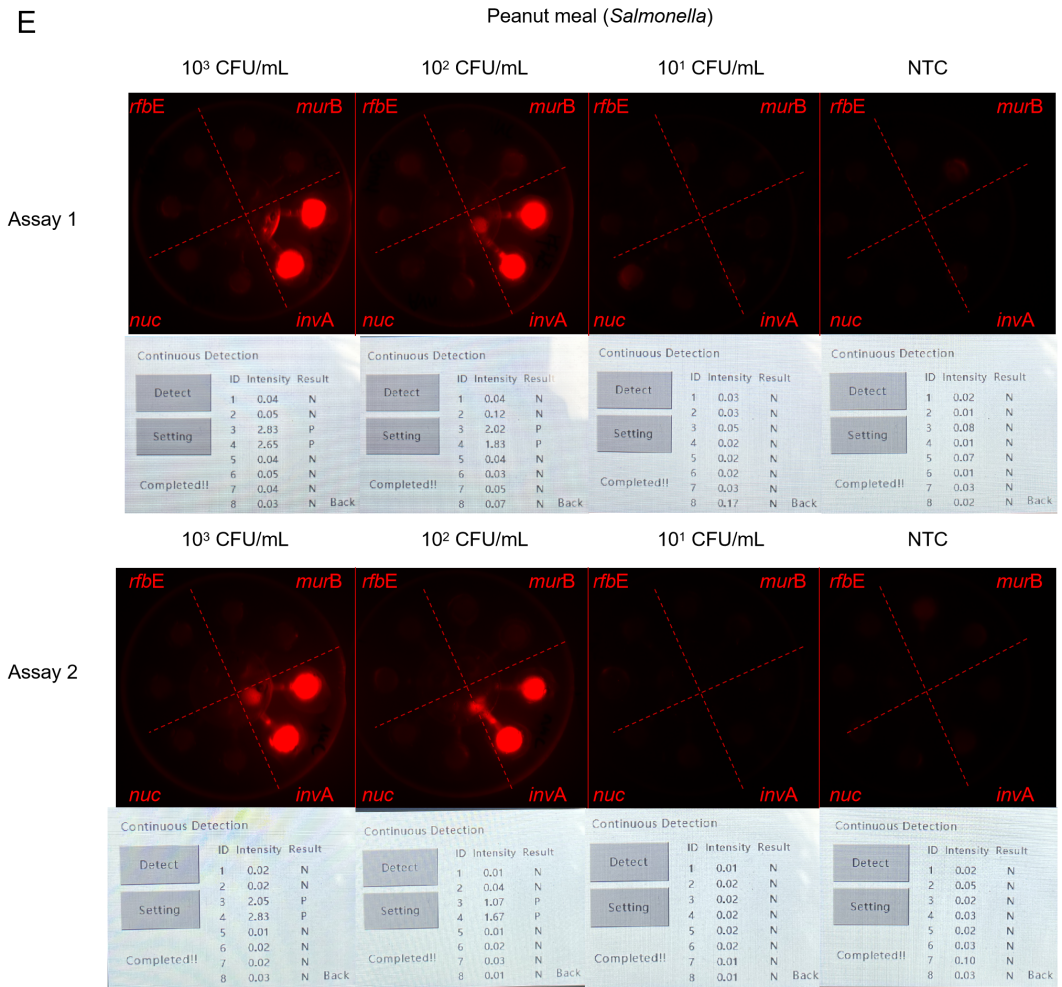


# **Figure S17. The results of disc imaging and portable fluorescence detector for *Salmonella* detection from five types of synthetic food including canola cake (A), soybean meal (B), artificial cream (C), artificial meat (D), and peanut meal (E).**


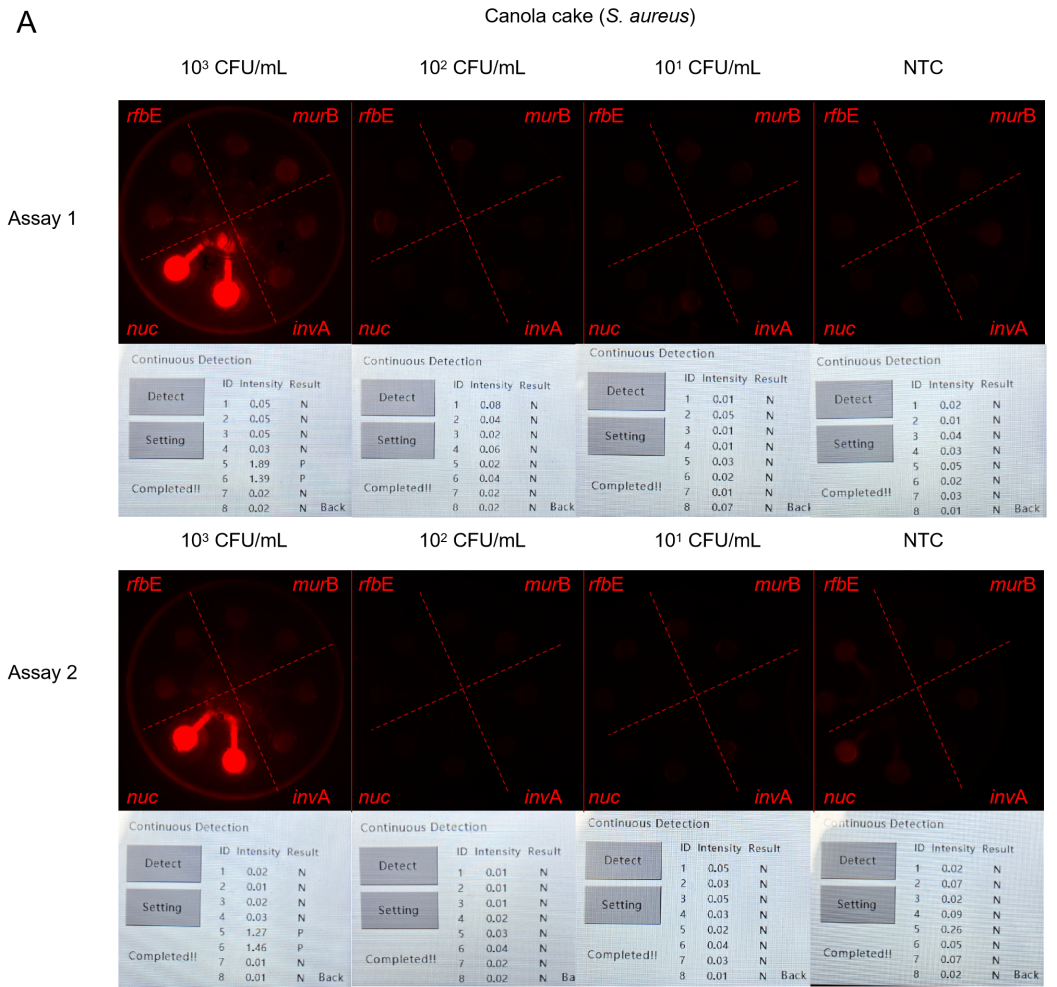


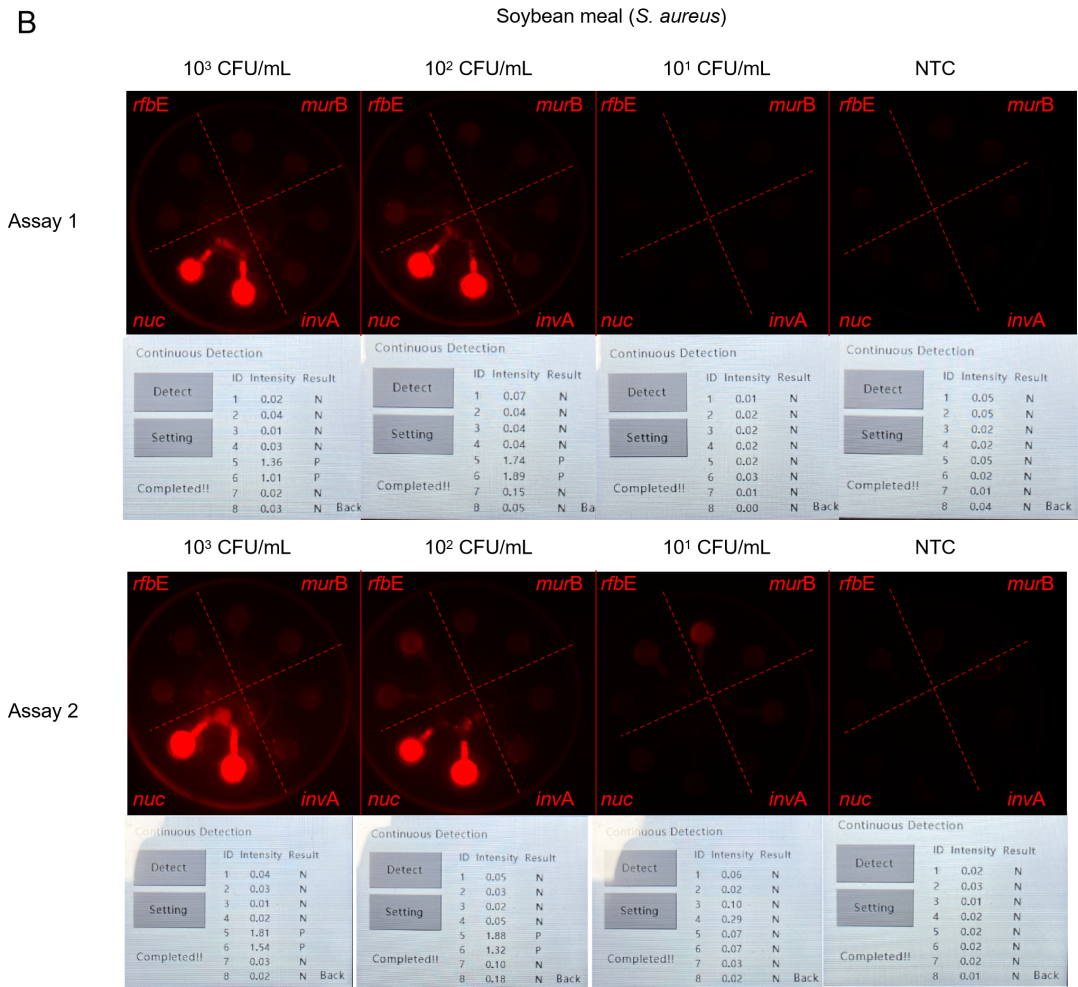


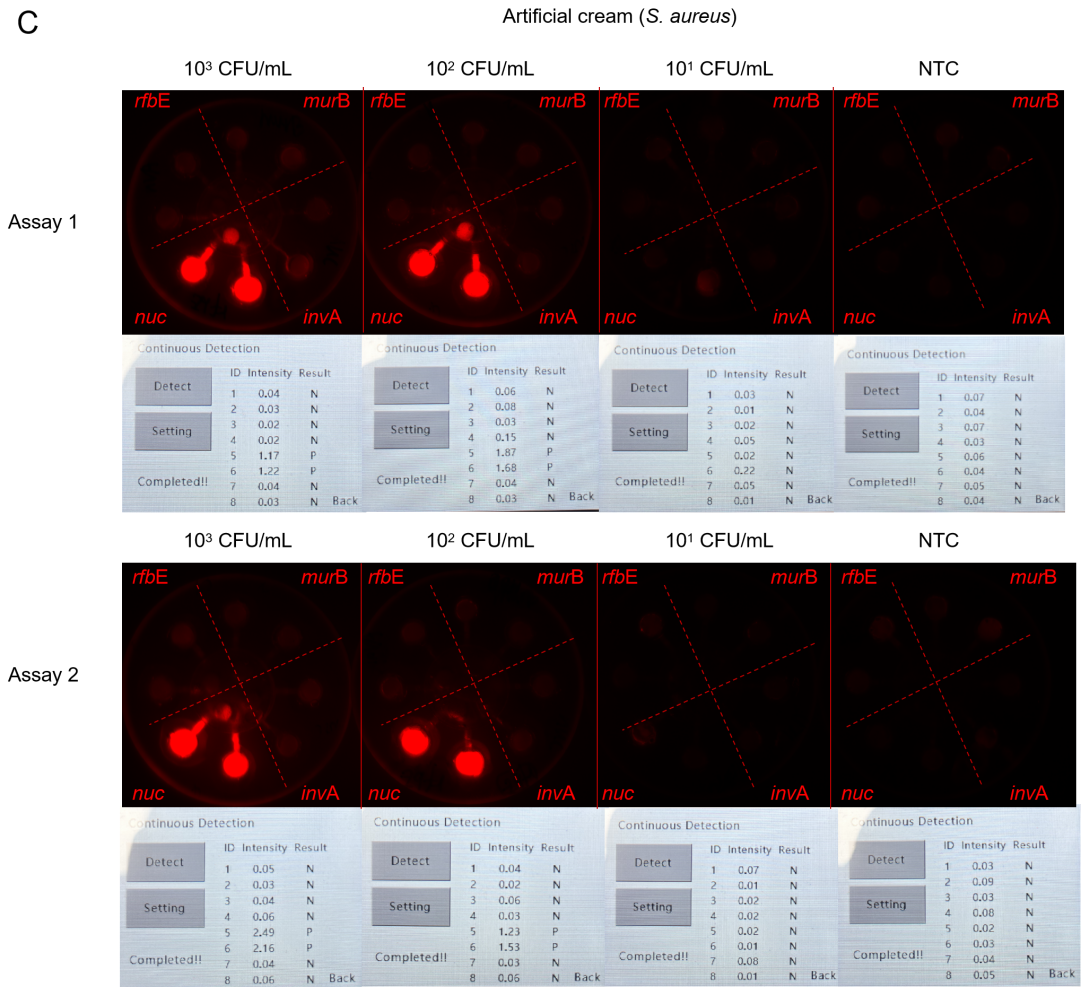


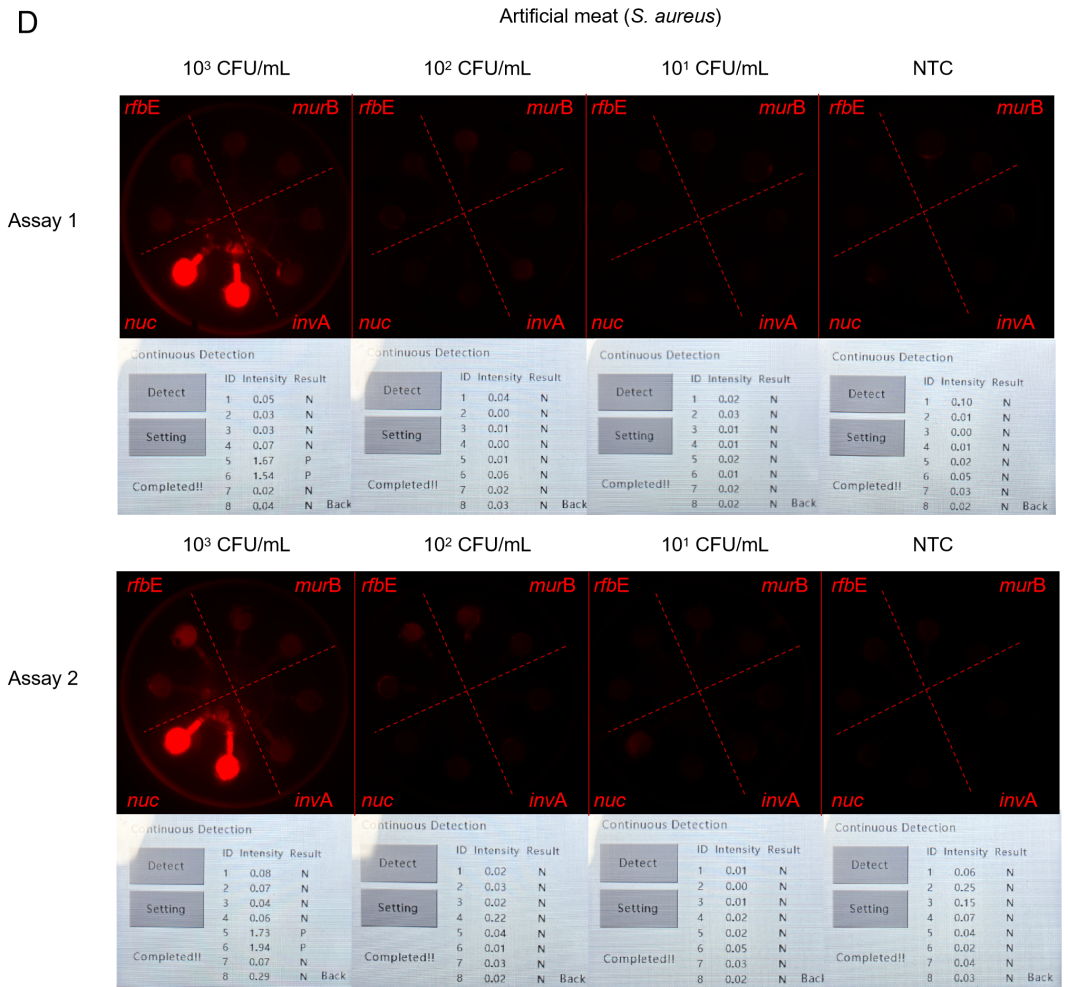


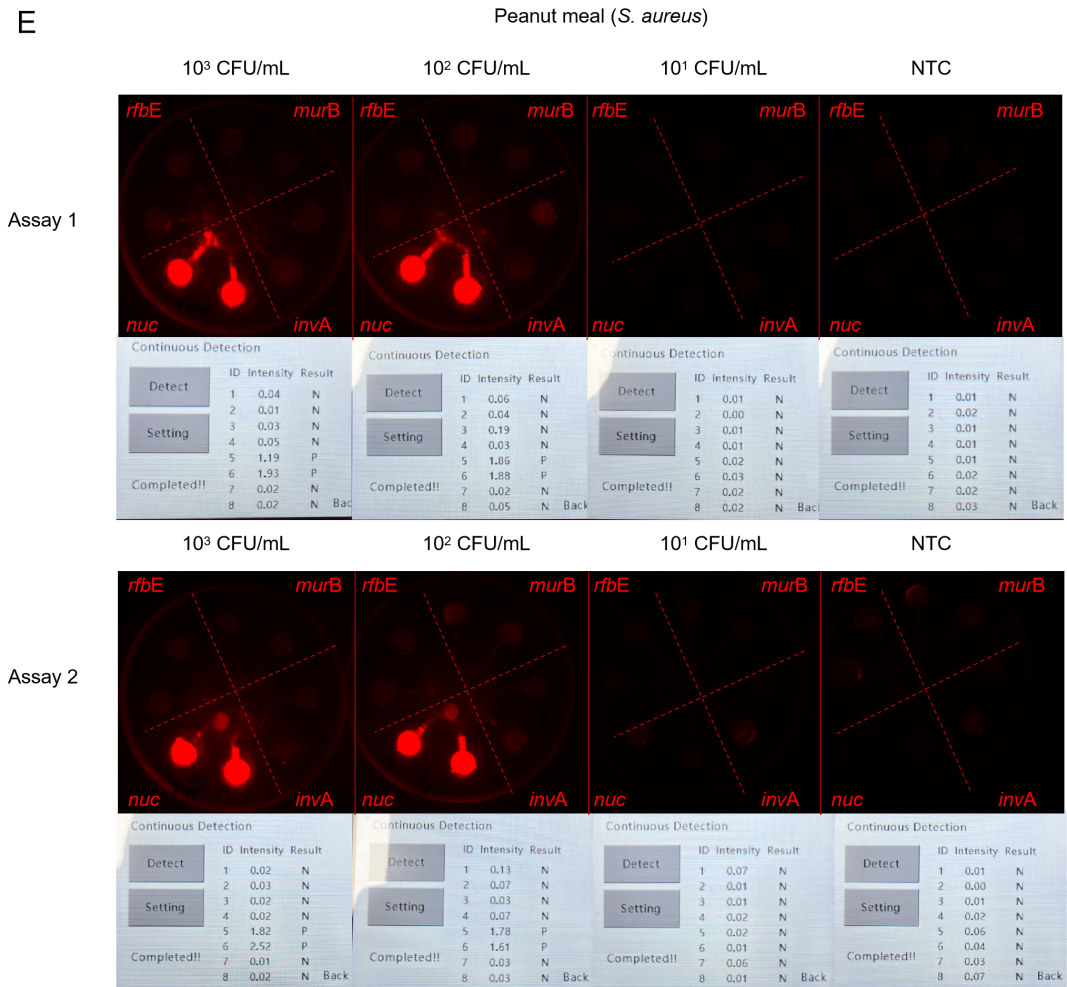


# **Figure S18. The results of disc imaging and portable fluorescence detector for *S. aureus* detection from five types of synthetic food including canola cake (A), soybean meal (B), artificial cream (C), artificial meat (D), and peanut meal (E).**


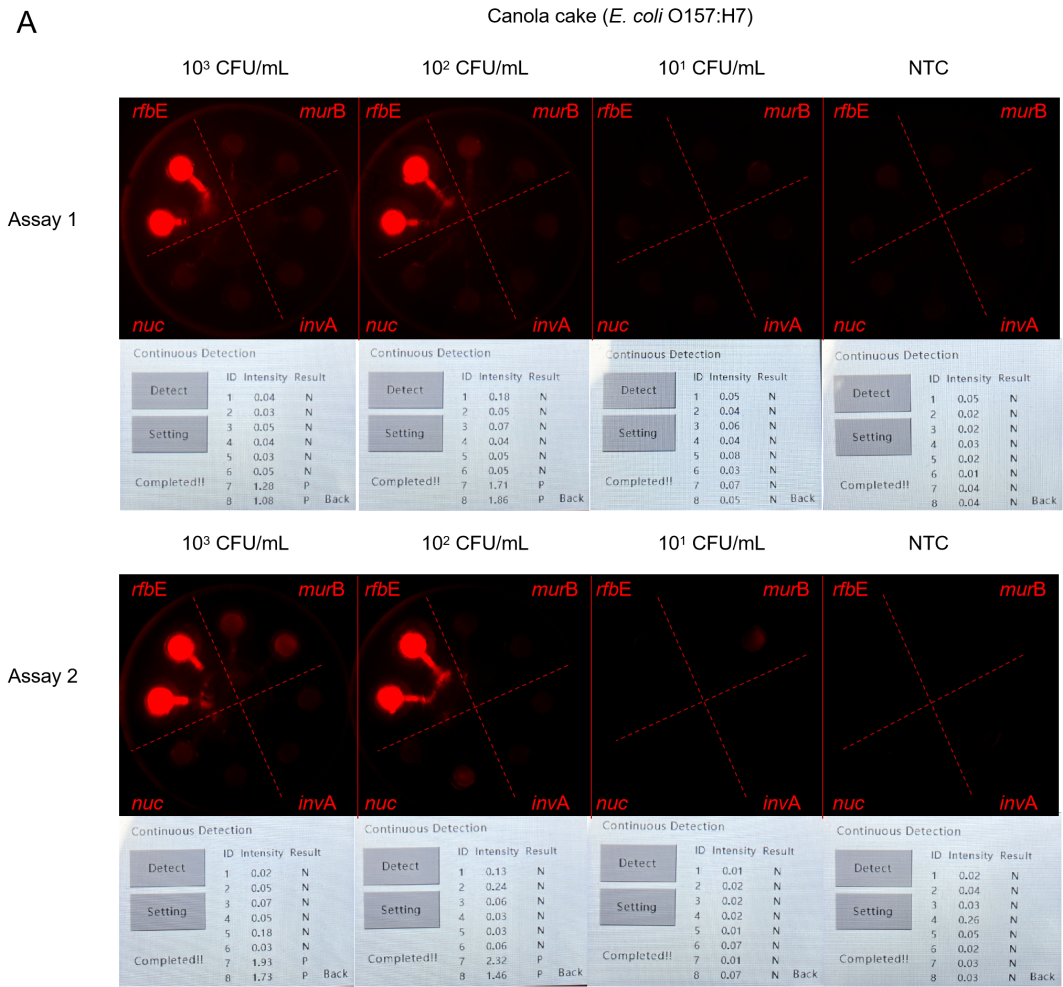


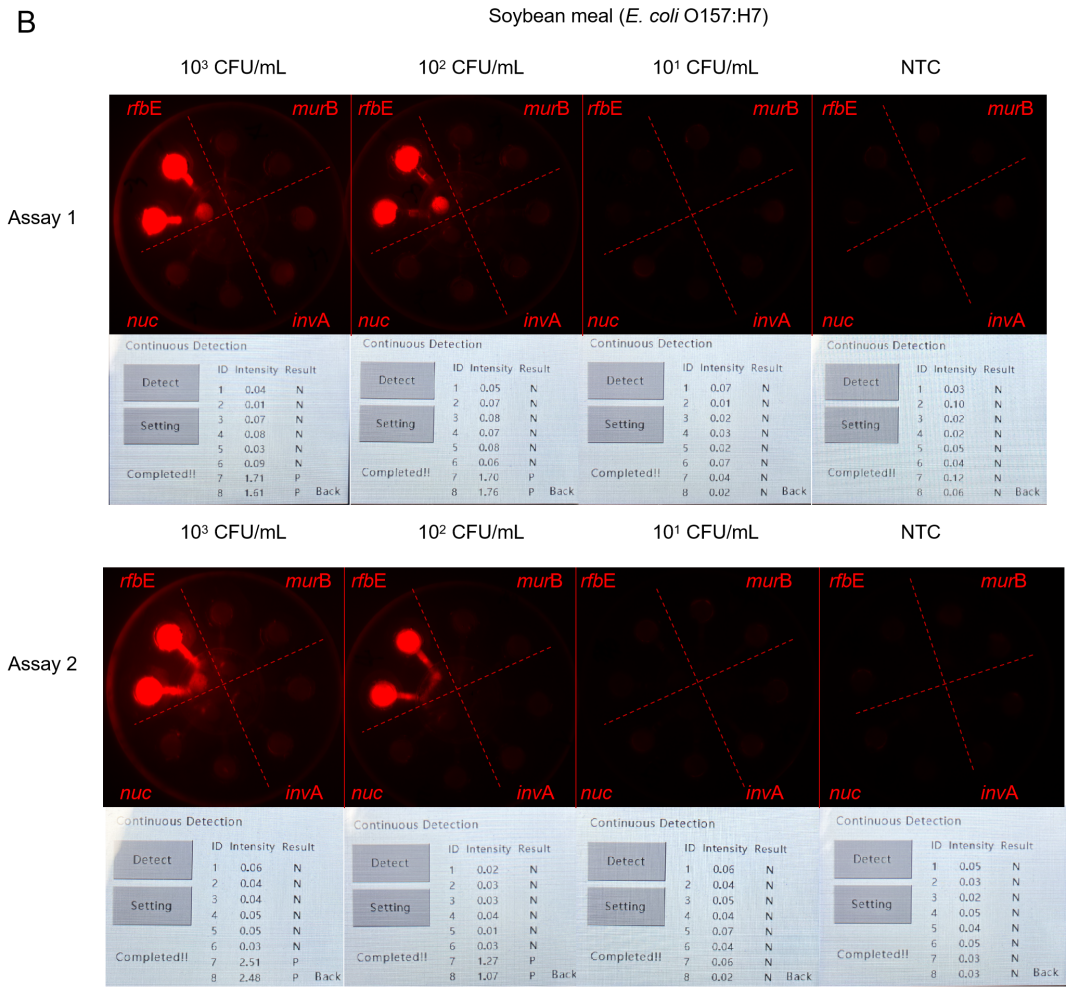


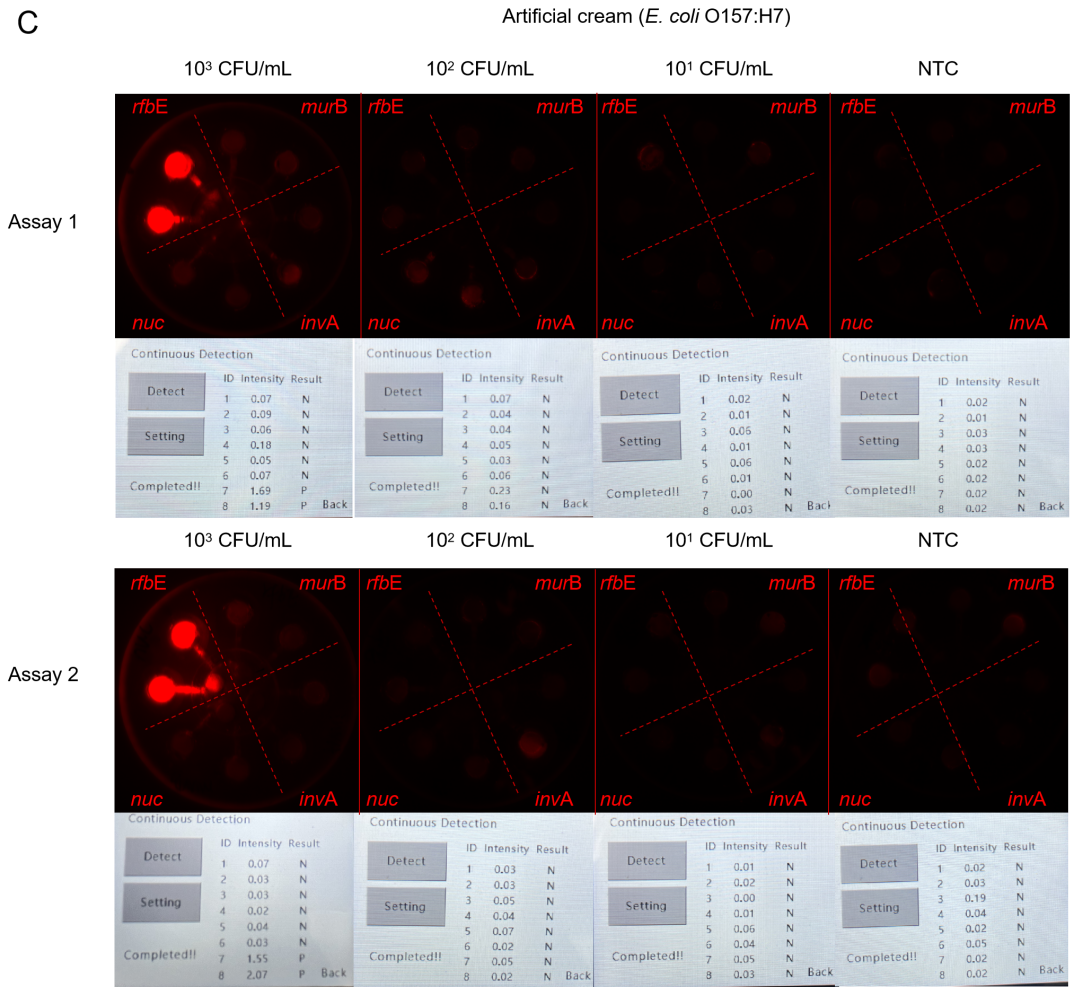


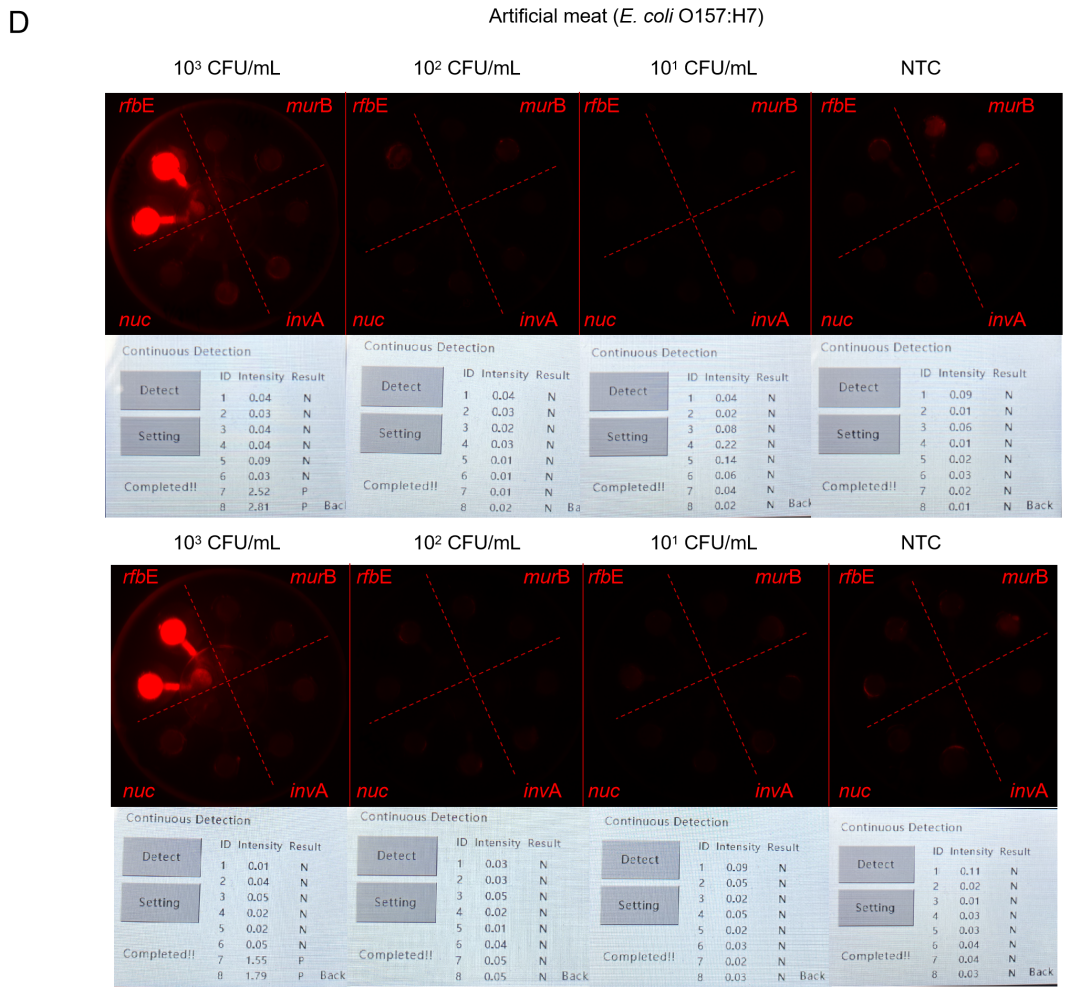


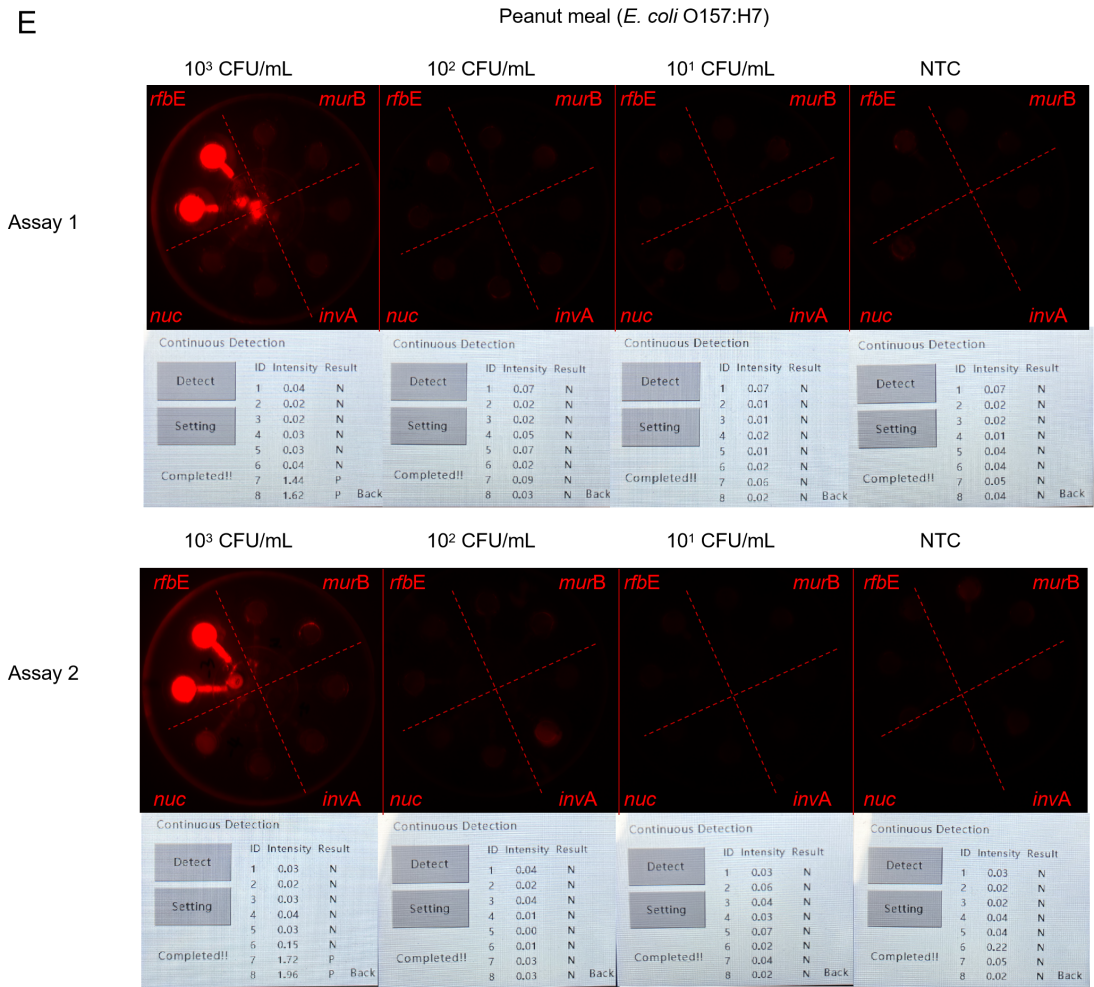


# **Figure S19. The results of disc imaging and portable fluorescence detector for *E. coli* O157:H7 detection from five types of synthetic food including canola cake (A), soybean meal (B), artificial cream (C), artificial meat (D), and peanut meal (E).**


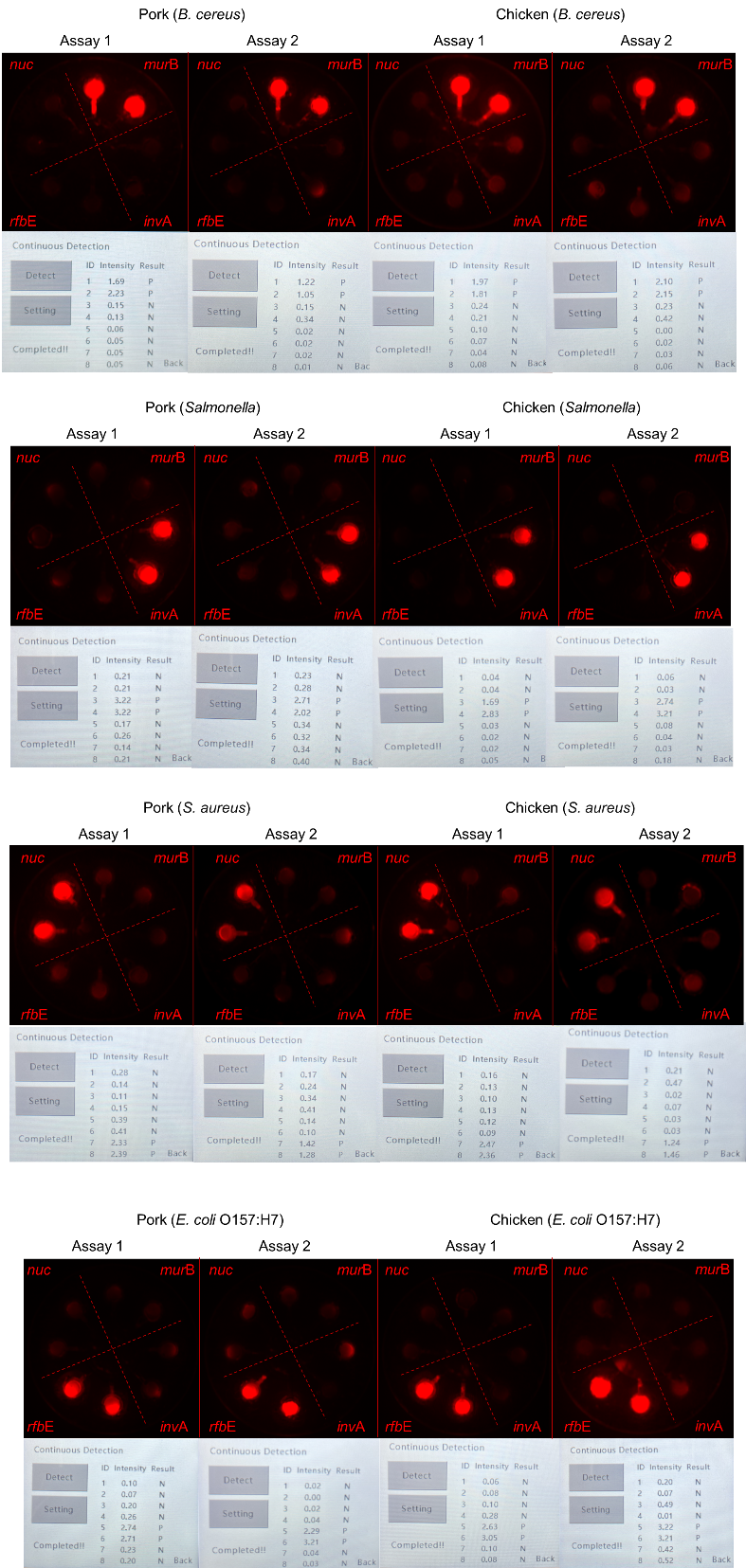


# **Figure S20. The results of disc imaging and portable fluorescence detector for the detection of *B. cereus, Salmonella, S. aureus,* and *E. coli O157:H7* detection from pork and chicken meats.** The meats were spiked with 10^3^ CFU/mL of bacterial cultures.


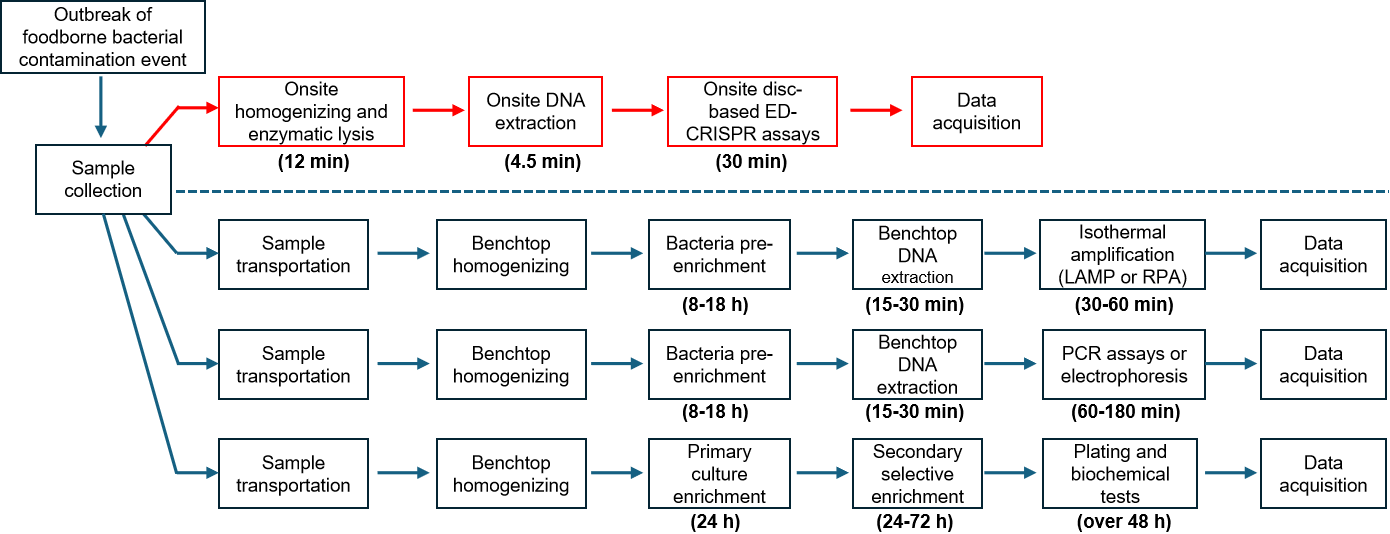


# **Figure S21. Schematic illustration of emergency coping strategies for outbreak of foodborne bacterial contamination event.** The red box shows the new strategy based on onsite EOD-CRISPR detection platform.

# **Table S1. The list of conserved sequences for *B. cereus, Salmonella, E. coli* O157:H7*,* and *S. aureus*.**

| **Target** | **Sequence (5'-3')** |
| --- | --- |
| *B. cereus mur*B gene | TTATTCCTTATCTTCTCCAATAATCCTTACTTCTCGCTCTAACTTCACGCCAAATTTCTCTTCAACTGTCTTTTGTACGAAGTGAATTAAATCGATGTAATCTTGTGCTGTTCCGTTATCAACATTCACCATAAATCCAGCGTGTTTTAAAGAAACTTCCACTCCACCAATTCGCTTACCTTGTAGTCCTGAGTCTTGAATCAACTTACCAGCAAAGTTATTTGGTGGACGCTTAAATACGCTACCACATGAAGGATATTCTAGAGGCTGTTTTGACTCACGCTTAAACGTTAAATCATCCATTTTTGCTTTAATTTCTTCACGTACACCTTCTTCAAGTTCAAATCTCGCTTCAAGAATAATGTAATGGTTGTTAGCAAATACACTCTTACGATATCCAAATTCAAATGCTTCTTTCGTCAAAGTACGTAGCTCTCCATCACCTGTCATTACAACAGCTTCTGTTAACACAAACGATACTTCACCACCGTAAGCACCAGCATTCATATATAACGCTCCGCCAACTGAACCTGGAATACCACAAGCAAACTCAAGACCCGTTAAGTTATGGTCTAAAGCAATACGTGATACGTCAATAATTGCTGCACCACACTGTGCTACAATCGTCGTTCCTGTTACAGTAACACCTGTAATATGAATTAAACTTACTGTAATCCCGCGAATTCCACCATCTTTAATAATGACATTCGATCCATTTCCTAAAAACGTAACTGGAATATTATATTCGTTCGCATATTTGATAACTTCTTGAATTTCATCATAATTTGTAGGCGCAACGAAAACATCTGCTTTTCCGCCAACTTTAATATGCGTATGATTCTTTAACATTTCATCTTGTTTAACATGTCCTTCAGGCAATACCGTACTTAAATATTTATAAACCTCTTGCATATTCAT |
| *Salmonella inv*A gene | CTGGCGGTGGGTTTTGTTGTCTTCTCTATTGTCACCGTGGTCCAGTTTATCGTTATTACCAAAGGTTCAGAACGCGTCGCGGAAGTCGCGGCCCGATTTTCTCTGGATGGTATGCCCGGTAAACAGATGAGTATTGATGCCGATTTGAAGGCCGGTATTATTGATGCGGATGCTGCGCGCGAACGGCGAAGTGTACTGGAAAGGGAAAGCCAGCTTTACGGTTCTTTTGACGGTGCGATGAAGTTTATCAAAGGTGACGCTATTGCCGGCATCATTATTATCTTTGTGAACTTTATTGGCGGTATTTCGGTGGGGATGACCCGCCATGGTATGGATTTGTCCTCCGCCCTGTCTACTTATACCATGCTGACCATTGGTGATGGTCTTGTCGCCCAGATCCCCGCATTGTTGATTGCGATTAGTGCCGGTTTTATCGTGACTCGCGTAAATGGCGATAGCGATAATATGGGGCGGAATATCATGACGCAGCTGTTGAACAACCCATTTGTATTGGTTGTTACGGCTATTTTGACCATTTCAATGGGAACTCTGCCGGGATTCCCGCTGCCGGTATTTGTTATTTTATCGGTGGTTTTAAGCGTACTCTTCTATTTTAAATTCCGTGAAGCAAAACGTAGCGCCGCCAAACCTAAAACCAGTAAAGGCGAGCAGCCGCTCAGTATTGAGGAAAAAGAAGGGTCGTCGTTGGGACTGATTGGCGATCTCGATAAAGTCTCTACAGAGACCGTACCGTTGATATTACTTGTGCCGAAGAGCCGGCGTGAAGATCTGGAAAAAGCTCAACTTGCGGAGCGTCTACGTAGTCAGTTCTTTATTGATTATGGCGTGCGCCTGCCGGAAGTATTGTTACGCGATGGCGAGGGCCTGGACGATAACAGCATCGTATTGTTGATTAATGAGATCCGTGTTGAACAATTTACGGTCTATTTTGATTTGATGCGAGTGGTAAATTATTCCGATGAAGTCGTGTCCTTTGGTATTAATCCAACAATCCATCAGCAAGGTAGCAGTCAGTATTTCTGGGTAACGCATGAAGAGGGGGAGAAACTGCGGGAGCTTGGCTATGTGTTGCGGAACGCGCTTGATGAGCTTTACCACTGTCTGGCGGTGACGCTGGCGCGCAACGTCAATGAATATTTCGGTATTCAGGAAACAAAACATATGCTGGACCAACTGGAAGCGAAATTTCCTGATTTACTTAAAGAAGTGCTCAGACA |
| *E. coli* O157:H7 *rfb*E gene | GGGTTAACTGTTATGTTGTACTGCTTCATTTTTATATATATTGTAAATTACTTTATATGGTATAAATGTAGTTTTAAAAAACATATCGATAGACAGTTAAATATAAGAGGATGAAAATGAAATATATACCAGTTTACCAACCGTCATTGACAGGAAAAGAAAAAGAATATGTAAATGAATGTCTGGACTCAACGTGGATTTCATCAAAAGGAAACTATATTCAGAAGTTTGAAAATAAATTTGCGGAACAAAACCATGTGCAATATGCAACTACTGTAAGTAATGGAACGGTTGCTCTTCATTTAGCTTTGTTAGCGTTAGGTATATCGGAAGGAGATGAAGTTATTGTTCCAACACTGACATATATAGCATCAGTTAATGCTATAAAATACACAGGAGCCACCCCCATTTTCGTTGATTCAGATAATGAAACTTGGCAAATGTCTGTTAGTGACATAGAACAAAAAATCACTAATAAAACTAAAGCTATTATGTGTGTCCATTTATACGGACATCCATGTGATATGGAACAAATTGTAGAACTGGCCAAAAGTAGAAATTTGTTTGTAATTGAAGATTGCGCTGAAGCCTTTGGTTCTAAATATAAAGGTAAATATGTGGGAACATTTGGAGATATTTCTACTTTTAGCTTTTTTGGAAATAAAACTATTACTACAGGTGAAGGTGGAATGGTTGTCACGAATGACAAAACACTTTATGACCGTTGTTTACATTTTAAAGGCCAAGGATTAGCTGTACATAGGCAATATTGGCATGACGTTATAGGCTACAATTATAGGATGACAAATATCTGCGCTGCTATAGGATTAGCCCAGTTAGAACAAGCTGATGATTTTATATCACGAAAACGTGAAATTGCTGATATTTATAAAAAAAATATCAACAGTCTTGTACAAGTCCACAAGGAAAGTAAAGATGTTTTTCACACTTATTGGATGGTCTCAATTCTAACTAGGACCGCAGAGGAAAGAGAGGAATTAAGGAATCACCTTGCAGATAAACTCATCGAAACAAGGCCAGTTTTTTACCCTGTCCACACGATGCCAATGTACTCGGAAAAATATCAAAAGCACCCTATAGCTGAGGATCTTGGTTGGCGTGGAATTAATTTACCTAGTTTCCCCAGCCTATCGAATGAGCAAGTTATTTATATTTGTGAATCTATTAACGAATTTTATAGTGATAAATAGCCTAAAATATTGTAAAGGTCATTCATGAAAATTGCG |
| *S. aureus nuc* gene | GTATGGCAATTGTTTCAATATTACTTATAGGGATGGCTATCAGTAATGTTTCGAAAGGGCAATACGCAAAGAGGTTTTTCTTTTTCGCTACTAGTTGCTTAGTGTTAACTTTAGTTGTAGTTTCAAGTCTAAGTAGCTCAGCAAATGCATCACAAACAGATAACGGCGTAAATAGAAGTGGTTCTGAAGATCCAACAGTATATAGTGCAACTTCAACTAAAAAATTACATAAAGAACCTGCGACATTAATTAAAGCGATTGATGGTGATACGGTTAAATTAATGTACAAAGGTCAACCAATGACATTCAGACTATTATTAGTTGATACACCTGAAACAAAGCATCCTAAAAAAGGTGTAGAGAAATATGGCCCTGAAGCAAGTGCATTTACGAAAAAAATGGTAGAAAATGCAAATAAAATTGAAGTCGAGTTTGACAAAGGTCAAAGAACTGATAAATATGGACGTGGCTTAGCGTATATTTATGCTGATGGAAAAATGGTAAACGAAGCTTTAGTTCGTCAAGGCTTGGCTAAAGTTGCTTATGTTTATAAACCTAACAATACACATGAACAACTTTTAAGAAAAAGTGAAGCACAAGCGAAAAAAGAGAAATTAAATATTTGGAG |

# **Table S2. The list of RPA primers for *B. cereus, Salmonella, E. coli* O157:H7*,* and *S. aureus*.**

| **Target** | **Primer** | **Sequence (5'-3')** |
| --- | --- | --- |
| *B. cereus mur*B gene | Forward primer | ACATTCACCATAAATCCAGCGTGTTTTAAAGA |
|  | Reverse primer | TTTAAGCGTGAGTCAAAACAGCCTCTAG |
| *Salmonella* *inv*A gene | Forward primer | CGTCTACGTAGTCAGTTCTTTATTGATTAT |
|  | Reverse primer | CATCAAATCAAAATAGACCGTAAATTGTTC |
| *E. coli* O157:H7 *rfb*E gene | Forward primer | TTGGATGGTCTCAATTCTAACTAGGACCGCAGA |
|  | Reverse primer | GTGCTTTTGATATTTTTCCGAGTACATTGGCAT |
| *S. aureus* *nuc* gene | Forward primer | GCATCACAAACAGATAACGGCGTAAATAGAAG |
|  | Reverse primer | ACATTAATTTAACCGTATCACCATCAATCGCT |

# **Table S3. The list of crRNA and probe for *B. cereus, Salmonella, E. coli* O157:H7*,* and *S. aureus*.**

| **Target** | **Primer** | **Primer Sequence (5'-3')** |
| --- | --- | --- |
| *B. cereus mur*B gene | crRNA 1 | UAAUUUCUACUAAGUGUAGAUAAGGUAAGCGAAUUGGUGGA |
|  | crRNA 2 | UAAUUUCUACUAAGUGUAGAUACGCUACCACAUGAAGGAUAU |
| *Salmonella* *inv*A gene | crRNA 1 | UAAUUUCUACUAAGUGUAGAUACUUCCGGCAGGCGCACGCC |
|  | crRNA 2 | UAAUUUCUACUAAGUGUAGAUUGAUUAAUGAGAUCCGUGU |
| *E. coli* O157:H7 *rfb*E gene | crRNA 1 | UAAUUUCUACUAAGUGUAGAUCAAGGUGAUUCCUUAAUUCCUC |
|  | crRNA 2 | UAAUUUCUACUAAGUGUAGAUAACAAGGCCAGUUUUUUACC |
| *S. aureus* *nuc* gene | crRNA 1 | UAAUUUCUACUAAGUGUAGAUAAGUUGCACUAUAUACUGUUGG |
|  | crRNA 2 | UAAUUUCUACUAAGUGUAGAUAUUACAUAAAGAACCUGCGA |
| Probe |  | Cy5-TTTTTT-BHQ2 |

# **Table S4. The list of PCR primers for *B. cereus, Salmonella, E. coli* O157:H7*,* and *S. aureus*.**

| Target | Primer | Primer Sequence (5'-3') | Source |
| --- | --- | --- | --- |
| *B. cereus mur*B gene | Forward primer | CCTTCTTCAAGTTCAAATCTCG | SN/T 3932-2014 |
|  | Reverse primer | GTTGTAATGACAGGTGATGGA |  |
| *Salmonella* *inv*A gene | Forward primer | TCGCACCGTCAAAAGAACCGTAAAGC | SN/T 5439.1-2022 |
|  | Reverse primer | GCATTATCGATCAGTACCAGTCGTCT |  |
| *E. coli* O157:H7 *rfb*E gene | Forward primer | CGGACATCCATGTGATATGG | This study |
|  | Reverse primer | TTGCCTATGTACAGCTAATCC |  |
| *S. aureus* *nuc* gene | Forward primer  Reverse primer | AGCATCCTAAAAAAGGTGTAGAGA | SN/T 5364.5-2021 |
|  | Reverse primer | CTTCAATTTTATTTGCATTTTCTACCA |  |

# **Table S5. The Cq values of the qPCR method for detecting *B. cereus.***

| Target | Concentration (CFU/mL) | Cq of Assay 1 | Cq of Assay 2 | Cq of Assay 3 | Average Cq ^a^ |
| --- | --- | --- | --- | --- | --- |
| Canola cake | 10^3^ | 28.3 | 29.22 | 30.16 | 29.23 |
|  | 10^2^ | 30.15 | 30.99 | 28.87 | 30.00 |
|  | 10^1^ | Negative | Negative | Negative | Negative |
|  | NTC | Negative | Negative | Negative | Negative |
| Soybean meal | 10^3^ | 30.54 | 29.81 | 29.48 | 29.94 |
|  | 10^2^ | 30.48 | Negative | 31.1 | Negative |
|  | 10^1^ | Negative | Negative | Negative | Negative |
|  | NTC | Negative | Negative | Negative | Negative |
| Artificial cream | 10^3^ | 29.17 | 29.2 | 28.88 | 29.06 |
|  | 10^2^ | 31.83 | 31.41 | 29.52 | 30.92 |
|  | 10^1^ | Negative | Negative | Negative | Negative |
|  | NTC | Negative | Negative | Negative | Negative |
| Artificial meat | 10^3^ | 29.45 | 28.76 | 29.37 | 29.19 |
|  | 10^2^ | 30.72 | 30.01 | 29.49 | 30.07 |
|  | 10^1^ | Negative | Negative | Negative | Negative |
|  | NTC | Negative | Negative | Negative | Negative |
| Peanut meal | 10^3^ | 28.61 | 28.9 | 28.35 | 28.62 |
|  | 10^2^ | 30.21 | 30.46 | 29.77 | 30.15 |
|  | 10^1^ | Negative | Negative | Negative | Negative |
|  | NTC | Negative | Negative | Negative | Negative |

^a^ The reaction with Cq≥32 was defined as negative while the positive was defined when all replicates presented Cq<32.

# **Table S6. The Cq values of the qPCR method for detecting *Salmonella.***

| Target | Concentration (CFU/mL) | Cq of Assay 1 | Cq of Assay 2 | Cq of Assay 3 | Average Cq ^a^ |
| --- | --- | --- | --- | --- | --- |
| Canola cake | 10^3^ | 29.66 | 29.36 | 29.95 | 29.66 |
|  | 10^2^ | 33.51 | Negative | 33.68 | Negative |
|  | 10^1^ | Negative | Negative | 34.19 | Negative |
|  | NTC | Negative | Negative | Negative | Negative |
| Soybean meal | 10^3^ | 28.84 | 29.9 | 29.58 | 29.44 |
|  | 10^2^ | 32.03 | 31.48 | 33.42 | 32.31 |
|  | 10^1^ | Negative | Negative | Negative | Negative |
|  | NTC | Negative | Negative | Negative | Negative |
| Artificial cream | 10^3^ | 29.13 | 29.28 | 29.9 | 29.44 |
|  | 10^2^ | Negative | 34.73 | 31.81 | Negative |
|  | 10^1^ | Negative | Negative | Negative | Negative |
|  | NTC | Negative | Negative | Negative | Negative |
| Artificial meat | 10^3^ | 29.58 | 30.25 | 29.48 | 29.77 |
|  | 10^2^ | 33.91 | 32.76 | 31.67 | 32.78 |
|  | 10^1^ | Negative | 33.52 | Negative | Negative |
|  | NTC | Negative | Negative | Negative | Negative |
| Peanut meal | 10^3^ | 30.43 | 29.68 | 29.53 | 29.88 |
|  | 10^2^ | Negative | Negative | 33.18 | Negative |
|  | 10^1^ | Negative | Negative | Negative | Negative |
|  | NTC | Negative | Negative | Negative | Negative |

^a^ The reaction with Cq≥35 was defined as negative while the positive was defined when all replicates presented Cq<35.

# **Table S7. The Cq values of the qPCR method for detecting *S. aureus*.**

| Target | Concentration (CFU/mL) | Cq of Assay 1 | Cq of Assay 2 | Cq of Assay 3 | Average Cq ^a^ |
| --- | --- | --- | --- | --- | --- |
| Canola cake | 10^3^ | 28.2 | 28.67 | 28.65 | 28.51 |
|  | 10^2^ | 28.79 | 30.08 | 29.93 | 29.6 |
|  | 10^1^ | Negative | Negative | Negative | Negative |
|  | NTC | Negative | Negative | Negative | Negative |
| Soybean meal | 10^3^ | 28.92 | 28.04 | 28.97 | 28.64 |
|  | 10^2^ | 28.62 | 30.69 | 30.94 | 30.08 |
|  | 10^1^ | Negative | 30.73 | Negative | Negative |
|  | NTC | Negative | Negative | Negative | Negative |
| Artificial cream | 10^3^ | 28.54 | 29.09 | 28.99 | 28.87 |
|  | 10^2^ | 28.63 | 30.66 | 28.96 | 29.42 |
|  | 10^1^ | Negative | 31.12 | Negative | Negative |
|  | NTC | Negative | Negative | Negative | Negative |
| Artificial meat | 10^3^ | 29.04 | 28.49 | 29.24 | 28.92 |
|  | 10^2^ | 27.77 | 30.97 | 29.88 | 29.54 |
|  | 10^1^ | 32.16 | 30.99 | Negative | Negative |
|  | NTC | Negative | Negative | Negative | Negative |
| Peanut meal | 10^3^ | 28.14 | 28.73 | 28.6 | 28.49 |
|  | 10^2^ | 29.99 | 30.75 | 31.17 | 30.64 |
|  | 10^1^ | Negative | Negative | Negative | Negative |
|  | NTC | Negative | Negative | Negative | Negative |

^a^ The reaction with Cq≥35 was defined as negative while the positive was defined when all replicates presented Cq<35.

# **Table S8. The Cq values of the qPCR method for detecting *E. coli* O157:H7.**

| Target | Concentration (CFU/mL) | Cq of Assay 1 | Cq of Assay 2 | Cq of Assay 3 | Average Cq ^a^ |
| --- | --- | --- | --- | --- | --- |
| Canola cake | 10^3^ | 30.64 | 29.35 | 30.09 | 30.03 |
|  | 10^2^ | 29.35 | 31.89 | 32.7 | 31.31 |
|  | 10^1^ | Negative | Negative | Negative | Negative |
|  | NTC | Negative | Negative | Negative | Negative |
| Soybean meal | 10^3^ | 28.87 | 28.41 | 31.42 | 29.57 |
|  | 10^2^ | 32.69 | 31.44 | 30.62 | 31.58 |
|  | 10^1^ | Negative | Negative | Negative | Negative |
|  | NTC | Negative | Negative | Negative | Negative |
| Artificial cream | 10^3^ | 31.2 | 31.62 | 31.24 | 31.35 |
|  | 10^2^ | Negative | Negative | 30.78 | Negative |
|  | 10^1^ | Negative | Negative | Negative | Negative |
|  | NTC | Negative | Negative | Negative | Negative |
| Artificial meat | 10^3^ | 30.55 | 30.66 | 30.31 | 30.51 |
|  | 10^2^ | Negative | 31.12 | 31.5 | Negative |
|  | 10^1^ | Negative | Negative | Negative | Negative |
|  | NTC | Negative | Negative | Negative | Negative |
| Peanut meal | 10^3^ | 31.09 | 31.05 | 29.57 | 30.57 |
|  | 10^2^ | 30.74 | 31.79 | 31.5 | 31.34 |
|  | 10^1^ | Negative | Negative | 30.43 | Negative |
|  | NTC | Negative | Negative | Negative | Negative |

^a^ The reaction with Cq≥33 was defined as negative while the positive was defined when all replicates presented Cq<33.

# **Table S9. Comparison of currently reported one-pot RPA-CRISPR/12a assays with the EOD-CRISPR assay.**

| **Assay Name** | **Targets** | **Cas Effector** | **Number of crRNA** | **Efficiency of sensing ^a^** | **Assay time** | **Sensitivity** | **Ref.** |
| --- | --- | --- | --- | --- | --- | --- | --- |
| EOD-CRISPR | Foodborne pathogens | uAsCas12a | 2 | Super high | 10-20 min | 1 copy or 10^-4^-10^-5^ ng per test | This study |
| deCOViD | SARS-CoV-2 | LbCas12a | 2 | High | 15-30 min | 10 copies per test | [1] |
| CRISPR gel biosensing platform | HIV viral | LbCas12a | 1 | Moderate | 30 min | 30 copies per test | [2] |
| AIOD-CRISPR | SARS-CoV-2 | LbCas12a | 2 | High | 20-40 min | 2 copies per test | [3] |
| Cas12a-MPR | *Listeria species* | LbCas12a | 1 | Moderate | 50 min | About 60.2 copies per test | [4] |
| OPRCC-eLFS | *Salmonella* | LbaCas12a | 1 | Moderate | 90 min | 3.84 CFU/mL | [5] |
| RADICA | SARS-CoV-2 | LbCas12a | 1 | Moderate | 60 min | 6 copies per test | [6] |

a The efficiency of sensing is reflected by the real-time fluorescence change of positive reaction.

# **References**

[1] J. S. Park, K. Hsieh, L. Chen, A. Kaushik, A. Y. Trick, T. H. Wang, *Adv Sci (Weinh)* **2021**, *8* (5), 2003564.

[2] N. Uno, Z. Li, L. Avery, M. M. Sfeir, C. Liu, *Anal Chim Acta* **2023**, *1262*, 341258.

[3] X. Ding, K. Yin, Z. Li, C. Liu, *bioRxiv* **2020**.

[4] Y. Tian, T. Liu, C. Liu, Q. Xu, S. Fang, Y. Wu, M. Wu, Q. Liu, *LWT* **2021**, *152*, 112166.

[5] Q. Chen, H. Wang, H. Xu, Y. Peng, B. Yao, Z. Chen, J. Yang, S. Adeloju, W. Chen, *Biosensors and Bioelectronics* **2025**, 117529.

[6] X. Wu, J. K. Tay, C. K. Goh, C. Chan, Y. H. Lee, S. L. Springs, Y. Wang, K. S. Loh, T. K. Lu, H. Yu, *Biomaterials* **2021**, *274*, 120876.
